# Drops in confidence precede symptoms of obsessive-compulsive disorder

**DOI:** 10.1038/s41467-026-76671-5

**Published:** 2026-08-13

**Authors:** A. A. Marzuki, L. A. Kosina, L. Dome, S. R. C. Hewitt, T. U. Hauser

**Affiliations:** 1 Department of Psychiatry and Psychotherapy, Faculty of Medicine, Tübingen University, Tübingen, Germany; 2 German Center for Mental Health (DZPG), Tübingen, Germany; 3 Department of Neuroimaging, Institute of Psychology, Psychiatry & Neuroscience, King’s College London, London, UK; 4 Max Planck UCL Centre for Computational Psychiatry and Ageing Research, University College London, London, UK; 5 Functional Imaging Laboratory, Department of Imaging Neuroscience, University College London, London, UK

**Keywords:** Human behaviour, Decision

## Abstract

Mental health symptoms, like those in obsessive-compulsive disorder, show pronounced fluctuations over time. However, little is known about the underlying factors driving these fluctuations. Whilst theoretical accounts propose that cognitive processes drive symptom variability, cross-sectional work is unable to effectively probe these mechanisms. Based on prevailing notions of doubt and underconfidence being cognitive hallmarks of the disorder, we investigate whether metacognitive processes correspond to obsessive-compulsive disorder symptoms. We employ a smartphone-based momentary assessment design wherein participants with obsessive-compulsive disorder (*N* = 137) log instances of experiencing symptoms across 2-weeks. Participants also complete daily scheduled assessments and play a perceptual metacognitive task every two days. Our results reveal that metacognition is associated with obsessive-compulsive symptoms. Concretely, we find directional effects whereby reductions in both self-confidence and task-based metacognition precede occurrences of obsessive-compulsive symptom logs, but not vice versa. Moreover, confidence during perceptual decision-making is significantly related to self-reported confidence on a moment-to-moment basis, indicating that distinct metacognitive modalities may be interrelated. Results remain robust in the face of several sensitivity analyses targeting possible imbalances and missingness in the data. Thus, our micro-longitudinal study design provides evidence for cognitive distortions (both self-reported and task-based) preceding symptom escalations, signifying the potential for designing just-in-time interventions.

## Abstract (web summary)

To devise effective interventions for obsessive-compulsive disorder, it is crucial to understand antecedents to symptom emergence. Here, the authors show evidence for self- and decision making underconfidence preceding symptom escalations.

## Introduction

Variability is a fundamental aspect of human nature. Our moods, feelings, as well as cognitive attitudes fluctuate along different timescales, from minutes^1^ to hours^2^ to days and months^3^. This is also apparent in mental illnesses, where symptoms and their severity wax and wane seemingly unpredictably. Obsessive-compulsive disorder (OCD), for example, is known to show symptom fluctuations even within a single day^4^. Yet, the factors influencing these fluctuations remain unclear. This is a crucial challenge to be addressed, because pinpointing when symptoms are likely to get worse is essential for providing just-in-time support.

A prominent account potentially explaining symptom fluctuations stems from cognitive behavioural therapy^5^, where it is believed that symptoms arise (or fluctuate) due to biased cognitive patterns. This suggests that a worsening in cognitive biases precedes and drives mental illness symptoms—a notion that has led to extensive research investment into characterising cognitive mechanisms underlying the disorder^6–8^. However, little is known about whether these cognitive mechanisms underlie more rapid fluctuations in symptoms on a day-to-day basis.

In this study, we put this hypothesis to the test by investigating fluctuations in OCD symptoms. To this end, we focused on metacognition as a candidate cognitive predictor. Metacognition refers to introspection into and confidence over our thoughts and/or cognitive processes^9,10^ and has been linked to ensuing moods and feelings^11^. Importantly, metacognition is believed to be altered in OCD^12–14^, which is thought to drive intrusive thoughts and compulsions. Empirically, lowered confidence is correlated with dysfunctional and elevated levels of checking (a form of compulsive behaviour) in clinical OCD^15^. Based on these cross-sectional and group-level findings, it is imperative to further investigate how metacognitive fluctuations correspond meaningfully to variance and severity in OCD symptoms

In the current study, we investigated whether rapid fluctuations in metacognition (i.e. changes within a few hours) were associated with ebbs and flows in OCD symptoms. To do so, we have built on our recently established ecological momentary cognitive testing (EMCT) approach^2^, where we trace state fluctuations in feelings, symptoms and cognition by repeatedly sampling participants’ behaviours and experiences in real-world settings.

To capture OCD symptoms in this study, we evaluated instances where participants with OCD self-reported experiencing symptoms using a 2-week smartphone protocol. We show that these daily 'symptom logs' are substantively associated with relevant states and external contexts. Further, to address an ongoing discussion over the best metrics for assessing metacognition^16^, we evaluated metacognition using multiple methods, including self-reported confidence and task-based judgements. In our micro-longitudinal smartphone-based design in 137 participants, we find that both measures of metacognition are indeed related to OCD symptoms. Moreover, we demonstrate a clear directional effect, wherein drops in metacognition precede OCD symptoms. Our findings thus provide direct evidence for fluctuations in (meta-)cognition driving mental health symptoms, therefore constituting a promising avenue for predicting symptom trajectories and potentially delivering just-in-time interventions.

## Results

**Fig. 1:**
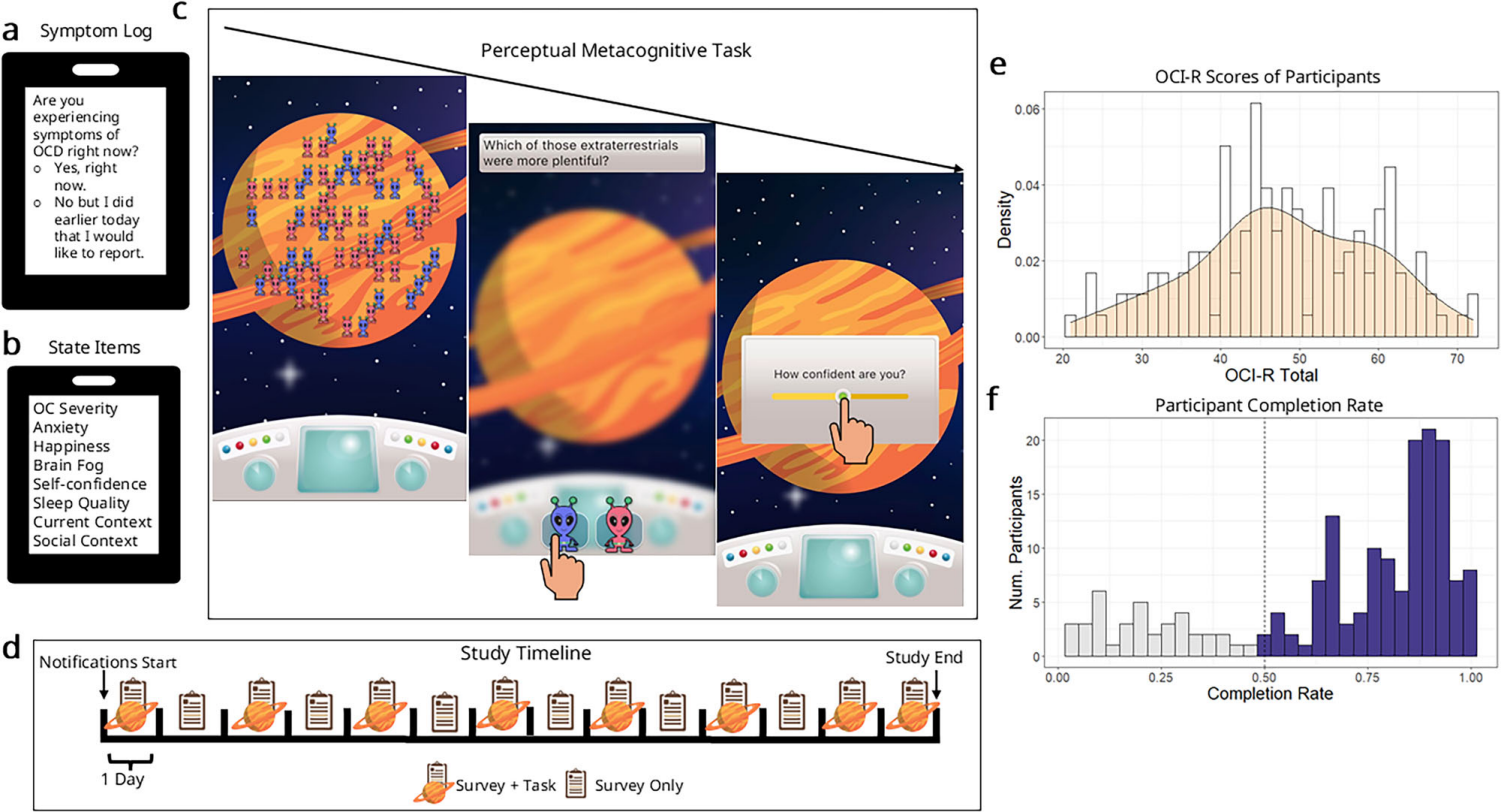
Microlongitudinal study design to determine the link between real-world fluctuating symptoms of OCD, self-confidence and metacognition during decision making. **a** Participants were asked to log in the app whenever they experienced episodes of OCD. They could press ‘Yes, right now’ if current experiencing symptoms, or otherwise ‘No’ to retrospectively log symptom that happened earlier that day. **b** Participants answered a survey probing states four times a day (with a minimum of 2 h between notifications) that probed the listed constructs. **c** On alternate days (eight total), participants completed a perceptual metacognitive task at the same time as one of the four state notifications. Trials begin with participants viewing a total of 64 aliens, mixed of two different colours on a planet for 250 ms. They are asked to select which alien is more abundant on the planet and then rate their confidence in their decision on a sliding bar. The task lasted 80 trials each play (40 trials per block) ~roughly 5 min per play. **d** Study timeline depicting the ecological momentary cognitive testing (EMCT) design over 14 days. **e** All participants scored highly on an obsessive-compulsive scale (OCI-R, ≥21 being the traditional cut-off for OCD). **f** Participants’ data were used in the analysis if they completed at least 50% of all notifications. We excluded 38/175 people for not meeting this criterion. Note that artwork for (**c**, **d**) are adapted from the Brain Explorer app: https://brainexplorer.net/ created by senior author Tobias Hauser.

We conducted a microlongitudinal study in 137 participants investigating how fluctuations in metacognition were driving day-to-day OCD symptoms. Participants (76.6% female, 21.2% male, 2.2% non-binary; age: mean [*M*] = 32.8 years ± 8.22 [standard deviation]) were recruited via an online worker platform and were included based on a self-declared diagnosis of OCD and a score of ≥21 (the traditional cut-off for clinically significant OCD) on the obsessive-compulsive inventory-revised (OCI-R^17^; *M* = 48.04 ± 11.43—Fig. 1).

After an initial baseline assessment, participants followed a 14-day procedure in which they were instructed to log any symptoms of OCD (defined as any instances of intrusive thoughts or compulsive behaviour) by tapping on a button on the home screen of the study app (see Fig. 1a). This self-initiated ‘symptom logging’ was used to capture instances of OCD episodes independent of scheduled notification periods (see below). This has been successfully adopted in prior work to assess emotional states following specific events, for instance, after non-suicidal self-injury^18,19^, but, until now, not in OCD.

In total, 119 participants logged a total of 1862 symptoms (median logs = 6, interquartile range = 15.5) throughout the study period. Alongside symptom logging, participants were repeatedly notified to report on their subjective states (four times a day), including subjective self-confidence (Fig. 1b), and play a perceptual metacognitive task (once every other day) (Fig. 1c, d). The combination of symptom logs with state notification reports provided enhanced insight into the drivers of OCD symptoms, allowing us to measure instances of symptom logs close in time to state confidence. Eighteen participants did not log any symptoms throughout the testing period, although they did complete the state-notified assessments.

Participants reported a total of 6389 states across the 14 days (mean notification completion rate [proportion]: *M* = 0.82 ± 0.13; mean total number of notifications completed per person: *M* = 46.6 ± 7.79) and completed a total of 922 cognitive games (*M* = 7.4 ± 2.7). See Fig. 1f and 'Methods' for details of data and participant exclusion criteria.

To validate our symptom logging approach, we also assessed OCD severity during state notifications by asking participants to rate their symptoms, as is standard in other momentary sampling studies^20^. To better understand factors relating to and fluctuating with confidence and OCD symptoms, we also asked participants other questions related to mental health (e.g. current anxiety, mood and sleep quality—see 'Methods').

### Frequency of self-initiated OCD symptom logging is linked to other state measures and external contexts

**Fig. 2:**
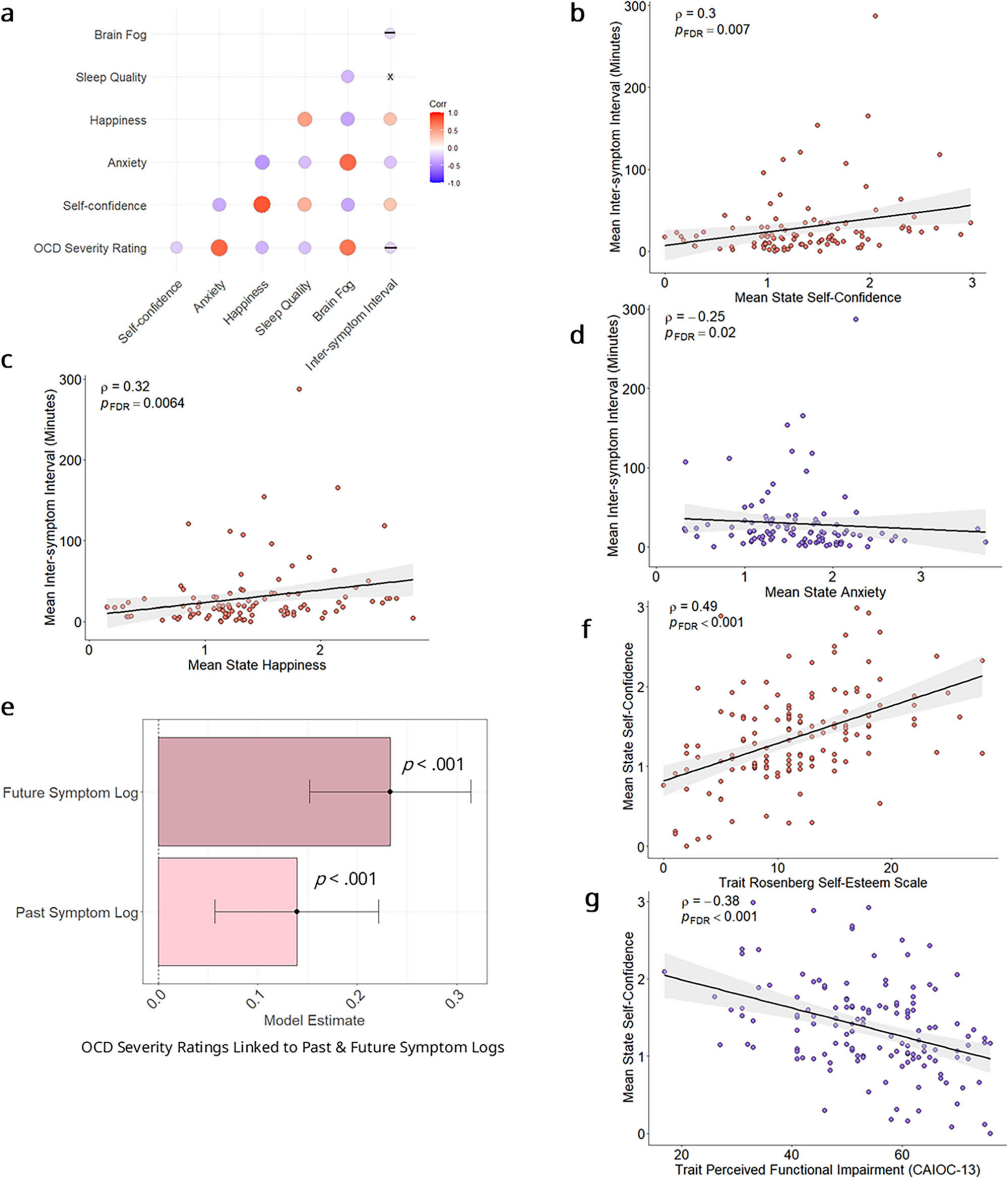
Identifying correlations between trait- and state-measures, with a focus on OCD symptoms and self-confidence. **a** Correlation matrix showing Spearman relationships between averaged state measures. ‘X’ indicates correlation is not statistically significant, while a ‘-’ indicates marginal significance (*p*-FDR <0.1). **b**–**d** Scatterplots depicting key relationships between averaged states and symptom log frequency (quantified as mean time difference between logs). State measures are always displayed on the x-axis. Spearman’s Rho (*ρ*) is displayed in the top left of each plot. Higher averaged time between symptom logs (inter-symptom interval) was significantly correlated with averaged self-confidence (**b**) and happiness (**c**), but inversely related to averaged anxiety (**d**). **e** Results from a mixed-effects model with random intercepts showing OCD severity ratings during notifications are significantly linked to past and future OCD symptom logs within a 4-h window. *n* (sample size) = 119; error bars = 95% confidence intervals with centre representing fixed effect value (beta) from mixed-effects model. Scatterplots depicting correlations between averaged self-confidence and self-esteem (**f**) and between averaged self-confidence and perceived functional impairment related to OCD (**g**). Across all scatterplots, purple colours indicate significant negative relationships, while red indicates significant positive relationships. Shaded bands represent 95% confidence intervals. Spearman correlations were used for all correlational analyses, which were corrected for multiple comparisons using the false discovery rate. All tests used were two-tailed.

First, as a validity check, we investigated whether self-initiated OCD symptom log frequency was associated with well-established measures (e.g. OCD severity and mood) (Fig. 2a). We quantified symptom logging frequency as the mean time difference between symptom logs (inter-symptom interval) in hours per participant (the lower the time, the greater the density). We implemented this instead of simple counts of symptom logs, which is more susceptible to compliance to the study protocol, while averaged time-between-logs better reflect intensity of symptoms (i.e. greater intensity when logs are closer in time)^21^. We found that people who had a smaller inter-symptom interval also overall reported increased state anxiety (*rho* = − 0.25, *p*-FDR [false discovery rate corrected] = 0.020; Fig. 2d) and an increase in averaged state OCD severity ratings (*rho* = − 0.19, *p*-FDR = 0.077, *p*-uncorrected = 0.050), albeit the latter association did not reach corrected statistical significance. Conversely, the inter-symptom interval was linked to a reduced averaged state confidence (*rho* = 0.30, *p*-FDR = 0.007; Fig. 2b) and happiness (*rho* = 0.32, *p*-FDR = 0.0064; Fig. 2c). These relationships align with findings from prior trait work reporting that OCD symptomatology is tied to constructs of mood, anxiety and confidence^12,22,23^ and thus provides evidence that our OCD symptom log data captured meaningful variation in behaviour.

Next, to further assess symptom log validity, we confirmed that symptom logs were significantly associated with moment-to-moment state OCD severity ratings (see Supplementary Note 3 for detailed analyses on OCD severity ratings and other state and trait scores) during notification periods. Indeed, current OCD severity ratings were positively associated with past (*β* = 0.149, 95% CI [0.06 0.22], *p* < 0.001) and future symptom logs (*β* = 0.234, 95% CI [0.15 0.31], *p* < 0.001) within a 4 h-window (Fig. 2e), indicating that our symptom log measure is temporally linked to (more traditionally implemented) ecological momentary assessment (EMA)-based OCD severity ratings^20^.

Additionally, we confirmed that symptom logs were meaningfully capturing external events (i.e. whether the probability of a symptom log varied based on current activities and social contexts) and explored how symptom log occurrence fluctuated by the time-of-day and the day-of-week. Briefly, we uncovered that OCD symptom logging was, on average, more frequent on weekdays (per day mean = 2.34 ± 3.73) compared to weekends (per day mean = 1.95 ± 3.38), Wilcoxon’s *V* = 4506, *p* = 0.008, and that partaking in restful (odds ratio = 0.59, *β* = − 0.54, *p* < 0.001), and self-care (odds ratio = 0.69, *β* = − 0.37, *p* = 0.008) activities promoted lowered likelihood of symptom logging. This suggests, intuitively, engaging in relaxing or enjoyable activities is associated with reduced OCD symptoms (see Supplementary Note 2 for further analyses of these contextual and environmental effects on symptoms).

Our findings thus support the ecological validity of this logging approach, allowing us to further investigate the relationship between symptom logs and its drivers.

### Self-confidence linked to self-esteem and perceived cognitive impairment

Because our momentary assessment of self-confidence (probed using the question ‘How confident do you feel right now?’—see 'Methods') was not used in this context before, we assessed whether it is associated with well-established trait measures. Indeed, participants with greater trait self-esteem (measured with the Rosenberg self-esteem scale^24^) also reported increased mean state self-confidence (*rho* = 0.49, *p*-FDR < 0.001; Fig. 2f), thus confirming the construct validity of our state self-confidence question. Additionally, more (averaged state) self-confident participants reported lower perceived cognitive impairment related to OCD (*rho* = − 0.38, *p*-FDR < 0.001, Fig. 2g; measured using the Cognitive Assessment Instrument of Obsessions and Compulsions-13^25^; example item: 'Do you doubt having done things properly?'), suggesting that self-reported confidence is also linked to OCD-related metacognition.

These trait-state relationships were maintained even when accounting for other state measures (mean anxiety, happiness, sleep quality and brain fog) using partial Spearman correlations (see Supplementary Note 1). Further analyses on links between self-confidence and external contexts, the time of day and the day-of-week can be found under Supplementary Note 2.

### Reduced self-confidence predicts future OCD symptom logs

**Fig. 3:**
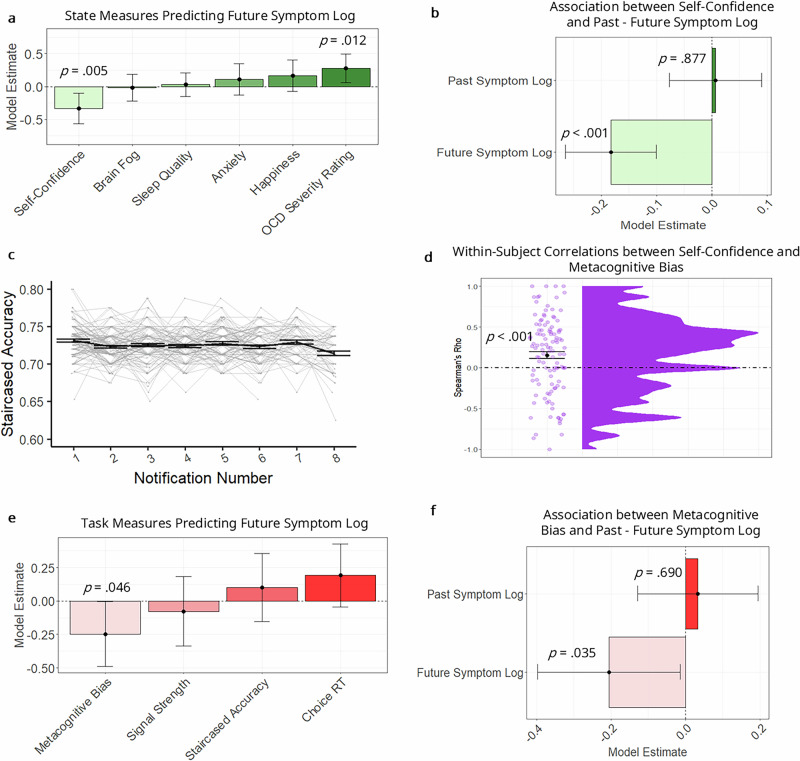
Characterising associations between self-confidence, metacognitive bias and OCD symptom logs. **a** Low self-confidence and high OCD severity significantly predicted the occurrence of a future OCD symptom log. **b** The effects of confidence on symptom logs are unidirectional; high self-confidence is associated with a lower probability of a symptom being logged, but occurrence of a past symptom log was not associated with future self-confidence. Past and future symptom logs (within 4 h of answering the state questions) were used as independent variables in a logistic mixed-effects model predicting self-confidence. **c** Our staircasing procedure succeeded in producing relatively stable accuracy (~0.72) across participants and notifications. The plot depicts mean staircased accuracy (grey) and group mean ± standard error of the mean (black) across notifications (x-axis). Each grey line is an individual participant. **d** Raincloud plot showing an overall positive within-participant correlation between metacognitive bias and self-confidence (significantly different from 0 using the Wilcoxon one-sample signed rank test). Each circle represents one participant’s Spearman’s Rho value quantifying the correlation strength between their own self-confidence and metacognitive bias. Overall mean correlation coefficient and standard error are shown in black. **e** Low metacognitive bias significantly predicts future symptom logs above other task measures, and **d** the effect of metacognitive bias over symptom logs is unidirectional; metacognitive bias significantly predicts future symptom logs, but past symptom logs do not predict metacognitive bias. Error bars for **a**, **b**, **e**, **f** = 95% confidence intervals with centre representing fixed effect value (beta) from mixed-effects models; *n* for **a**, **b**, **e**, **f** = 119; *n* for **c**, **d** = 137. All bar plots depict model coefficient estimates from mixed-effects models with random intercepts. All tests used were two-tailed.

Our main question in this study was to assess whether and how fluctuations in momentary confidence contributed to the emergence of OCD symptoms. We therefore leveraged our temporally fine-grained assessments to not only investigate associations, but also the directionality of these effects. To do this, we used logistic mixed effects models, with state measures predicting whether a symptom was logged within 4 h of completing a state questionnaire. We found that above other state measures (anxiety, brain fog, happiness and sleep quality), a future symptom log was best predicted by reduced self-confidence (odds ratio = 0.72, *β* = − 0.33, *p* = 0.005), as well as increased OCD severity ratings (odds ratio = 1.32, *β* = 0.28, *p* = 0.012)—Fig. 3a. These significant effects were maintained even when subsequently controlling for activities people reported they were currently doing. The association with OCD severity ratings supports the notion that symptom logging in this study reflects genuine OCD ratings, while the association with self-confidence expands current understanding of how confidence contributes to OCD symptoms. These results held even when testing shorter time windows between symptom logs and state questionnaires (i.e. within 3 h: *β* = − 0.23, *p* < 0.001; 2 h: *β* = − 0.23, *p* < 0.001; and 1 h: *β* = − 0.29, *p* < 0.001). This means that drops in self-confidence were meaningfully associated with OCD symptoms.

Next, we were interested in the directionality of these effects, i.e. whether drops in self-confidence preceded increased OCD severity or vice versa. To this end, we tested whether self-confidence was more related to past (OCD_t − 1_; suggesting OCD affects future confidence) or future OCD logs (OCD_t + 1_; confidence affecting OCD). We uncovered that the relationship between self-confidence and the likelihood of logging a symptom was unidirectional: in a linear mixed-effects model, current self-confidence was significantly associated with a future symptom log (*β* = − 0.18, 95% CI [−0.26 −0.10], *p* < 0.001; Fig. 3b) but not with a past symptom log (*β* = 0.007, 95% CI [−0.08 0.09], *p* = 0.877). These findings were maintained even when using past, current and future OCD severity ratings during timed notifications to predict self-confidence—see Supplementary Note 3. This means that drops in self-confidence temporally precede OCD symptoms, but OCD symptoms do not precede drops in self-confidence.

### Task-derived metacognitive bias is coupled with self-confidence

Next, we were interested in whether metacognition influencing symptom occurrence was specific to self-reported self-confidence, or whether it generalised to other metacognitive measures, such as task-related confidence. To this end, we asked participants to play a smartphone-compatible gamified perceptual metacognition task (eight times total throughout the EMCT period; Fig. 1b) with staircased performance^26^, an essential component for computational metacognition research. We ascertained that the staircasing procedure succeeded in producing an averaged (proportion) accuracy of 0.72 ± 0.005 across all participants and across eight sessions (Fig. 3c), within the range of average staircased accuracy reported in prior metacognitive studies^14,27–29^. Each individual session also yielded averaged accuracies ranging from 0.71 to 0.73. Moreover, in a linear mixed-effects model with session number predicting staircased accuracy, we found that accuracy did not significantly fluctuate by session (*β* = 0.023, 95% CI [−0.055 0.009], *p* = 0.164), indicating that practice effects did not substantially impact participant accuracy.

To further study the construct validity of the metacognitive measures, we investigated whether the self-reported self-confidence was linked to task metacognition. From the metacognition task, we derived two commonly studied measures: (i) metacognitive bias (operationalised as mean confidence rating^30^), where low bias indicates underconfidence while high bias indicates overconfidence and (ii) metacognitive efficiency (meta-d’/d’), derived using signal-detection theoretic computational models^31^, which quantifies whether task confidence ratings are sensitive to correct and error trials while controlling for performance (d’). Here, *metacognitive bias* was defined according to metacognition research conventions^9,27,29,30,32,33^, where it refers to the overall expressed confidence under staircased accuracy conditions—not to the calibration bias in a strict sense of a deviation from a normative reference point.

We utilised these task measures based on existing literature, in which some reports indicate that obsessive-compulsive symptoms are associated with biased (e.g. too low) reporting of confidence (i.e. related to metacognitive biases) (see Hoven et al.^12^ for review) while other findings suggest compulsivity is linked to imprecise confidence judgements^13,34^, manifesting as decreased sensitivity in delineating correct from incorrect choices in their confidence ratings (i.e. pertaining to metacognitive efficiency)^27,35^.

We found a significant within-participant correlation between metacognitive bias and self-confidence (Fig. 3d; median Spearman’s rho = 0.24, one-sample Wilcoxon rank-sum test: *p* < 0.001, % participants showing a positive correlation coefficient ≥0.1 = 57%, participants showing a negative correlation coefficient ≤ − 0.1 = 29%), meaning that task-based metacognitive bias co-fluctuated with self-reported self-confidence over time. Metacognitive efficiency was not significantly associated with self-confidence (one-sample Wilcoxon rank-sum test: *p* = 0.267). We formally tested the robustness of the association between self-confidence and metacognitive bias in a model controlling for other state measures (anxiety, brain fog, happiness and OCD severity rating). Indeed, only self-confidence was significantly associated with metacognitive bias (*β* = 0.12, 95% CI [0.04, 0.20], *p* = 0.002). The relationship between metacognitive bias and self-confidence was maintained (*β* = 0.12, 95% CI [0.05 0.19], *p* < 0.001) even when controlling for other task measures, namely signal strength (numerical difference between task stimuli; a measure of task difficulty), choice reaction time and staircased (i.e. adaptive; see 'Methods') accuracy. These findings provide strong evidence for different confidence measures measuring a common underlying metacognitive construct, even though the assessment modalities (self-report vs task) and frequency (four times daily vs every other day) differed substantially.

### Metacognitive bias fluctuations drive future OCD symptom logs

Here, we assessed whether the task-based metacognitive bias was linked to OCD symptom logs. When inserted into a logistic mixed effects model, lower metacognitive bias (i.e. underconfidence; odds ratio = 0.75, *β* = − 0.28, *p* = 0.022), but not metacognitive efficiency (*β* = 0.027, *p* = 0.839), was significantly associated with a future symptom log (within a 4-h period from completing a game).

We conducted further analyses to thoroughly assess the robustness of the relationship between metacognitive bias and symptom logs. First, controlling for other task measures in the same model still indicated that reduced metacognitive bias was significantly associated with a future symptom log (odds ratio =  0.78, *β* = − 0.25, *p* = 0.046)—see Fig. 3e. Next, the relationship was maintained (odds ratio = 0.60, *β* = − 0.51, *p* = 0.036) when controlling for state measures (anxiety, brain fog, happiness, OCD severity rating and sleep quality) that were close in time to the completion of the task (within at most a 4-h period). In addition, using shorter time windows between game completion and symptom log (<4 h) yielded similarly significant results (3 h: *β* = − 0.26, *p* = 0.046; 2 h: *β* = − 0.36, *p* = 0.011; 1 h: *β* = − 0.31, *p* = 0.048).

We also investigated the temporal succession of these effects, i.e. whether a drop in metacognition preceded or followed the emergence of OCD symptoms. In line with our self-confidence findings, we saw that reduced metacognitive bias was linked to future symptom logs (*β* = − 0.21, 95% CI [−0.40 −0.015], *p* = 0.035), but past symptom logs did not impact current metacognitive bias (*β* = 0.03, 95% CI [−0.13 0.19], *p* = 0.690)— Fig. 3f.

Finally, to address the possibility that the observed effects reflect a systematic miscalibration rather than an overall confidence level, we reanalysed the data using the signed difference between confidence and accuracy (confidence − accuracy). This alternative operationalisation is conceptually stricter as it uses accuracy as an explicit reference point against which confidence is evaluated. The results held: this calibration-based bias measure similarly showed a negative association with future symptom logs (odds ratio = 0.75, *β* = − 0.28, *p* = 0.022), even when controlling for choice reaction times and signal strength (odds ratio = 0.78, *β* = − 0.25, *p* = 0.046). This indicates that participants who are underconfident relative to their actual performance are more likely to subsequently report OCD symptoms.

In summary, both task-based (as well as self-reported) metacognition preceded the appearance of OCD symptom logs. This indicates that fluctuations in metacognition predict a possible rise in OCD symptoms within a few hours’ time.

### Sensitivity analyses

In addition to robustness tests described above, we conducted several sensitivity analyses targeting potential imbalances and missingness in the data and found that the main findings were largely robust across these checks.

**Fig. 4:**
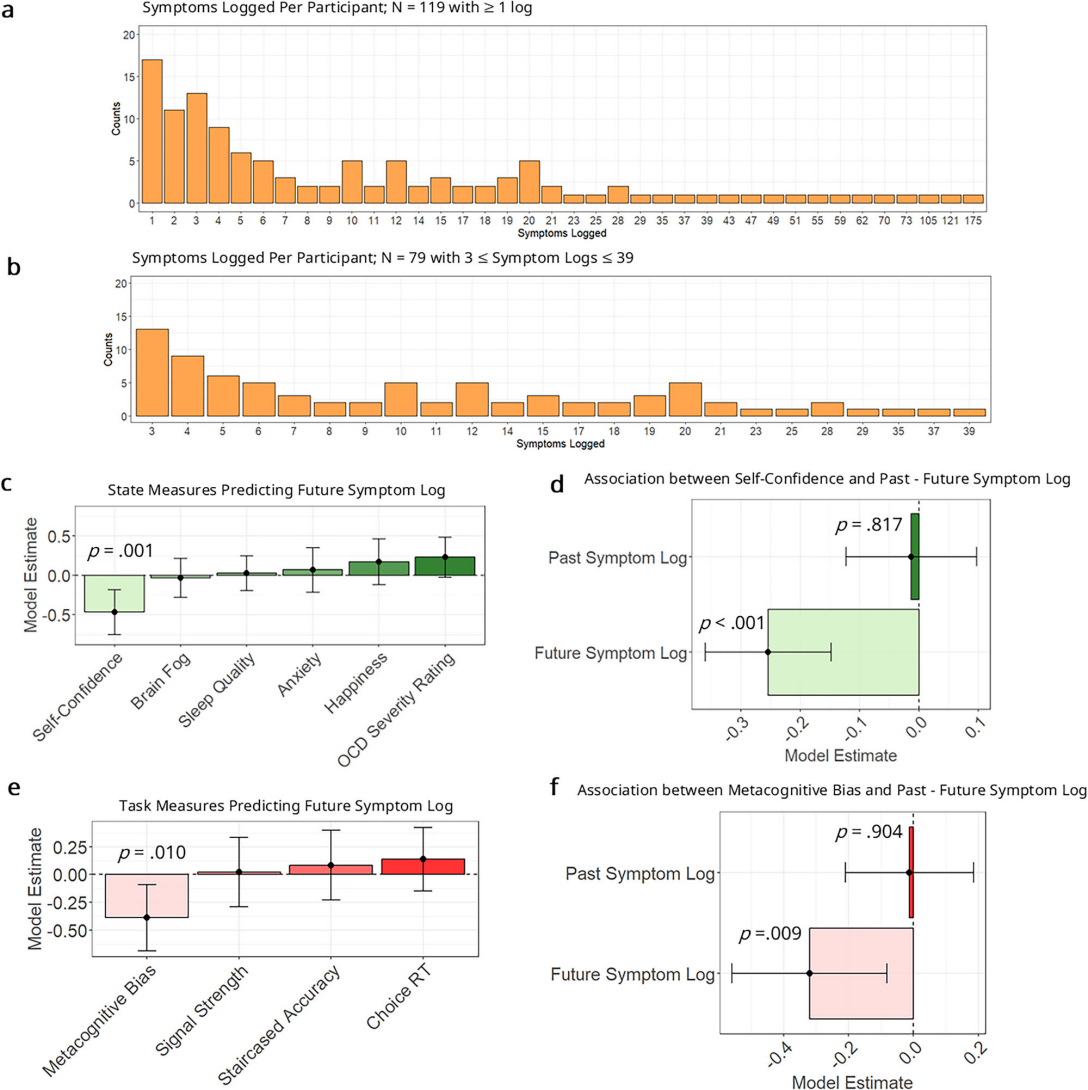
Sensitivity analysis to address the skewed symptom logging distribution. **a** Histogram of the number of symptoms logged per participant throughout the EMCT period. **b** Histogram of the number of symptoms logged per participant after removal of highly infrequent and frequent loggers. **c** Main results were maintained for self-confidence predicting future symptom logs, controlling for other state variables. **d** Self-confidence predicted a future symptom log, but past symptom logging was not significantly related to self-confidence. **e** Metacognitive bias still significantly predicted future symptom logging, controlling for other task variables. **f** Metacognitive bias predicted a future symptom log, but past symptom logging was not significantly related to metacognitive bias. Results **c**–**f** were obtained from the sample where overly frequent and infrequent symptom loggers were removed. Error bars = 95% confidence intervals with centre representing fixed effect value (beta) from mixed-effects models; *n* for **c**–**f** = 79. Bar plots depict model coefficient estimates from mixed-effects models with random intercepts. All tests used were two-tailed.

First, inspecting the distribution of symptom logs across the study period (see Fig. 4a) revealed that the data were highly skewed, with several participants only logging once or twice and a few showing extremely high logging frequency (e.g. 175 total logs). To assess whether our results were disproportionately influenced by these participants, we removed 'outlier' values following standard statistical conventions, i.e. participants showing symptom log numbers either above the 75% or below the 25% quartiles by a factor of 1.5 times the interquartile range. This initially led to the removal of 12 participants who showed extremely high logs, although no participants in the lower ranges met the cut-off for removal (using the quartile method, the cut-off for high logging was 41.75, while the cut-off for low logging was −20.25). Nonetheless, we removed those who logged only once or twice (*n* = 28) as we assumed this may be too infrequent to be meaningful. This led to a total of 79 participants retained with a number of symptom logs ranging between 3 and 39 (see Fig. 4b for distribution).

When removing the overly high- and low-frequency loggers, we found that self-confidence (odds ratio = 0.63, *β* = − 0.46, *p* = 0.001) still significantly predicted future symptom logging—Fig. 4b. Furthermore, a future symptom log, but not past logging, was still significantly associated with current self-confidence (future log: *β* = − 0.25, 95% CI [−0.36 −0.15], *p* < 0.001; past log: *β* = − 0.01, 95% CI [−0.12 0.10], *p* = 0.817; Fig. 4d). Additionally, metacognitive bias, but not other task measures, still significantly predicted a future symptom log (odds ratio = 0.68, *β* = − 0.39, *p* = 0.010; Fig. 4e). The relationship between metacognitive bias and a future symptom log was similarly intact (future log: *β* = − 0.32, 95% CI [−0.56 −0.08], *p* = 0.009; past log: *β* = − 0.01, 95% CI [−0.21 0.18], *p* = 0.904; Fig. 4f).

Next, our decision to retain participants who completed at least 50% of notifications may be viewed as too liberal, and hence we reattempted the analyses above (removing high and low frequency loggers) using a more stringent 75% cut-off (*n* = 101 before removing high/low frequency loggers, *n* = 56 after removal). We found that lowered state self-confidence (controlling for other state measures; *β* = − 0.41, odds ratio =  0.66, *p* = 0.010) and metacognitive bias (controlling for other task measures; *β* = − 0.46, odds ratio = 0.63, *p* = 0.005) still significantly predicted a future symptom log.

**Fig. 5:**
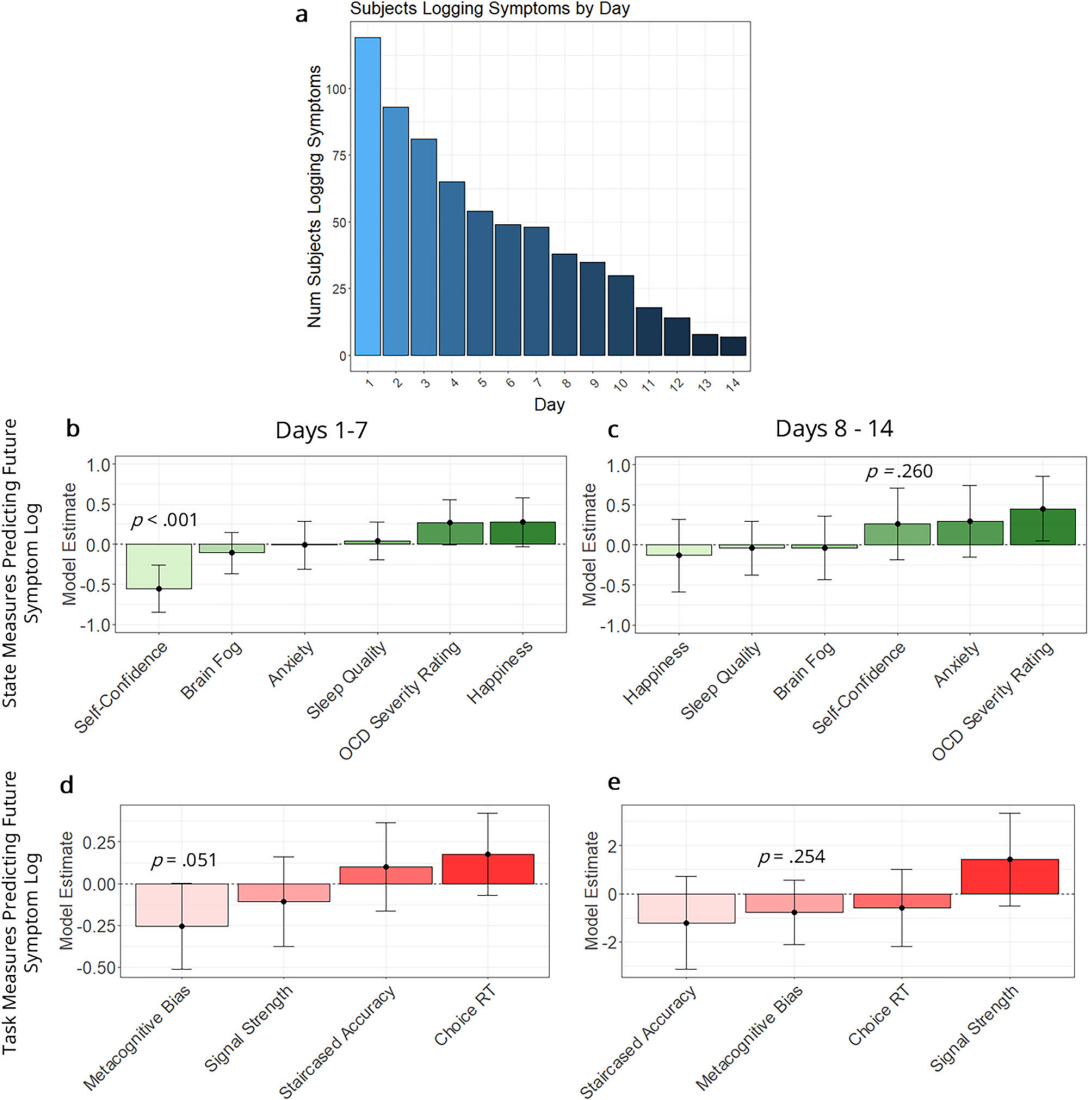
Sensitivity analysis comparing results from week 1 (days 1–7) and week 2 (days 8–14). **a** Number of participants logging symptoms decreased as the study continued. **b** During week 1, state self-confidence still significantly predicted a future symptom log. **c** When analysing only data within week 2, self-confidence was no longer significantly associated with symptom logs. **d** Within week 1, metacognitive bias was still inversely associated with future symptom logs, but the effect was no longer as strong as it was across all days. **e** When considering only week 2, the effect of metacognitive bias on symptom logs was no longer apparent. Error bars = 95% confidence intervals with centre representing fixed effect value (beta) from mixed-effects models; *n* for **b**, **d** = 115; *n* for **c**, **e** = 114. Bar plots depict model coefficient estimates from mixed-effects models with random intercepts. All tests used were two-tailed.

Lastly, we found that the number of participants logging symptoms depleted over the 14-day testing period—with 119 participants logging symptoms on day 1, but by day 8, this had diminished to 38 loggers, with day 14 only having 7 loggers left (Fig. 5a). We assessed whether this drop in symptom logging impacted our main results.

In the first week of the study (days 1–7), controlling for other state measures, state self-confidence was still significantly predictive of a future symptom log (odds ratio = 0.58, *β* = − 0.55, *p* < 0.001, Fig. 5b), while the effect was in the same direction for metacognitive bias (controlling for other task measures), albeit weaker (odds ratio = 0.77, *β* = − 0.26, *p* = 0.051, Fig. 5c).

However, both effects had disappeared by week 2/days 8–14 (state self-confidence: odds ratio = 1.29, *β* = 0.26, *p* = 0.260, Fig. 5d; metacognitive bias: odds ratio = 0.46, *β* = − 0.77, *p* = 0.254, Fig. 5e), potentially due to the limited availability of symptom log data. Thus, our conclusions hold under sufficient data density, and future work with higher compliance rates will be better positioned to determine whether this reflects a power limitation or if the effect may genuinely attenuate over time.

## Discussion

In this densely sampled longitudinal study, we established that fluctuations in metacognition potentially drive symptoms of OCD. During instances of reduced confidence, both in the self and in perceptual decisions, participants with self-declared OCD demonstrated a greater likelihood of logging symptoms. These effects were directional—lowered confidence preceded symptom logging, but past symptom logs were not predictive of future low confidence. Our findings thus support the notion that fluctuations in cognitive processes may predict (in a temporal sense) future mental health symptoms, and that in OCD, metacognition appears as a key driver.

Our EMCT study implemented naturalistic self-initiated symptom logging throughout the assessment period, which enabled us to effectively ascertain the temporal relationship between multimodal metacognitive processes and OCD symptoms. Our work contributes to the growing cognitive OCD literature, which has produced heterogeneous accounts regarding metacognition. To illustrate, clinical studies point to OCD being associated with reduced metacognitive bias, i.e. underconfidence, across multiple cognitive domains^12^ while transdiagnostic research indicates that, in general populations, compulsivity is more associated with inflated confidence^11,14,27,36^ and possibly metacognitive inefficiency^27,35^. However, cross-sectional studies, intrinsically, are unable to truly capture variability in these erratic processes. Thus, our EMCT design advances existing knowledge by providing (i) insight into how these processes interact over time while controlling for potential confounding factors, and (ii) assessment of the temporal directionality between processes. Using this state fluctuation approach, we established that it is reductions in confidence that precede OCD symptoms within the timescale of a few hours. Moreover, our online sample, who have self-identified as having OCD and show high obsessive-compulsive scores, may reveal metacognitive patterns (i.e. lowered bias) more akin to in-person clinical samples than to online transdiagnostic samples^36^, signifying strong clinical relevance. It is now crucial to understand whether clinical and subclinical/transdiagnostic populations are in alignment on these within-subject momentary associations between metacognition and OCD(-like) symptoms, and this may shed light on diverging cross-sectional findings thus far.

Leveraging EMCT, our findings suggest metacognitive processes from different modalities (i.e. task vs self-report) may be interrelated and shape mental health. We uncovered that confidence during perceptual decision-making was significantly related to one’s own self-confidence on a moment-to-moment basis. This points to metacognition portraying aspects that generalise across domains and assessment modalities, rather than being context specific. Moreover, our results are in line with a notion that metacognitive processes are organised hierarchically, an ongoing and pertinent area of study^37^ (see Seow et al. for a recent conceptual review^16^). Briefly, lower levels along the hierarchy are thought to pertain to ‘local’ metacognition (i.e. confidence in specific contexts, such as trial-by-trial decisions on our cognitive task) and higher levels represent ‘global’ metacognition related to metrics like confidence in oneself (i.e. what our self-confidence item taps into). Indeed, compulsivity has been linked to specific alterations at different levels of the metacognitive hierarchy^14,38^. In the context of the present study, we demonstrate how self-beliefs perhaps generalise across global and local metacognitive levels and that alterations in these beliefs go on to potentially drive obsessive-compulsive symptoms. Nonetheless, we note some caveats to this speculation. First, our work has stopped short of addressing person-level heterogeneity in the relatedness between levels of metacognition: our findings uncovered a proportion of the sample (29%) displayed a negative relationship (correlation coefficient of ≤ − 0.1) between metacognitive bias and self-confidence. Future work should address whether this dissociation is meaningfully related to psychopathology and has implications for one’s broader metacognitive processes. Next, while averaged moment-to-moment self-confidence was significantly correlated with trait-level self-esteem, research needs to investigate whether this confidence measure truly is representative of one’s global unified self-image. For instance, assessing whether long-term changes in self-esteem reflect shifts in momentary self-confidence. Lastly, we acknowledge that the tested ratings (decision-making and self-confidence) remain limited in scope, and future work should characterise how more diverse domains of confidence are interrelated, for instance, along the lines of previous research that has compared memory and perceptual confidence^14^.

As our findings indicate that task metacognitive biases drive moment-to-moment symptom emergence, this could serve as a foundation for future work to incorporate mobile neuroimaging techniques with EMCT to identify the neural mechanisms underlying these fluctuations. Whilst the neural mechanisms associated with fluctuations in metacognition remain unknown, we speculate that connectivity between prefrontal cortices and right dorsal anterior cingulate cortex and striatal regions could act as drivers given their involvement both in metacognition^39,40^ and OCD symptomatology^7,41^. Nonetheless, if we just wanted to predict the likelihood of future symptom occurrence, a simple confidence self-report measure could suffice over a more time-consuming task-confidence paradigm^42^.

Ultimately, this study paves the way for the design of just-in-time interventions targeting reduced confidence to alleviate symptoms of OCD as they occur. Prior research has already established that cognitive behavioural therapy targeting meta-memory leads to immediate improvements in memory confidence, and corresponds to decreased checking symptoms over time^43^, while deep brain stimulation applied to the ventral striatum leads to an immediate increase in self-confidence, coupled with improved OCD symptoms^44^. These studies collectively point to confidence as an effective intervention target, while our current EMCT study unveils the promise of mobile confidence and symptom tracking to define precisely when interventions should be delivered (i.e. during drops in confidence) to maximise effectiveness.

While this line of research is still in its infancy, we speculate that task-based metacognitive interventions may prove effective, for instance, participants could be provided easy practice trials with a greater frequency of positive feedback, as this has been found to improve both trial-by-trial and overall task confidence in prior work^29^. Just-in-time interventions could also build on existing metacognitive training programmes, shown to be effective for disorders like OCD and depression^43,45^. These trainings could be delivered by clinicians, followed by reminders/summaries of training material sent to participants’ smartphones, triggered by reports of low confidence and symptom logging. Moreover, therapist access to these momentary assessment reports could improve therapeutic treatment outcomes, as they can provide rich context^46^ into where and when low confidence and OCD symptoms could occur.

Lastly, it is important to highlight that we did not collect information on current medication intake and thus are unable to assess how OCD symptom fluctuations may have been affected by momentary psychiatric drug use. Next, as is typical in ecological momentary assessment (EMA) research, our dataset, particularly the symptom logs, exhibited unevenness and missingness, with some participants completing entries infrequently or insufficiently. Given the high notification frequency (four times per day), we adopted a 50% completion threshold, which could be considered relatively liberal compared to other EMA studies^47^. Our primary findings remained robust despite these limitations, suggesting the presence of a genuine effect. Nonetheless, we did demonstrate that our results may rely on having enough participants logging to demonstrate that reduced confidence precedes symptoms, as the effect was weakened in the 2nd week of the study when substantially less people were logging symptoms. Albeit, do note that our study design does not allow us to ascertain whether the lack of significance is due to insufficient power or a genuine absence of effect later in the study; it is uncertain whether repeated engagement with the app may have reduced the sensitivity of confidence ratings and symptom logs as measures, independent of compliance rate. All in all, it is important for future work to design techniques for improving compliance for self-directed reporting, which can simultaneously examine whether the ecological validity of self-report degrades over the course of such studies. One method could be to notify participants, at the end of the day, to explicitly record whether they did NOT experience any symptoms that day, and to otherwise retrospectively log them. This method additionally aids in distinguishing between a true lack of symptoms and non-compliance.

In sum, our study provides empirical evidence for metacognitive processes contributing to fluctuations in symptoms of OCD. We reveal that moment-to-moment reductions in confidence across different domains (in the self and in perceptual decision-making) are associated with a greater likelihood of reporting symptoms. This implies that lowered confidence can serve as a temporal marker for episodes of OCD, and further, underconfidence could be targeted with remote and/or mobile interventions to facilitate momentary alleviation of symptoms.

## Methods

### Participants

The study was conducted online (https://prolific.co/), following three distinct stages: pre-screening, baseline and EMCT. We pre-screened 290 participants (residing in the United Kingdom) who declared they had a diagnosis of OCD on Prolific. Other inclusion criteria at the pre-screening stage were as follows: aged 18–55 years, fluency in English, and smartphone ownership. During pre-screening, we selected participants who scored ≥21 on the OCI-R (the traditional cutoff for OCD^48^) and who did not have a history of neurological conditions or a diagnosis of Schizophrenia/psychosis. Following pre-screening, 286 participants were invited to the baseline assessment, which involved completing trait questionnaires and being briefed on the EMCT portion of the study; 201 participants completed this stage and were enrolled onto the EMCT part. After removal of dropouts and people who did not meet the completion criterion (minimum 50% EMCT notification completion), we analysed data from 137 participants (see Fig. Supplementary 1A for participants recruitment flowchart), 119 of which logged symptoms of OCD at least once throughout the study period. While our final sample size aligns with prior EMCT work^2^, no formal statistical method was used to predetermine sample size. The study was approved by the University College London Research Ethics Committee (reference: 23505/001), and all participants provided informed consent. See Fig. S2 for further demographic information.

### Design and procedure

During pre-screening, participants completed the OCI-R and declared any psychiatric and/or neurological diagnoses. During baseline, participants completed demographic and trait questionnaires on REDCap (Research Electronic Data Capture). Next, EMCT was completed on participants’ own smartphones within the Brain Explorer Android or iOS app (www.brainexplorer.net). EMCT began the following morning after enrolment onto the study using the app. Participants resumed their usual daily routines while responding to notifications prompting them to complete state reports and a perceptual metacognitive game over the next 14 days. This timeframe was chosen based on prior work demonstrating promising links between mood and computational task parameters over a 2-week period^2^. Participants received four state notifications total per day: one every morning (between 08:30 and 11:30) and three notifications between 13:30 and 21:30. Actual notification times were spaced at least 2 h apart from each other and were pseudorandomised. Participants knew to expect four notifications a day but were not informed of the exact delivery times to minimise expectation effects. We programmed notifications to be sent out this frequently (×4 a day) to rigorously capture time-of-day effects on symptom severity—as OCD symptoms are reported to fluctuate significantly within a single day^4^. The perceptual metacognitive task (see description below) was played on alternate days (8 games total). The number of games associated with state notifications in the morning, afternoon, evening and night was counterbalanced (twice for each time of day).

Participants were reimbursed at a base rate for completing the screening and trait questionnaires (£5.25) and received additional payment at the end of the 14-day study based on the number of assessments completed: they were informed they could earn up to £15 extra if they completed 70% of assessments, and up to £20 extra if they completed 100% of assessments. They were informed that they had to complete surveys/tasks within one hour of receiving the notification, otherwise the assessment would not count towards their completion rate. Participants who had missed more than two notifications in a row also received personalised reminders from the experimenter over the Prolific messaging platform to encourage compliance.

### Assessments

#### Trait and demographic questionnaires

Participants completed the Cognitive Assessment Instrument of Obsessions and Compulsions (CAIOC-13)^25^, Rosenberg Self-Esteem Scale^24^, Barratt Impulsivity Scale-11 (BIS-11)^49^, Intolerance of Uncertainty Scale (IUS)^50^, Patient Health Questionnaire-9 (PHQ-9)^51^ for depression severity, trait anxiety scale (STAI-T)^52^ and the obsessive-compulsive inventory-revised (OCI-R)^48^, as well as demographic questions (age, gender, ethnicity, education level and socioeconomic status).

#### Subjective states and momentary contexts

The state assessment included eight items total, seven of which occurred at every notification timepoint, while an additional sleep quality item was in the morning only (see Table S1 in Supplementary).

Previous momentary sampling studies of OCD have predominantly assessed symptoms using dimension-specific items, such as those targeting checking or cleanliness (see Braga et al., for extensive review)^53^. However, given the substantial heterogeneity in disorder presentation, we opted for a broader question probing symptom intensity based on an item successfully utilised in EMA work identifying candidate neural biomarkers (using intracranial electrophysiology) of OCD severity^20^. Past EMA research was also used to inform the self-confidence item^54^.

We tested for state anxiety^54,55^, and happiness/mood^54,56^ because OCD has high comorbidity rates with both anxiety and depressive disorders^22^, and it is thus important to control for these variables when investigating the link between OCD and confidence. Similarly, we assessed sleep quality once a day due to the disorder being associated with sleep disturbances and reduced sleep efficiency^57^.

To address the potential impact of poor concentration or diminished thought clarity resulting from OCD symptoms on performance in the perceptual metacognitive task, we included a ‘brain fog’ item designed to assess difficulties with mental clarity. Evaluating this aspect was considered important, as ‘mind-blanking’ is also observed during states of anxiety^58^.

To assess the external validity of our state measures, we also examined how states are influenced by current activities and social contexts. This approach was particularly relevant given that daily routines and situational factors have been shown to affect OCD symptom severity^59^.

The first 6 state questionnaire items were rated on a 5-point Likert scale (not at all, a little, moderately, very, extremely), while the current activity item contained check box options (‘Work/school/studying/training’, ‘resting/relaxing’, ‘Everyday tasks/errands/shopping’, ‘Inactive leisure activities (e.g. reading, surfing the net, social media, games and watching TV)’, ‘Active leisure activities (e.g. sports, hiking)’, ‘travelling (e.g. on foot, car and public transport)’, ‘Self-care/personal hygiene/eating/drinking’) and the social context item had the options ‘Alone’ and ‘With others’. Activity and social context options were obtained from existing items used in EMA research (German Center for Mental Health, 2024).

#### Symptom log

Participants were asked to log whenever they were experiencing symptoms of OCD. This self-initiated logging enabled assessment of close-in-time associations between state and metacognitive measures used. To encourage compliance, we ensured that symptom logging required minimal effort; participants merely needed to tap on a ‘Symptom Log’ button on the app front page, and they would then be asked, ‘Are you experiencing symptoms of OCD (e.g. intrusive thoughts, compulsions) right now?’ Participants would then select ‘Yes’ if they were experiencing symptoms at the given moment. They were also able to retrospectively log symptoms, an option we provided to account for their symptoms of OCD interfering with their ability to log symptoms at the specific moment. To do this, in response to the question above, they would select the option ‘No, but I did earlier today that I would like to report’. They would then be able to select an approximate time (in 30-min intervals) that they last experienced symptoms. These retrospective logs were then placed into the appropriate timeframe in the dataset and were analysed alongside real-time logs. To encourage compliance further, we sent daily reminders in the evenings to notify participants to log symptoms of OCD if any symptoms occurred on the day. These notifications were pseudorandomised between 18:00 and 21:00 daily. Users could choose an estimated time, in 30-min increments, for when they last noticed symptoms.

#### Perceptual metacognitive task^60^

The task was implemented on the Brain Explorer app on participants’ mobile phones. Participants were instructed to make a perceptual decision based on an array of two different coloured aliens presented for 250 milliseconds (ms). They then had to decide which of the two aliens was more abundant by selecting the specific alien on screen. This was followed by a confidence rating in which participants had to rate their decision certainty on a scale from ‘totally guessing’ to ‘totally certain’ [these labels were adopted from prior work^60^]. Participants were instructed to use the full length of the scale.

The first-time participants played the task, they completed 20 practice trials in which they only made the perceptual decision (no confidence rating) and were given feedback, where either a green tick (correct) or a red cross (incorrect) appeared on screen. The actual task consisted of 80 trials divided into two blocks of 40 trials each (with the option for a break in between blocks). Feedback was not provided in the actual task. A maximum of 68 aliens were displayed on screen on any given trial.

A staircase procedure was used throughout the training and task to match participants’ performance^26^. Equating performance across participants in this way was done to enable observation of biases in task confidence ratings (i.e. metacognitive bias)^30^. To this end, we used a 2-up-1-down staircase procedure converging at ~70% accuracy (in practice, staircased accuracy ranged from 0.71 to 0.73 across the EMCT period), which was initiated during the 20 practice trials to minimise burn-in period. The difference between the two-coloured aliens was initialised at 15 and then would change by +4 or −4 with every step of the staircase. We used a continuous staircase, meaning that the staircasing did not reset between task plays. See Fig. S3 and Table S2 for visualisation and summary of task metrics across the eight sessions.

### Statistical analyses

Analyses were conducted using RStudio using R version 4.3.3. A statistical significance threshold of *p* < 0.05 was adopted, and all tests reported here were two-tailed unless otherwise stated.

#### Data validity checks

We conducted data validity checks before running the analyses. First, there were several instances where participants bulk completed notifications in one sitting. To account for this, in the initial 175 recruited participants, we removed data points that were too close in time to each other within each participant (>1 h). We always retained the first data point within a given hour (e.g. if notification A was completed first, and notification B was completed 5 min later, we would retain data from notification A and discard B).

Next, we excluded participants (38 out of 175 recruited participants) who did not provide data ≥50% of the total timepoints. The final sample (*N* = 137) represented 78.3% of the recruited sample (see participant recruitment and exclusion flowchart in Supplementary). This exclusion criteria applied only to timed notifications (i.e. for surveys and tasks). We did not have prior expectations over how often participants would conduct self-initiated symptom logging, hence we did not impose an exclusion criterion based on symptom-log frequency for our main analyses. Nonetheless, we conducted sensitivity analyses on a subset of the sample that did not show extremely low and high logging frequency to ensure our findings were not driven by these few participants (see ‘Sensitivity Analysis’ sub-section in the Results).

#### Trait-state relationships

Spearman correlations were employed to correlate averaged trait scores with mean (averaged across the 14 days) state measures and between each mean state measure (see Fig. S4). Benjamini–Hochberg’s false discovery rate (FDR) was used to correct for resulting *p*-values. Additionally, we conducted partial correlations and linear regressions to assess the robustness of key trait-state correlations (e.g. partial correlation between mean state self-confidence and trait self-esteem, controlling for mean state happiness). We also observed how states are correlated within people using within-subject Spearman correlations. We computed a Spearman correlation coefficient (rho) per participant for each pair of states (e.g. OCD severity and self-confidence; see Tables S3–S6).

#### Effects of external contexts on OCD symptomatology

We assessed whether symptom logs, state self-confidence, and state OCD severity were meaningfully capturing external events (i.e. whether the probability of a symptom log varied based on current activities and social contexts) and explored how these variables fluctuated by the time-of-day and the day-of-week (see Supplementary Note 2 for the method/results and Figs. S5–S7).

#### Regression analyses

To formally deduce how self-confidence and metacognitive bias impact moment-to-moment OCD symptoms, we employed various regression analyses. All continuous state and task measures were within-person centred (z-scored) before being inserted into the regressions. Logistic mixed effect models with random intercepts were used when ‘symptom log’ (presence [1] or absence [0] within a 4-h time window from the completion of a state or task notification) was inserted as the dependent variable. We assessed that accounting for within-person dependence improved model fit (or otherwise showed no difference in performance) compared to non-mixed effect models by reporting the estimated variance and standard deviation of the random effects. We also ran likelihood ratio tests comparing models with and without the random intercept (see Supplementary Note 4 and Table S7).

When analysing the impact of self-confidence on future symptom logs, we controlled for other state variables (OCD severity, anxiety, happiness, brain fog and sleep quality, see Eq. 1 and Fig. 3a). Next, when investigating the effect of metacognitive bias over symptom logging, we controlled for potential confounding task variables including staircased accuracy, choice reaction time and signal strength (Eq. 2 and Fig. 4c). Additionally, we checked that the relationship between metacognitive bias and symptom logging was maintained when controlling for state variables recorded close in time (≤4 h) to task completion (OCD severity, anxiety, happiness, brain fog and sleep quality) using a model similar to Eq. 1 but with metacognitive bias inserted as an independent variable instead of self-confidence.

The method of binarizing the symptom log variable in our mixed models partially accounts for imbalanced data because several participants simply did not log frequently enough to operationalise symptom severity as the total number of symptom logs within a 4-h period from state notification. However, treating the symptom log as a binary variable led to us retaining only the first symptom log close to notification completion and discarding symptom logs that were recorded after. We assessed the potential severity of this by calculating the percentage of logs discarded per participant (logs discarded/total number of logs). We found that several participants had no logs discarded (*n* = 45; 38% of our sample), while the median number of logs discarded across all participants was 1 (interquartile range = 4)—see Fig. S8 for visualisation. Considering the relatively low proportion of data loss, we determined that logistic regression models were suitable for our analysis. However, we recognize that in samples with greater logging frequency, a linear model that retains all data would be preferable.

These logistic mixed-effect models were fitted using the glmer function from the R *lme4*^61^ package. To obtain accurate maximum likelihood estimates, we used adaptive Gauss–Hermite quadrature with 10 points (*nAGQ* = 10). The BOBYQA optimizer was specified via glmerControl(optimizer = “bobyqa”). The model was specified with a binomial (logit) link function.

1 $${{\rm{SymptomLog}}}_{i,t}\in \left\{0,\,1\right\} \sim {{\rm{SelfConfidence}}}_{i,t}+{\rm{OCDseverity}}+{{\rm{Anxiety}}}_{i,t}{+{\rm{Happiness}}}_{i,t}+{+{\rm{Brain}}\; {\rm{Fog}}}_{i,t}{+{\rm{Sleep}}\; {\rm{Quality}}}_{i,t}+\left(1|i\right)-$$

$$i={individual},t={time}\,{point}$$

2 $${{\rm{SymptomLog}}}_{{\rm{i}},{\rm{t}}}\in \left\{0,\,1\right\} \sim {{\rm{Metacognitive\; Bias}}}_{{\rm{i}},{\rm{t}}}+{\rm{Staircased}}{{\rm{Accuracy}}}_{{\rm{i}},{\rm{t}}}{+{\rm{Choice\; RT}}}_{{\rm{i}},{\rm{t}}}+{+{\rm{Signal\; Strength}}}_{{\rm{i}},{\rm{t}}}+\left(1|{\rm{i}}\right)-$$

$$i={individual},t={time}\,{point},{RT}={Reaction}\,{Time}$$

For cross-correlation analyses (i.e. to check how OCD severity, self-confidence and metacognitive bias affects past and future OCD symptoms), we created lagged and leading versions of the symptom log variables. Lagged and leading symptom log variables were inserted in the same logistic mixed effect model predicting (1) OCD severity (Eq. 3 and Fig. 2e) (2) self-confidence (Eq. 4 and Fig. 3a) and (3) metacognitive bias (Eq. 5 and Fig. 4b).

3 $${{\rm{OCD\; Severity}}}_{i,t} \sim {{\rm{SymptomLog}}}_{i,t+1}\in \{0,1\}+{{\rm{SymptomLog}}}_{i,t-1}\in \{0,1\}+\left(1|i\right)-$$

$$i={individual},\,t={time}\,{point}$$

4 $${{\rm{SelfConfidence}}}_{i,t} \sim {{\rm{SymptomLog}}}_{i,t+1}\in \{0,1\}+{{\rm{SymptomLog}}}_{i,t-1}\in \{0,1\}+\left(1|i\right)-$$

$$i={individual},t={time}\,{point}$$

5 $${{\rm{Metacognitive\; Bias}}}_{i,t} \sim {{\rm{SymptomLog}}}_{i,t+1}\in \{0,1\}+{{\rm{SymptomLog}}}_{i,t-1}\in \{0,1\}+\left(1|i\right)-$$

$$i={individual},\,t={time}\,{point}$$

To corroborate our results showing reduced self-confidence is associated with future symptom logs, we also assessed the effects of self-confidence over OCD severity ratings during timed notifications using linear mixed-effect models (see Supplementary Note 3 and Fig. S9).

To verify that self-confidence and metacognitive bias are indeed temporally interconnected, we fit two linear models with metacognitive bias as the dependent variable and self-confidence as the independent variable, while controlling for state variables (Eq. 6) and for task variables (Eq. 7). These models did not include random intercepts as all dependent and independent variables were already within-person centred. Models were fitted using the lm function in base R.

6 $${{\rm{Metacognitive\; Bias}}}_{i,t} \sim {{\rm{SelfConfidence}}}_{i,t}+{\rm{OCD\; severity}}+{{\rm{Anxiety}}}_{i,t}{+{\rm{Happiness}}}_{i,t}+{+{\rm{Brain\; Fog}}}_{i,t}{+{\rm{Sleep\; Quality}}}_{i,t}-$$

$$i={individual},\,t={time}\,{point}$$

7 $${{\rm{Metacognitive\; Bias}}}_{i,t} \sim {{\rm{SelfConfidence}}}_{i,t}+{\rm{Staircased}}{{\rm{Accuracy}}}_{i,t}{+{\rm{Choice\; RT}}}_{i,t}+{+{\rm{Signal\; Strength}}}_{i,t}-$$

$$i={individual},\,t={time}\,{point},\,{RT}={Reaction}\,{Time}$$

#### Robustness tests

We conducted several robustness tests to ensure the reliability of the results. (1) We checked if study compliance (operationalised as the proportion of notifications answered by each participant) impacted our findings. To do this, we inspected whether compliance correlated with any averaged state and task measures, and if so, we repeated our regression analyses controlling for compliance (operationalised as the number of notifications answered throughout the assessment period). Next, (2) we repeated models accounting for different slopes per participant, although several models did not reach convergence. Results were maintained for the majority of models when per-participant slopes were included. Additionally, (3) we reran our models controlling for age (z-scored) and gender (‘Female’ or not) and found that all significant results were maintained. (4) We found that time-of-day, day-of-week and current context (i.e. current activities) variables were significantly associated with OCD severity ratings, self-confidence and symptom logging (Figs. S5–S7). We also repeated relevant models controlling for these elements. Next, (5) to further assess robustness of the symptom log results, we repeated our analyses using different symptom log time windows (i.e. within 3, 2 and 1 h of a state/task notification). See Supplementary Note 4 and Tables S8, S9 for results of these tests.

Linear model validity was determined through inspection of approximate normality of residuals and assessing collinearity of predictors (variance inflation factor >5). We checked for under- or over-dispersion of residuals when fitting logistic models using the *blmeco*^62^ R package.

As metacognitive bias emerged as the strongest task predictor of symptom logs, we checked the reliability of this measure by calculating the intraclass correlation coefficient between each pair of games (e.g., 1 and 2, 2 and 8, etc.) and computed the average per pair across all participants (see Supplementary Note 5 and Fig. S10).

#### Modelling metacognitive sensitivity

We estimated metacognitive sensitivity, meta-*d’*^63^ based on Signal Detection Theory^31^, using the cpm toolbox (Dome et al.^64^, version 0.21.4. For full model description, see Maniscalco & Lau^63^. Briefly, the model is designed to quantify how well people differentiate between correct and incorrect decisions as indexed by the confidence attributed to choices. This is captured by the model parameter, meta-*d’*, where larger values correspond to better differentiation, and smaller values correspond to worse differentiation. Metacognitive efficiency (meta-d’/d’), used in our analysis, is simply the ratio of meta-d’ to d’ (perceptual sensitivity), which enables comparisons across participants with different d’ values. The model used also specifies other parameters, collectively called meta-*c*_*2*_, which are a set of response thresholds the model uses in combination with meta-*d* to provide confidence ratings. These thresholds essentially determine the propensity to endorse judgements with a certain degree of confidence by the model.

#### Exclusions from modelling

Estimating meta-*d’* requires the discretisation of continuous confidence ratings that underlies our meta-*c*_*2*_ estimates. In our case, this meant binning the confidence ratings of each completed session into four distinct bins. This comes with a caveat that if participants had too many extreme values in both the lowest and highest bins, we could not estimate meta-*d’*. Hence, we excluded each session where the 10th and 25th percentile being equal coincided with the 75th and 90th percentile being equal for the confidence bins. Furthermore, we excluded participant’s session if their confidence rating for a given session had a very low variance (SD < 5) or extreme mean (M < 5 or M > 95). Overall, this resulted in the exclusion of 162 sessions (including the complete data set of 4 participants).

Considering the estimation of meta-d’/d’ was conducted for secondary analysis, and estimation of metacognitive bias (our task primary variable) does not require fitting this model and is robust to such distributional irregularities, we retained all sessions for analyses of metacognitive bias to avoid unnecessary loss of power.

#### Metacognitive model fitting procedure

Following previous work^63^, for metacognitive sensitivity estimation, we adjusted the model parameters to minimise the difference between human confidence ratings and the probability of those ratings as output by the model, quantified via the negative log-likelihood. This was done for each session the participants completed. We searched for the best-fitting model parameters via Trust-Region Methods^65^, which is an iterative algorithm that finds the parameter values with the lowest negative log-likelihood, the one best approximating human responses.

### Reporting summary

Further information on research design is available in the Nature Portfolio Reporting Summary linked to this article.

## Supplementary information

Supplementary Information

Reporting Summary

Transparent Peer Review File

## Data Availability

All deidentified data generated in this study are deposited in an Open Science Framework database here: 10.17605/OSF.IO/X7H5K. This includes trait questionnaire data, ecological momentary assessment data and cognitive task data.

## Code availability

All code for aggregating data, running analyses and computational modelling are available in this GitHub repository: 10.5281/zenodo.21158125.

## References

[1] Chew, B. et al. Endogenous fluctuations in the dopaminergic midbrain drive behavioral choice variability. *Proc. Natl. Acad. Sci. USA* **116**, 18732–18737 (2019). 31451671 10.1073/pnas.1900872116 PMC6744888

[2] Hewitt, S. R. C., Norbury, A., Huys, Q. J. M. & Hauser, T. U. Day-to-day fluctuations in motivation drive effort-based decision-making. *Proc. Natl. Acad. Sci. USA* **122**, 1–11 (2025). 10.1073/pnas.2417964122 PMC11962463 40096607

[3] Vidal Bustamante, C. M. et al. Within-person fluctuations in stressful life events, sleep, and anxiety and depression symptoms during adolescence: a multiwave prospective study. *J. Child Psychol. Psychiatry Allied Discip.* **61**, 1116–1125 (2020). 10.1111/jcpp.13234 PMC7494581 32185808

[4] Nota, J. A., Gibb, B. E. & Coles, M. E. Obsessions and time of day: a self-monitoring study in individuals with obsessive-compulsive disorder. *J. Cogn. Psychother.* **28**, 134–144 (2014). 32759112 10.1891/0889-8391.28.2.134

[5] Beck, A. *Cognitive Therapy and the Emotional Disorders* (Penguin, 1979).

[6] Robbins, T. W., Gillan, C. M., Smith, D. G., de Wit, S. & Ersche, K. D. Neurocognitive endophenotypes of impulsivity and compulsivity: towards dimensional psychiatry. *Trends Cogn. Sci.*10.1016/j.tics.2011.11.009 (2012). 10.1016/j.tics.2011.11.009 22155014

[7] Robbins, T. W., Vaghi, M. M. & Banca, P. Obsessive-compulsive disorder: puzzles and prospects. *Neuron* **102**, 27–47 (2019). 30946823 10.1016/j.neuron.2019.01.046

[8] Levy, N. Obsessive–compulsive disorder as a disorder of attention. *Mind Lang.* **33**, 3–16 (2018).

[9] Fleming, S. M., Dolan, R. J. & Frith, C. D. Metacognition: computation, biology and function. *Philos. Trans. R. Soc. B Biol. Sci.* **367**, 1280–1286 (2012). 10.1098/rstb.2012.0021 PMC3318771 22492746

[10] Flavell, J. H. Metacognition and cognitive monitoring: a new area of cognitive–developmental inquiry. *Am. Psychol.* **34**, 906 (1979).

[11] Fox, C. A. et al. An observational treatment study of metacognition in anxious-depression. *Elife* **12**, 1–17 (2023). 10.7554/eLife.87193 PMC10567110 37818942

[12] Hoven, M. et al. Abnormalities of confidence in psychiatry: an overview and future perspectives. *Transl. Psychiatry* **9**, 268 (2019). 10.1038/s41398-019-0602-7 PMC6803712 31636252

[13] Vaghi, M. M. et al. Compulsivity reveals a novel dissociation between action and confidence. *Neuron* **96**, 348–354 (2017). 28965997 10.1016/j.neuron.2017.09.006 PMC5643443

[14] Seow, T. X. F., Fleming, S. M. & Hauser, T. U. Metacognitive biases in anxiety-depression and compulsivity extend across perception and memory. *PLoS Ment. Health* **2**, e0000259 (2025). 41661914 10.1371/journal.pmen.0000259 PMC12798496

[15] Biria, M. et al. Excessive checking in obsessive-compulsive disorder: neurochemical correlates revealed by 7T magnetic resonance spectroscopy. *Biol. Psychiatry Glob. Open Sci.* **4**, 363–373 (2024). 38298778 10.1016/j.bpsgos.2023.08.009 PMC10829650

[16] Seow, T. X. F., Jelinek, L., Mortiz, S. & Hauser, T. U. Towards an integrative understanding of metacognitive mechanisms in psychopathology. *Nat. Rev. Psychol*. **5**, 9–28 (2026).

[17] Foa, E. B., Kozak, M. J., Salkovskis, P. M., Coles, M. E. & Amir, N. The validation of a new obsessive-compulsive disorder scale: the obsessive-compulsive inventory. *Psychol. Assess*. 10.1037/1040-3590.10.3.206 (1998).

[18] Halverson, T. F. et al. Interpersonal stress and nonsuicidal self-injury disorder in veterans: an ecological momentary assessment study. *Suicide Life Threat. Behav.* **53**, 546–556 (2023). 37052380 10.1111/sltb.12963 PMC10523856

[19] Briones-Buixassa, L. et al. Predicting non-suicidal self-injury in young adults with and without borderline personality disorder: a multilevel approach combining ecological momentary assessment and self-report measures. *Psychiatr. Q.* **92**, 1035–1054 (2021). 33475912 10.1007/s11126-020-09875-7

[20] Provenza, N. R. et al. Long-term ecological assessment of intracranial electrophysiology synchronized to behavioral markers in obsessive-compulsive disorder. *Nat. Med.* **27**, 2154–2164 (2021). 34887577 10.1038/s41591-021-01550-z PMC8800455

[21] Schneider, S. & Stone, A. A. Distinguishing between frequency and intensity of health-related symptoms from diary assessments. *J. Psychosom. Res.* **77**, 205–212 (2014). 25149030 10.1016/j.jpsychores.2014.07.006 PMC4265366

[22] Lochner, C. et al. Comorbidity in obsessive-compulsive disorder (OCD): a report from the International College of Obsessive-Compulsive Spectrum Disorders (ICOCS). *Compr. Psychiatry* **55**, 1513–1519 (2014). 25011690 10.1016/j.comppsych.2014.05.020

[23] Carter, A. S., Pollock, R. A., Suvak, M. K. & Pauls, D. L. Anxiety and major depression comorbidity in a family study of obsessive-compulsive disorder. *Depress. Anxiety* **20**, 165–174 (2004). 15643633 10.1002/da.20042

[24] Rosenberg, M. Rosenberg self-esteem scale (RSE). *Acceptance and commitment therapy*. *Measures package* **61**, 18 (1965).

[25] Dittrich, W. H., Johansen, T. & Fineberg, N. A. Cognitive Assessment Instrument of Obsessions and Compulsions (CAIOC−13)—a new 13-item scale for evaluating functional impairment associated with OCD. *Psychiatry Res.* **187**, 283–290 (2011). 21122922 10.1016/j.psychres.2010.10.031

[26] Cornsweet, T. The staircase-method in psychophysics. *Am. J. Psychol.* **75**, 485–491 (1962). 13881416

[27] Rouault, M., Seow, T., Gillan, C. M. & Fleming, S. M. Psychiatric symptom dimensions are associated with dissociable shifts in metacognition but not task performance. *Biol. Psychiatry* **84**, 443–451 (2018). 29458997 10.1016/j.biopsych.2017.12.017 PMC6117452

[28] Fleming, S. M., Ryu, J., Golfinos, J. G. & Blackmon, K. E. Domain-specific impairment in metacognitive accuracy following anterior prefrontal lesions. *Brain,*10.1093/brain/awu221 (2014). 10.1093/brain/awu221 PMC4163038 25100039

[29] Katyal, S., Huys, Q. J. M., Fleming, S. M. & Dolan, R. J. Distorted learning from local metacognition supports transdiagnostic underconfidence. *Nat. Commun*. 1–16, 10.1038/s41467-025-57040-0 (2025). 10.1038/s41467-025-57040-0 PMC11845503 39984460

[30] Fleming, S. M. & Lau, H. C. How to measure metacognition. *Front. Hum. Neurosci.* **8**, 1–9 (2014). 25076880 10.3389/fnhum.2014.00443 PMC4097944

[31] Green, D. & Swets, J. *Signal Detection Theory and Psychophysics* (Wiley, 1966).

[32] Seow, T. X. F., Jelinek, L., Moritz, S. & Hauser, T. U. Integrating cognitive neuroscience and clinical perspectives on metacognitive mechanisms in psychopathology. *Nat. Rev. Psychol.* **5**, 9–28 (2026).

[33] Weil, L. G. et al. The development of metacognitive ability in adolescence. *Conscious. Cogn.* **22**, 264–271 (2013). 23376348 10.1016/j.concog.2013.01.004 PMC3719211

[34] Marzuki, A. A. et al. Atypical action updating in a dynamic environment associated with adolescent obsessive–compulsive disorder. *J. Child Psychol. Psychiatry Allied Discip*. 10.1111/jcpp.13628 (2022). 10.1111/jcpp.13628 PMC9790358 35537441

[35] Hauser, T. U. et al. Metacognitive impairments extend perceptual decision making weaknesses in compulsivity. *Sci. Rep*. **7**, 6614 (2017). 10.1038/s41598-017-06116-z PMC5529539 28747627

[36] Hoven, M., Rouault, M., Van Holst, R. & Luigjes, J. Differences in metacognitive functioning between obsessive-compulsive disorder patients and highly compulsive individuals from the general population. *Psychol. Med.* **53**, 7933–7942 (2023). 37553980 10.1017/S003329172300209X PMC10755250

[37] Seow, T. X. F., Rouault, M., Gillan, C. M. & Fleming, S. M. How local and global metacognition shape mental health. *Biol. Psychiatry* **90**, 436–446 (2021). 34334187 10.1016/j.biopsych.2021.05.013

[38] Hoven, M., Luigjes, J., Denys, D., Rouault, M. & van Holst, R. J. How do confidence and self-beliefs relate in psychopathology: a transdiagnostic approach. *Nat. Ment. Health* **1**, 337–345 (2023).

[39] Baird, B., Smallwood, J., Gorgolewski, K. J. & Margulies, D. S. Medial and lateral networks in anterior prefrontal cortex support metacognitive ability for memory and perception. *J. Neurosci.* **33**, 16657–16665 (2013). 24133268 10.1523/JNEUROSCI.0786-13.2013 PMC6618531

[40] Saccenti, D. et al. Neural correlates of metacognition: disentangling the brain circuits underlying prospective and retrospective second-order judgments through noninvasive brain stimulation. *J. Neurosci. Res.* **102**, 1–18 (2024). 10.1002/jnr.25330 38622870

[41] Vaghi, M. M. et al. Specific frontostriatal circuits for impaired cognitive flexibility and goal-directed planning in obsessive-compulsive disorder: evidence from resting-state functional connectivity. *Biol. Psychiatry*, 10.1016/j.biopsych.2016.08.009 (2017). 10.1016/j.biopsych.2016.08.009 PMC6020061 27769568

[42] Bennett, D., Silverstein, M. S. & Niv, Y. The two cultures of computational psychiatry. *JAMA Psychiatry* **76**, 563–564 (2022). 10.1001/jamapsychiatry.2019.0231 PMC8813275 31017638

[43] Radomsky, A. S. et al. When it’s at: an examination of when cognitive change occurs during cognitive therapy for compulsive checking in obsessive-compulsive disorder. *J. Behav. Ther. Exp. Psychiatry* **67**, 101442 (2020). 30573211 10.1016/j.jbtep.2018.12.003

[44] Kiverstein, J., Rietveld, E., Slagter, H. A. & Denys, D. Obsessive compulsive disorder: a pathology of self-confidence? *Trends Cogn. Sci.* **23**, 369–372 (2019). 30954404 10.1016/j.tics.2019.02.005

[45] Jelinek, L., Faissner, M., Moritz, S. & Kriston, L. Long-term efficacy of metacognitive training for depression (D-MCT): a randomized controlled trial. 245–259, 10.1111/bjc.12213 (2019). 10.1111/bjc.12213 30556583

[46] Tilley, P. J. M. & Rees, C. S. A clinical case study of the use of ecological momentary assessment in obsessive compulsive disorder. *Front. Psychol.* **5**, 1–8 (2014). 24860521 10.3389/fpsyg.2014.00339 PMC4029001

[47] Fritz, J. et al. So you want to do ESM? 10 essential topics for implementing the experience-sampling method. 10.1177/25152459241267912 (2024).

[48] Foa, E. B. et al. The obsessive-compulsive inventory: development and validation of a short version. *Psychol. Assess.* **14**, 485–496 (2002). 12501574

[49] Patton, J. H., Stanford, M. S. & Barratt, E. S. Factor structure of the Barratt Impulsiveness Scale. *J. Clin. Psychol.* **51**, 768–774 (1995). 8778124 10.1002/1097-4679(199511)51:6<768::aid-jclp2270510607>3.0.co;2-1

[50] Buhr, K. & Dugas, M. J. The intolerance of uncertainty scale: psychometric properties of the English version. *Behav. Res. Ther.* **40**, 931–945 (2002). 12186356 10.1016/s0005-7967(01)00092-4

[51] Spitzer, R. L., Kroenke, K. & Williams, J. B. W. Validation and utility of a self-report version of PRIME-MD. *Prim. Care Companion J. Clin. Psychiatry* **2**, 31 (2000).

[52] Spielberger, C. D. State-Trait Anxiety Inventory for Adults (STAI-AD) [Database record]. *APA PsycTests*10.1037/t06496-000 (1983).

[53] Braga, R. et al. The use of ecological momentary assessment methods and designs in the context of obsessive-compulsive disorder: a systematic review. *J. Obs. Compuls. Relat. Disord*. **45**, 100952 (2025).

[54] Matcham, F. et al. Remote Assessment of Disease and Relapse in Major Depressive Disorder (RADAR-MDD): a multi-centre prospective cohort study protocol. *BMC Psychiatry* **19**, 1–11 (2019). 30777041 10.1186/s12888-019-2049-z PMC6379954

[55] Starr, L. R. & Davila, J. Temporal patterns of anxious and depressed mood in generalized anxiety disorder: a daily diary study. *Behav. Res. Ther.* **50**, 131–141 (2012). 22196213 10.1016/j.brat.2011.11.005 PMC3390195

[56] Heininga, V. E. et al. The dynamical signature of anhedonia in major depressive disorder: positive emotion dynamics, reactivity, and recovery. *BMC Psychiatry* **19**, 1–11 (2019). 30736751 10.1186/s12888-018-1983-5 PMC6368777

[57] Paterson, J. L., Reynolds, A. C., Ferguson, S. A. & Dawson, D. Sleep and obsessive-compulsive disorder (OCD). *Sleep. Med. Rev.* **17**, 465–474 (2013). 23499210 10.1016/j.smrv.2012.12.002

[58] Andrillon, T., Lutz, A., Windt, J. & Demertzi, A. Where is my mind? A neurocognitive investigation of mind blanking. *Trends Cogn. Sci.* **xx**, 1–14 (2025). 10.1016/j.tics.2025.02.002 40280833

[59] Brierley, M. E. E. et al. How do daily routines and situational factors affect the severity of obsessive-compulsive disorder? *J. Psychiatr. Res.* **143**, 395–399 (2021). 34600268 10.1016/j.jpsychires.2021.09.040

[60] Moses-Payne, M. E., Habicht, J., Bowler, A., Steinbeis, N. & Hauser, T. U. I know better! Emerging metacognition allows adolescents to ignore false advice. *Dev. Sci.* **24**, 1–13 (2021). 10.1111/desc.13101 PMC8612133 33686737

[61] Bates, D., Maechler, M., Bolker, B. & Walker, S. lme4: linear mixed-effects models using Eigen and S4. R package version 1.1-7, http://CRAN.R-project.org/package=lme4. R Package version (2014).

[62] Korner-Nievergelt, F. et al. *Bayesian Data Analysis in Ecology Using Linear Models with R, BUGS, and Stan* (Academic Press, 2015).

[63] Maniscalco, B. & Lau, H. A signal detection theoretic approach for estimating metacognitive sensitivity from confidence ratings. *Conscious. Cogn.* **21**, 422–430 (2012). 22071269 10.1016/j.concog.2011.09.021

[64] Dome, L. et al. cpm: a python library for theory-driven modelling in computational psychiatry. *PLOS Computational Biology* **22**, e1014481 (2026). 10.1371/journal.pcbi.1014481 PMC13379104 42441721

[65] Conn, A. R., Gould, N. I. & Toint, P. L. *Trust Region Methods* (Society for Industrial and Applied Mathematics, 2000).

