# Supplementary material for: Drops in confidence precede symptoms of obsessive-compulsive disorder
Source: Nat Commun. 2026 Aug 13;17:8333. doi: 10.1038/s41467-026-76671-5 (PMC13473584; doi:10.1038/s41467-026-76671-5)
Supplement: Supplementary file 1 [file 41467_2026_76671_MOESM1_ESM.pdf]

Drops in confidence precede symptoms of OCD

Supplementary Information

A.A. Marzuki[1,2,3], L.A. Kosina[1], L. Dome[1,2], S.R.C. Hewitt[4,5], T.U.Hauser[1,2,4]

1. Department of Psychiatry and Psychotherapy, Faculty of Medicine, Tübingen University, Tübingen, Germany

2. German Center for Mental Health (DZPG), Tübingen, Germany

3. Department of Neuroimaging, Institute of Psychology, Psychiatry & Neuroscience, King's College London, United Kingdom

4. Max Planck UCL Centre for Computational Psychiatry and Ageing Research, University College London, UK

5. Functional Imaging Laboratory, Department of Imaging Neuroscience, University College London, London WC1N 3AR, United Kingdom

Contents

Participant Recruitment Flowchart 2
Demographic Details 3
State survey items 4
Summary of longitudinal task performance data 4
Trait-State Relationships 6
Supplementary Note 1 7
Partial correlations between state and trait variables 7
Supplementary Note 2: External Contexts Affecting Symptoms 8
Daily activities and contexts associated with OCD symptom logs, severity ratings, and self-confidence 8
Time-of-day and day-of-week analysis 10
Time-of-day and day-of-week results 11
Quantifying number of logs discarded for logistic regression analysis 13
Supplementary Note 3 13
Day-to-day OCD severity ratings are predicted by current and past self-confidence 13

Supplementary Note 4: Robustness Tests 14
Analysing significance of random subject intercepts.. 14
Robustness tests for regression models.. 15
Testing symptom log effects in shorter time windows....................................... 17
Supplementary Note 5 17
Assessing reliability of task metacognitive bias 17

Participant Recruitment Flowchart

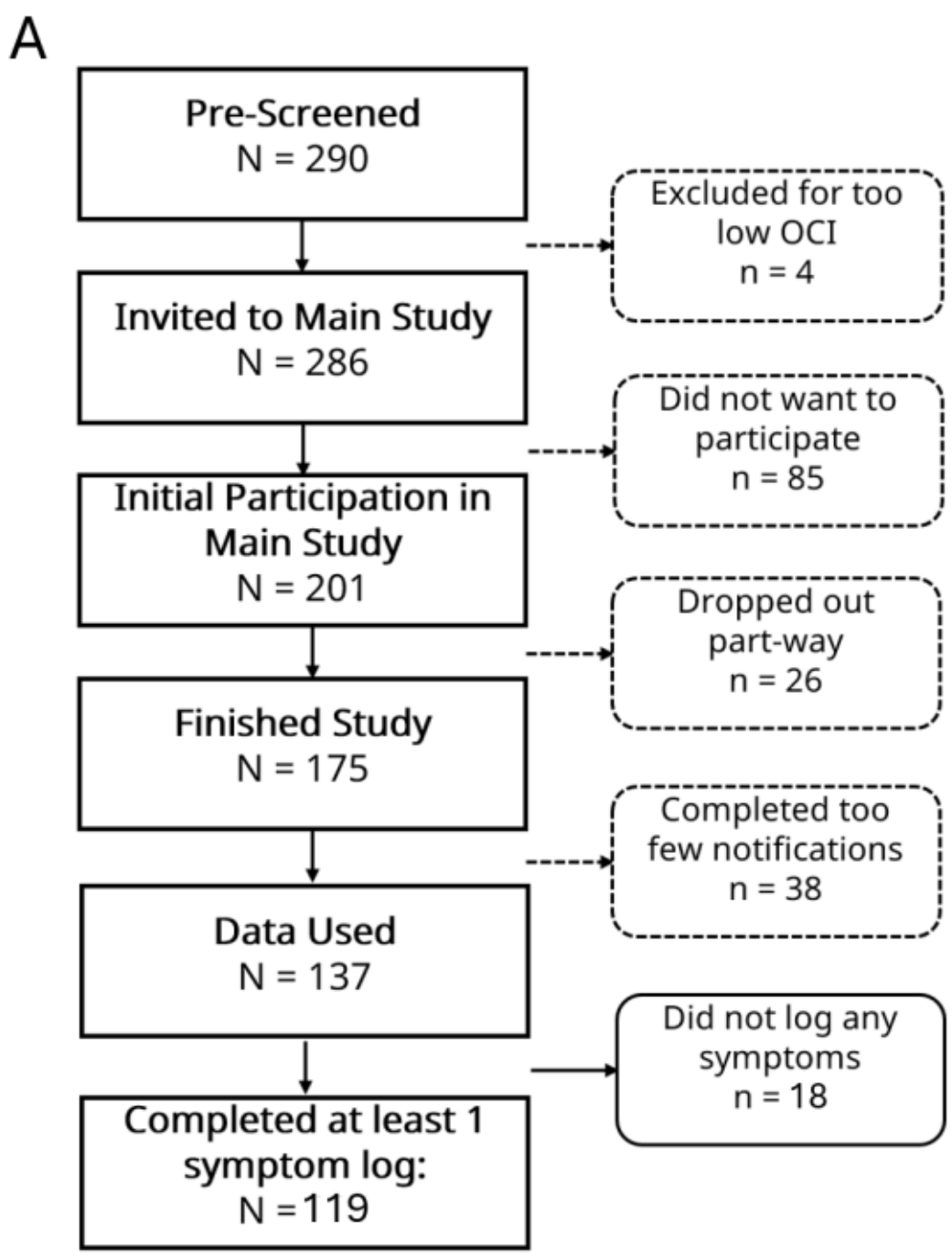

Figure S1: Flowchart of participant recruitment.

Demographic Details

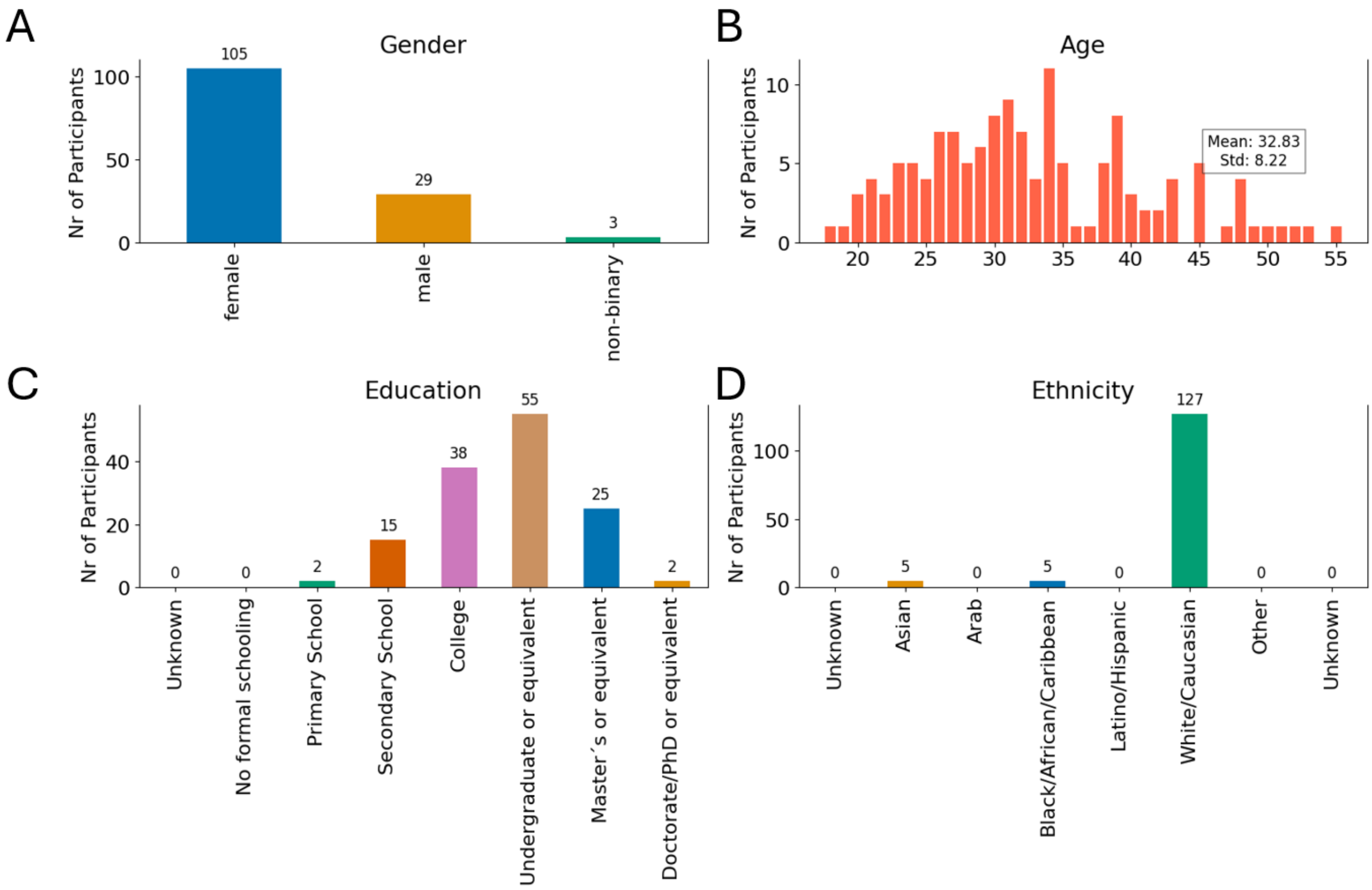

Figure S2: Plots of participant demographics, for gender (A), age (B), education level (C), and ethnicity (D).

State survey items

Table S1: Daily survey items used in EMCT study

**Item**	**Question**
Obsessive-compulsive severity	How intense are your symptoms of OCD right now?
Self-confidence	How confident do you feel right now?
Anxiety	How anxious do you feel right now?
Happiness	How happy do you feel right now?
Brain Fog	Do you have difficulty thinking clearly right now?
Sleep Quality	How well did you sleep last night?
Current Activity/Context	What are you doing right now?
Social Context	Who are you with right now?

Summary of longitudinal task performance data

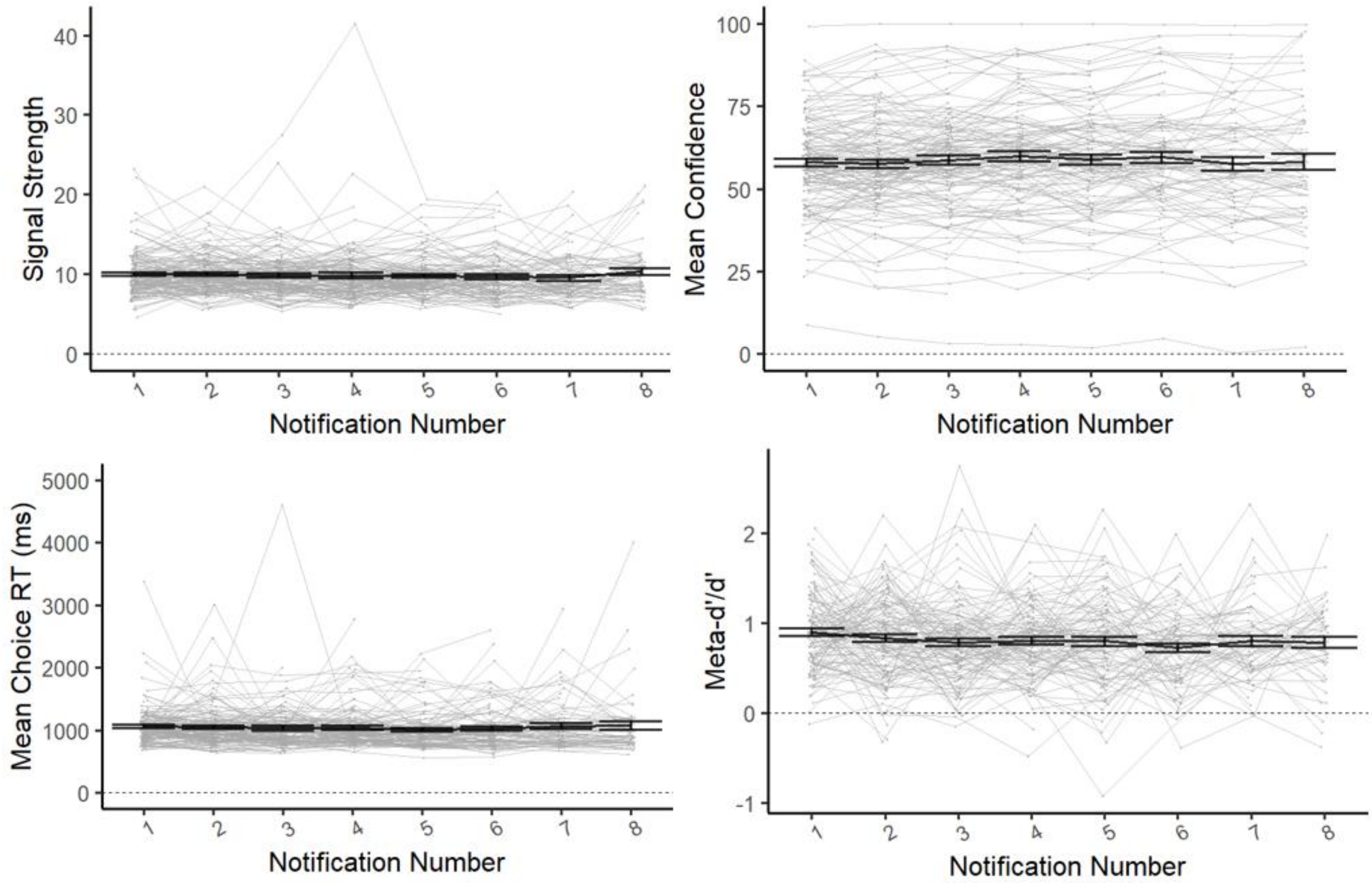

Figure S3: Plots summarising variability in task performance metrics over 8 sessions (number of times participants were notified to complete the task). The group mean ± standard error of mean (black) across notifications (x-axis) are depicted. Each grey line is an individual participant. RT (reaction times) are shown in milliseconds. Staircased accuracy is depicted in Figure 3c of the main paper.

Table S2: Mean and standard deviation of each task measure per session number

	1	2	3	4	5	6	7	8
Staircased Accuracy (proportion)	0.73 ± 0.022	0.72 ± 0.020	0.73 ± 0.023	0.72 ± 0.020	0.73 ± 0.022	0.72 ± 0.023	0.73 ± 0.022	0.71 ± 0.025
Choice Reaction Time (milliseconds)	1116 ± 659	1245 ± 1735	1079 ± 620	1140 ± 815	1012 ± 306	1151 ± 948	1239 ± 1165	1309 ± 1888
Signal Strength	10.0 ± 2.86	10.1 ± 2.53	9.87 ± 3.01	9.87 ± 3.92	9.81 ± 2.57	9.71 ± 3.05	9.57 ± 2.88	10.4 ± 3.4
Metacognitive Bias	58.0 ± 14.0	57.7 ± 15.4	58.7 ± 15.6	59.9 ± 16.0	58.9 ± 16.0	59.6 ± 16.5	57.6 ± 17.7	58.2 ± 18.9
Meta-d’/d’	0.90 ± 0.44	0.83 ± 0.48	0.79 ± 0.49	0.80 ± 0.43	0.80 ± 0.52	0.72 ± 0.43	0.80 ± 0.46	0.79 ± 0.45

Trait-State Relationships

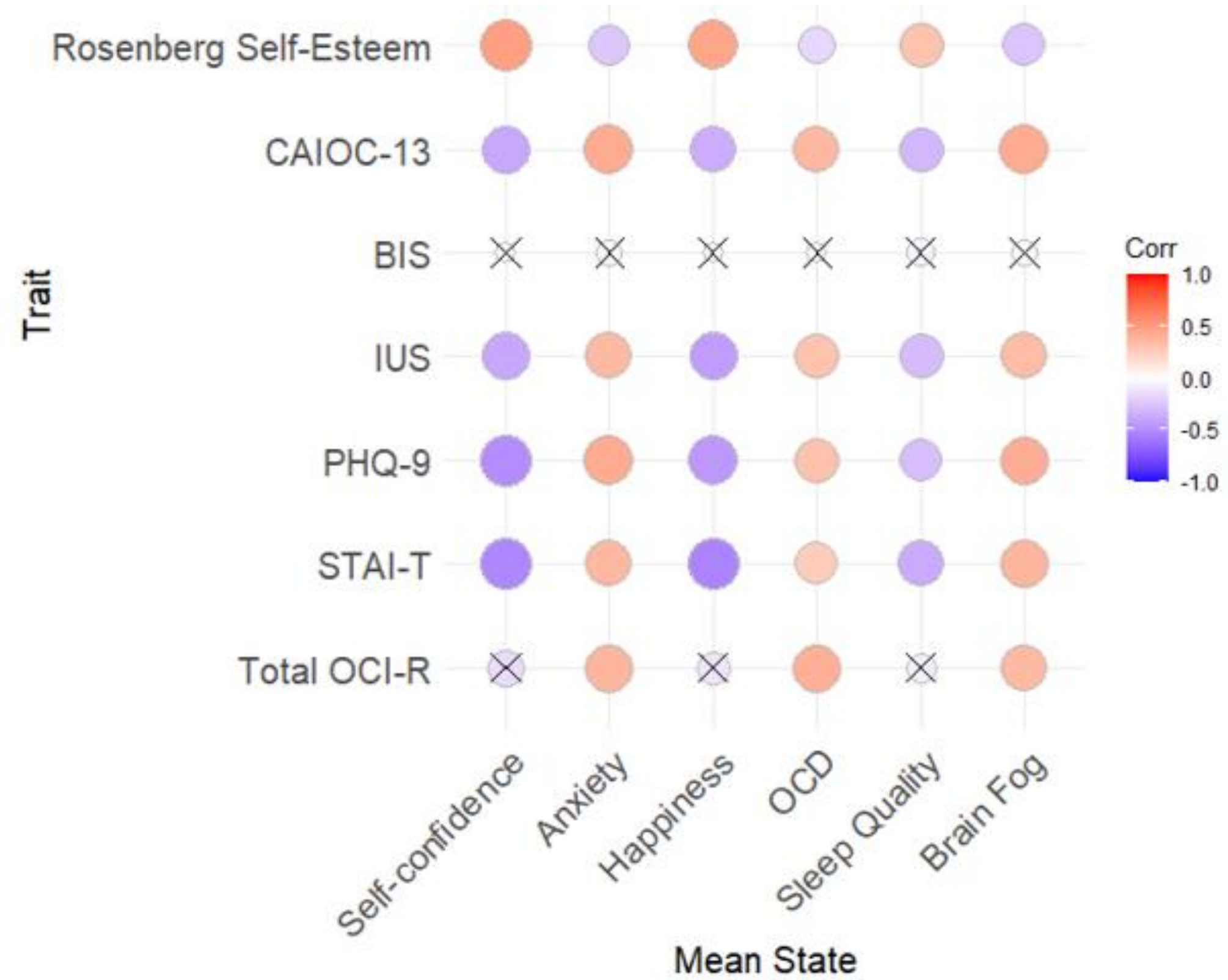

Figure S4: Correlation matrix showing overall Spearman correlations between all state measures (averaged across 14 days) and trait measures CAIOC-13: Cognitive Assessment Instrument of Obsessions and Compulsions, BIS: Barratt Impulsivity Scale, IUS: Intolerance of Uncertainty Scale, PHQ-9: Patient Health Questionnaire-9 for depression severity, STAI-T: trait anxiety scale, OCI-R: obsessive compulsive inventory-revised. 'X' indicates correlation is not statistically significant. There was no significant correlation between trait measures and averaged inter-symptom intervals.

Supplementary Note 1

Partial correlations between state and trait variables

In the main manuscript, we investigated whether the self-confidence state measure was associated with well-established trait measures. Here we report whether the relationships reported maintained statistical significance even when conducting partial Spearman correlations to control for other averaged state variables (Tables S3 – S4). All significant associations were maintained.

Table S3: Partial Spearman correlations for association between state averaged confidence and trait self-esteem (Rosenberg Self-Esteem Scale). Two-sided tests were conducted.

**Variable being controlled for**	**Statistic**
State averaged anxiety	*rho* = 0.45, p <.001
State averaged OCD severity	*rho* = 0.47, p <.001
State averaged sleep quality	*rho* = 0.42, p <.001
State averaged brain fog	*rho* = 0.46, p <.001
State averaged happiness	*rho* = 0.22, p = .009

Table S4: Partial Spearman correlations for association between state averaged confidence and perceived cognitive impairment related to OCD (Cognitive Assessment Instrument of Obsessions and Compulsions-13). Two-sided tests were conducted.

**Variable being controlled for**	**Statistic**
State averaged anxiety	*rho* = -0.27, p = .002
State averaged OCD severity	*rho* = -0.33, p < .001
State averaged sleep quality	*rho* = -0.29, p < .001
State averaged brain fog	*rho* = -0.26, p = .002
State averaged happiness	*rho* = -0.17, p = .045

We also ascertained that averaged OCD severity ratings during notification periods were significantly correlated with trait obsessive-compulsive scores (measured using OCI-R, *rho* = 0.41, *p-FDR* < .001). This was the case even when partially controlling for other state variables (Table S5).

Table S5: Partial Spearman correlations for association between state averaged OCD severity and trait obsessive-compulsive severity (Obsessive-compulsive Inventory-Revised). Two-sided tests were conducted.

**Variable being controlled for**	**Statistic**
State averaged anxiety	*rho* = 0.2, p = .01
State averaged confidence	*rho* = 0.40, p < .001
State averaged sleep quality	*rho* = 0.41, p < .001

State averaged brain fog	*rho* = 0.24, *p* = .004
State averaged happiness	*rho* = 0.40, *p* < .001

As an extra measure of robustness, we assessed whether relationships between state variables (OCD and confidence) and trait variables (Rosenburg Self-Esteem, CAIOC-13, and OCI-R) held when controlling for all other state variables (anxiety, sleep quality, brain fog, happiness) simultaneously (see Table S6) using linear models.

We found that the associations between averaged state confidence and CAIOC-13 scores (β = -8.00, 95% CI [-13.5 -2.42], *p* = .005), and between averaged state OCD and OCI-R scores (OCD: β = 6.09, 95% CI [0.84 11.35], *p* = .024) were still significant even when controlling for all other state variables However, while the direction of the relationship was maintained, the association between Rosenburg Self-Esteem scores and state averaged self-confidence only showed a trend-level significance (β = 2.66, 95% CI [-0.06 5.38], *p* = .055). Nonetheless, no other state measure emerged as being more strongly associated with trait self-esteem (state OCD: β = 0.58, *p* = .67; state anxiety: β = -0.84, *p* = .54; state sleep quality: β = 1.03, *p* = .26; state brain fog: β = -0.26, *p* = .833, state happiness: β = 1.89, *p* = .21).

Table S6: Results from linear models measuring associations between state and trait variables. Two-sided tests were conducted.

**Trait Dependent Variable**	**State Independent Variable of Interest**	**Result of State Variable of Interest**
Rosenberg Self-Esteem	Averaged Confidence	Confidence: β = 2.66, 95% CI [-0.06 5.38], *p* = .055
CAIOC-13	Averaged Confidence	Confidence: β = -8.00, 95% CI [-13.5 -2.42], *p* = .005
OCI-R	Averaged OCD	OCD: β = 6.09, 95% CI [0.84 11.35], *p* = .024

Supplementary Note 2: External Contexts Affecting Symptoms

Daily activities and contexts associated with OCD symptom logs, severity ratings, and self-confidence

To evaluate the ecological validity OCD symptom logs and ratings of OCD severity and self-confidence, we investigated whether they were meaningfully capturing external events. We examined how OCD symptom logging varied based on current activities and social contexts (such as whether participants were spending time with others or alone) – Figure S5A. All context variables were added simultaneously to regression models predicting either OCD symptom logs (logistic mixed-effects model) or self-

confidence/OCD symptom severity (linear mixed-effects models). We found people were less inclined to log symptoms during periods of rest (Odds ratio = 0.59 =, β = -0.54, p < .001). Additionally, partaking in leisure (Odds ratio = 0.70, β = -0.36, p = .003), and self-care activities (Odds ratio = 0.69, β = -0.37, p = .008) predicted a lower likelihood of symptom logs, suggesting that, intuitively, engaging in relaxing or enjoyable activities is associated with reduced OCD symptoms.

As a validation step, we confirmed that OCD severity ratings, which were recorded alongside "current context" during notified periods and were thus more frequent, showed similar associations with activities and social context (Figure S5B). Congruent with symptom log findings, OCD severity ratings were lower when participants were resting (β = -0.10, 95% CI [-0.18 -0.03], p = .006). OCD severity ratings were also elevated when participants were involved in daily routines like running errands (β = 0.26, 95% CI [0.17 0.34], p < .001), travelling/commuting (β = 0.18, 95% CI [0.05 0.31], p =.007), and working/studying (β = 0.13, 95% CI [0.03 0.22], p =.006). Whilst we cannot determine the causality of this effect, it aligns with previous reports of OCD symptoms making it difficult to complete daily tasks and driving occupational impairment[1,2], while our symptom log findings suggest that periods of rest and relaxation are important for symptom alleviation.

OCD severity ratings during notifications were also significantly linked to participating in sports or exercising (β = -0.17, 95% CI [-0.34 -0.005], p = .040), and when around other people (β = -0.07, 95% CI [-0.12 -0.02], p = .004). Inversely, OCD ratings were elevated when people reported running errands (β = 0.26, 95% CI [0.17 0.34], p < .001), travelling/commuting (β = 0.18, 95% CI [0.05 0.31], p =.007), and working/studying (β = 0.13, 95% CI [0.03 0.22], p =.006).

Self-confidence also showed fluctuations with external contexts (Figure S5C): people reported greater self-confidence during sports or exercising (β = 0.50, 95% CI [0.34 0.67], p < .001), and when with other people (β = 0.15, 95% CI [0.10 0.19], p < .001). In addition, self-confidence was elevated when partaking in leisure (e.g., watching television, reading; β = 0.11, 95% CI [0.03 0.19], p =.0042) and self-care (e.g., eating, drinking; β = 0.19, 95% CI [0.10 0.28], p < .001) activities. This suggests that, intuitively, greater self-confidence is associated with pleasurable contexts.

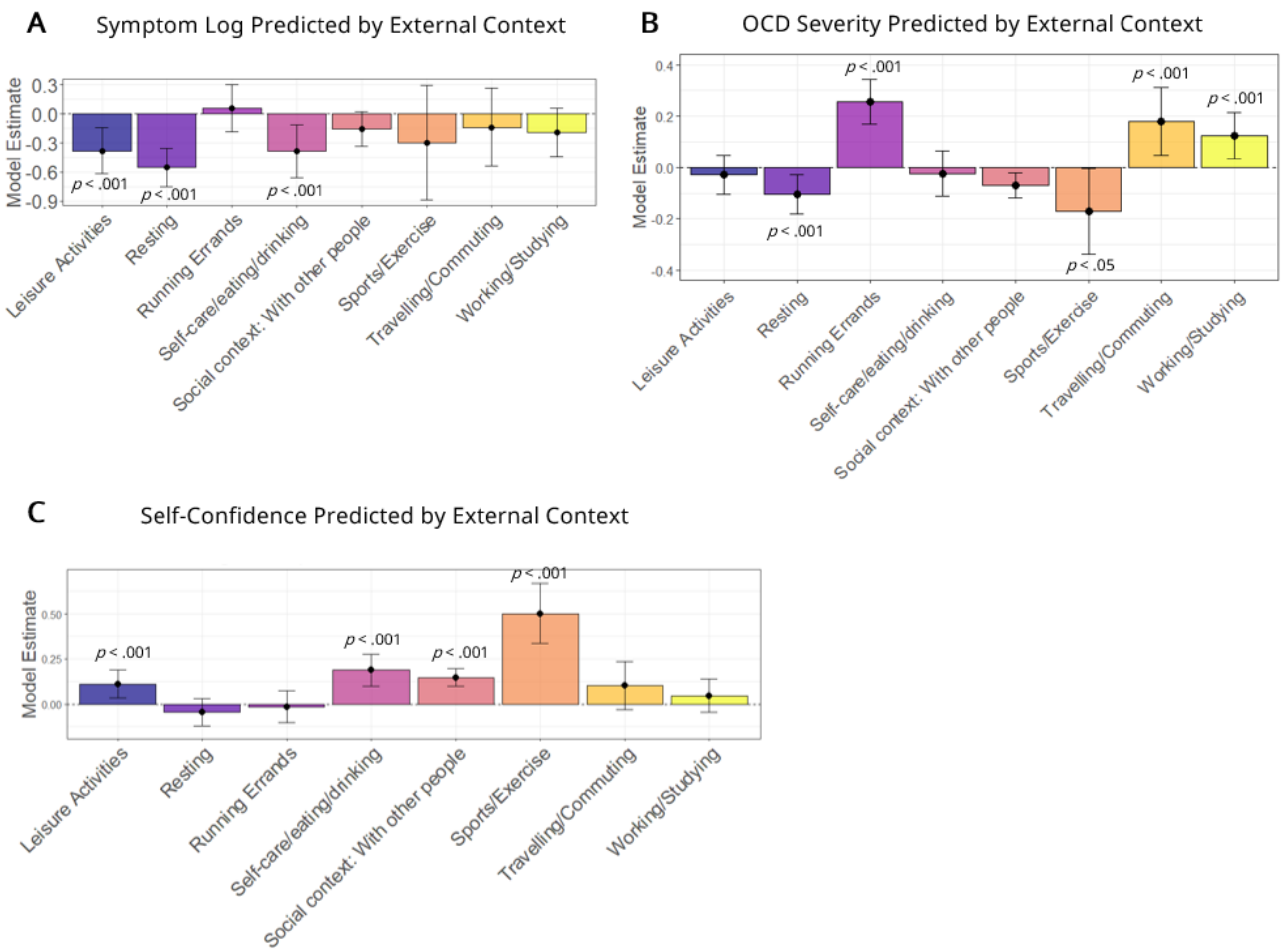

Figure S5: Bar plots depicting the estimated model coefficients obtained from linear mixed-effect models with current activity and social context (whether with other people or not) associated with ***A.*** *Symptom Logs,* ***B.*** *OCD severity, and* ***C.*** *Self-confidence. Activities and social context were entered as predictors together within the same models. Error bars = 95% confidence intervals. N for each plot = 119. Exact p-value for Sports/Exercise in C is .040.*

Time-of-day and day-of-week analysis

We tested time-of-day and day-of-week effects on symptom logs by summing the number of symptom logs per subject per day and per time. Two repeated measures ANOVAs (for time-of-day and day-of-week) were then conducted followed by post-hoc pairwise t-tests with Bonferroni adjustment. To compare symptom logging between weekdays and weekends, we first calculated the average daily number of symptoms recorded per subject. We then applied a Wilcoxon signed-rank test (due to deviations from normality assumption) to the data comparing weekdays and weekends.

When assessing day-of-week effects on moment-to-moment state variables, we dummy coded the days in which notifications were completed (e.g., for the variable 'Monday' it would be either Monday [1] or not Monday [0]). The separate days were then inserted as predictors within the same linear mixed effects model predicting self-confidence and OCD severity. In addition, we conducted a similar analysis but this time collapsing

Monday-Friday as 'Weekdays' and 'Saturday-Sunday' as weekends, to assess whether there was a general effect of weekends on state variables.

In our time-of-day analyses, we divided notification completion times into discrete periods: morning (06:00 – 11:59), afternoon (12:00 – 16:00), evening (16:00 – 20:00), night (20:00 - 23:59), and late-night (00:00 – 05:59). 'Late-night' rows were removed from this analysis due to low number of data points (only making up 1.5% of data). The times were dummy coded (e.g., the morning variable would contain either 'Morning' [1] or not [0]) and all were initially inserted into the same linear mixed-effects model. However, due to too high collinearity, we opted to insert them each into a separate model predicting self-confidence and OCD severity.

Time-of-day and day-of-week results

We found OCD symptom logging was, on average, more frequent on weekdays (per day mean = 2.34 ± 3.73) compared to weekends (per day mean = 1.95 ± 3.38), Wilcoxon's V = 4506, *p* = .008 – Figure S6. More fine-grained day-of-week analysis (i.e., dummy coding each day as Monday – Sunday) did not yield any significant patterns (all associations *p* > .05). Time-of-day was not significantly linked OCD symptom logging (all associations *p* > .05).

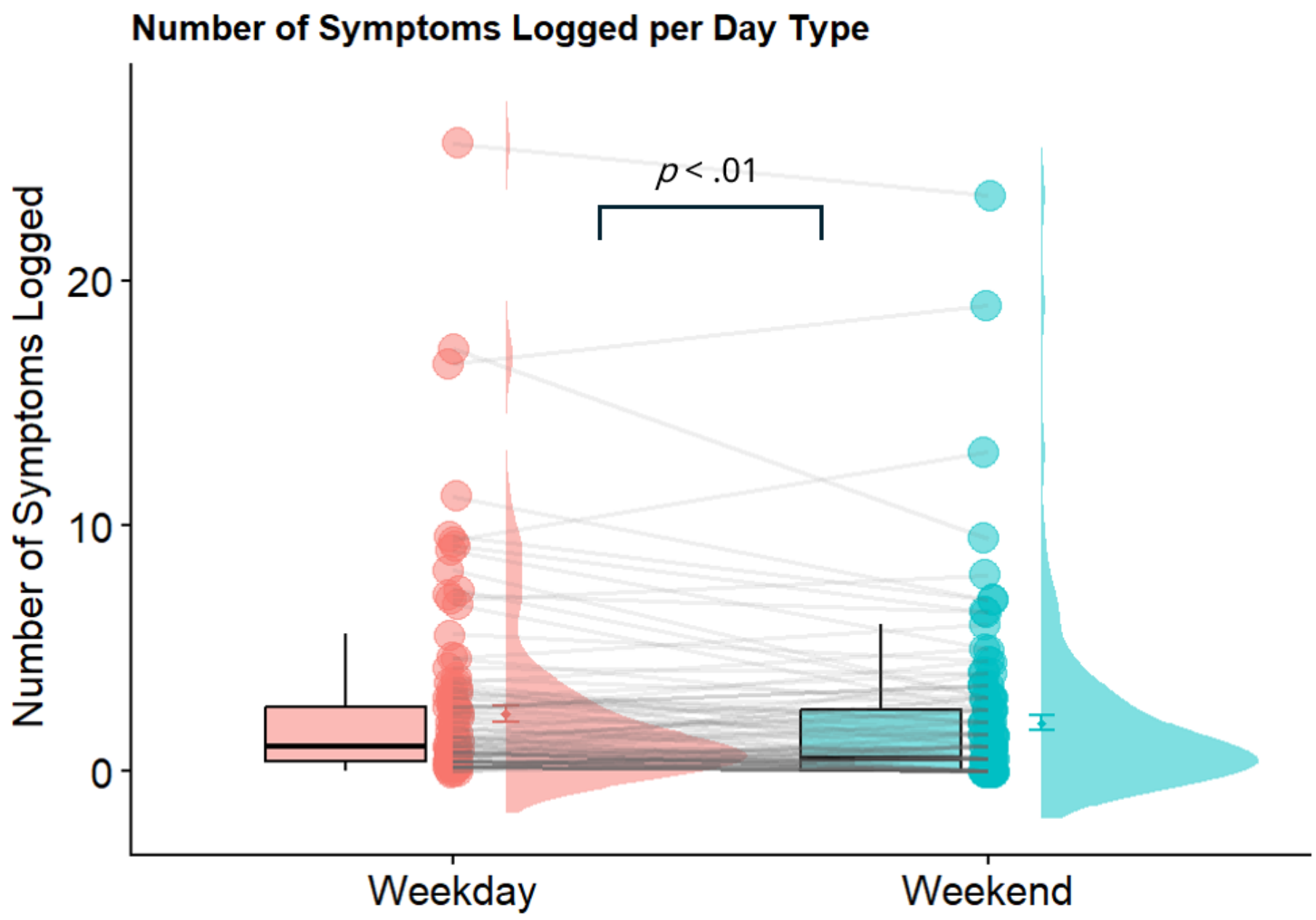

Figure S6: Mean number of symptoms logged was greater during weekdays vs weekends. Each dot represents one subject; error bars represent mean and standard errors. N = 119. Exact p-value = .008.

However, we found significant day-of-week and time-of-day fluctuations in state notified OCD severity ratings and self-confidence – likely because these variables were measured more regularly (i.e., multiple times daily) across the study period. OCD severity ratings showed a characteristic periodic structure where people experienced greater severity in the middle of the week compared to other days (Wednesdays: β = 0.14, 95% CI [0.05

0.23], *p* <.001) and lower severity during weekends (i.e., on Saturdays: β = -0.098, 95% CI [-0.19 -0.007], *p* =.034; Figure S7A). Self-confidence showed similar day-to-day variation, with lower self-confidence reported mid-week (Wednesdays: β = -0.092, 95% CI [-0.18 -0.001], *p* =.047; Figure S7B). These weekday-related effects remained significant even though we controlled for whether participants were currently at work/in employment (which may be driving weekday effects).

Moreover, similar to OCD symptom logging, we found an overall difference between weekdays and weekends when it came to state reporting: people reported that their state OCD severity was lower (Weekends compared to weekdays: β = -0.12, 95% CI [-0.18 -0.07], *p* < .001) and their state self-confidence higher (Weekends compared to weekdays: β = 0.061, 95% CI [0.006 0.06], *p* = .030) during weekends compared to weekdays.

When looking at fluctuations within a single day, OCD severity ratings (Figure S7C) and self-confidence (Figure S7D) showed dissociable daytime-effects. The latter was significantly lower in the mornings (β = -0.10, 95% CI [-0.16 -0.045], *p* < .001) while OCD severity was lower at night (β = -0.074, 95% CI [-0.016 -0.0037], *p* =.039). This is consistent with previous self-monitoring findings demonstrating that OCD peaks in midday before waning in the evening and night[3].

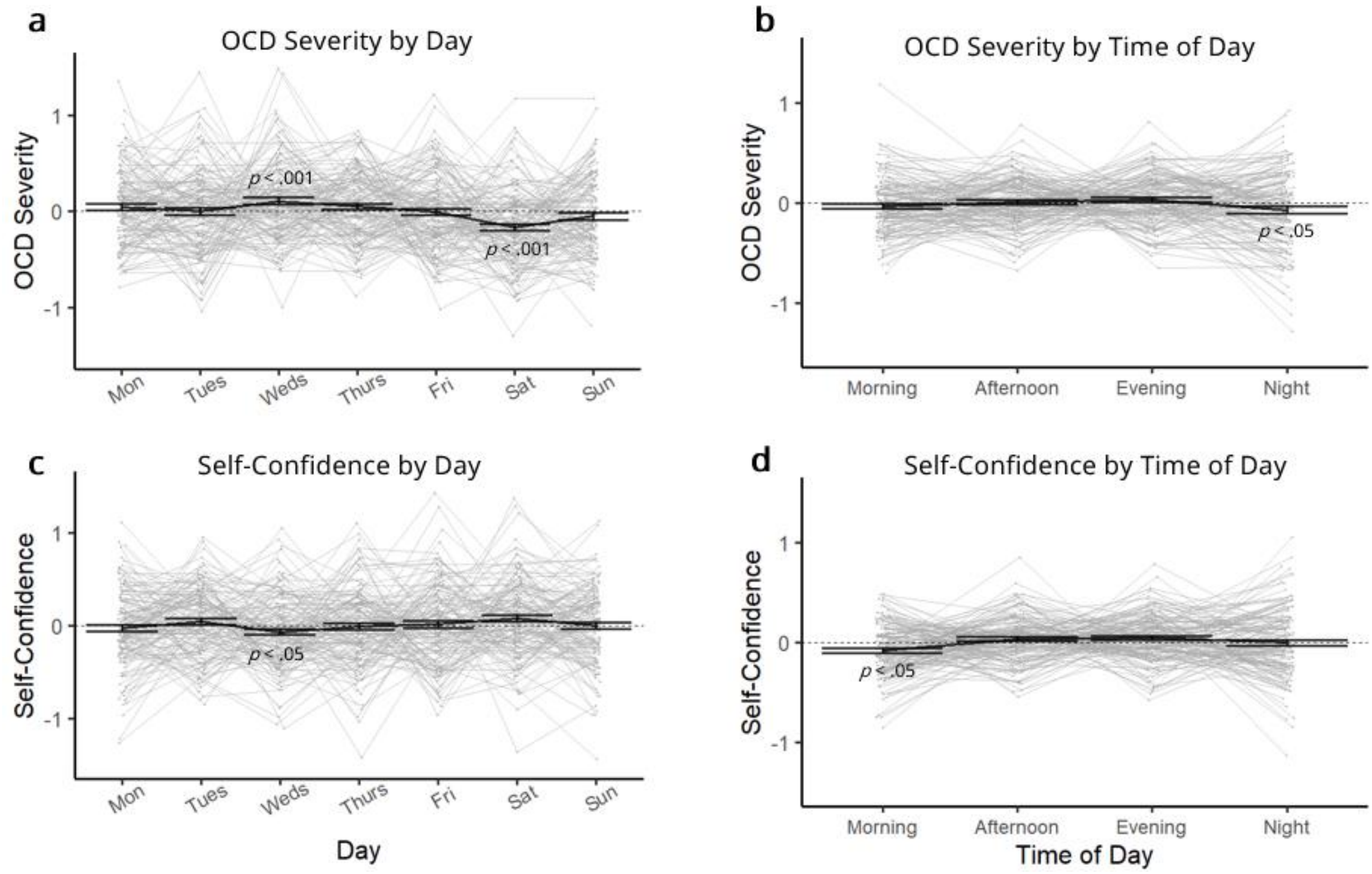

Figure S7: Fluctuations in self-confidence and OCD symptoms by time-of-day and day-of-week. Mean (normalized within-person) state (grey) and group mean ± standard error of mean (black) across days (x-axis). Each grey line is an individual participant. ***A.*** *Linear mixed-effect models revealed OCD severity was significantly worse mid-week (Wednesdays) and significantly improved on Saturdays.* ***B.*** *Mean state self-confidence variability across days. Self-*

confidence was significantly reduced on Wednesdays (exact p-value = .030). ***C.*** *Mean state OCD severity per subject and group mean by time of day. OCD severity was significantly lower at night (exact p-value = .039).* ***D.*** *Mean state self-confidence by time of day. Self-confidence was significantly lower during the mornings (p< .001). Error bars represent 95% confidence intervals. N =137.*

Quantifying number of logs discarded for logistic regression analysis

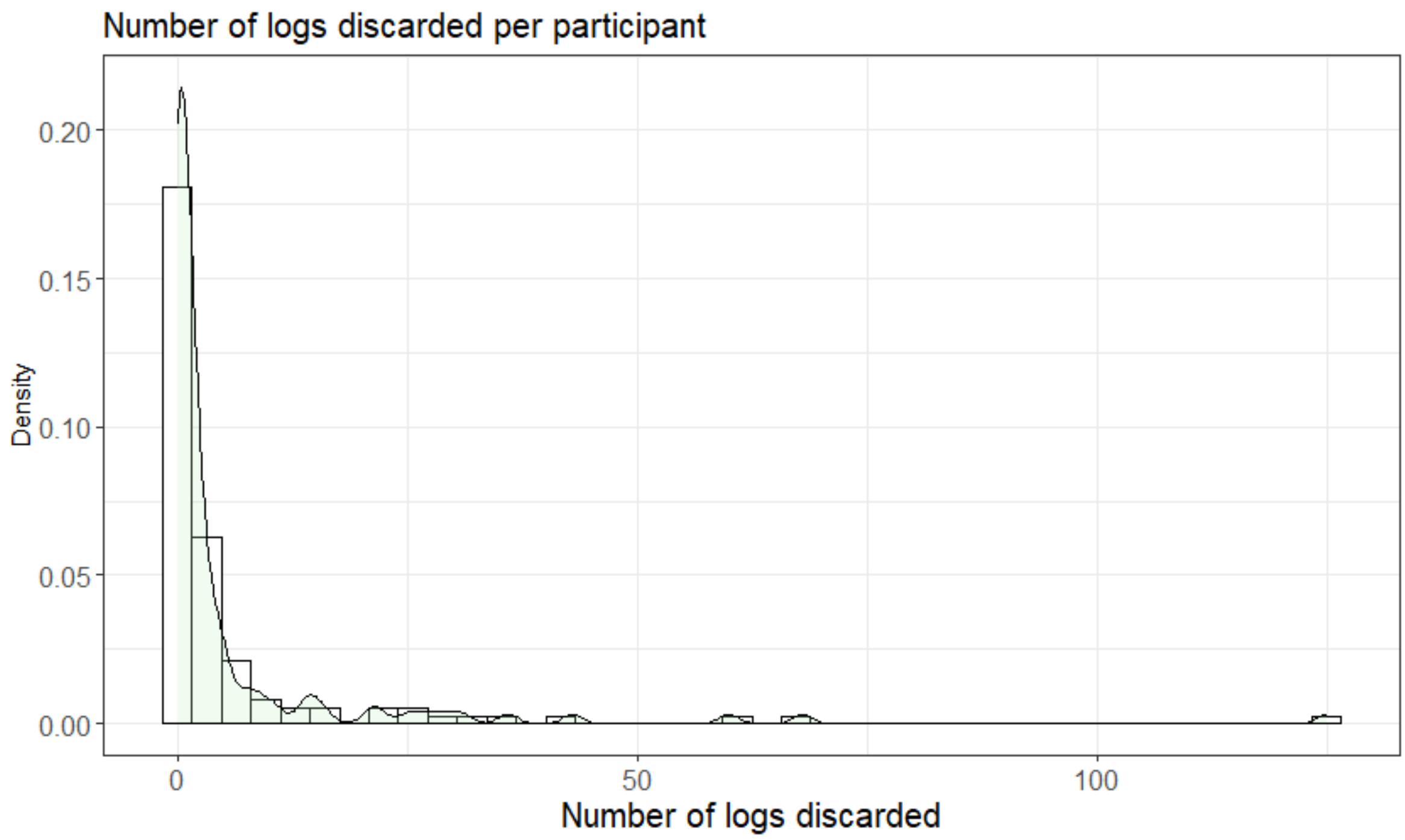

Figure S8: Histogram depicting number of symptom logs discarded per participant when running logistic regressions.

Supplementary Note 3

Day-to-day OCD severity ratings are predicted by current and past self-confidence

To corroborate our results showing reduced self-confidence is associated with future symptom logs, we assessed whether we see similar relationships when considering OCD severity ratings during timed notifications.

Linear mixed effects models showed that having low self-confidence at a given time point was significantly linked with higher OCD severity (β = -0.04, 95% CI [-0.06 -0.02], p <.001; Figure S9A). Alongside self-confidence, current OCD severity showed expected relationships with other state measures: it was associated positively with anxiety (β = 0.41, 95% CI [0.38 0.43], p < .001) and brain fog (β = 0.18, 95% CI [0.16 0.21], p < .001) and negatively with happiness (β = -0.09, 95% CI [-0.11 -0.06], p < .001) and sleep quality (entered in a separate model to prevent data loss; β = -0.19, 95% CI [-0.23 -0.14], p < .001) – Figure S9A.

Next, we were interested in the directionality of these effects, i.e. whether, like our symptom log findings, drops in self-confidence preceded increased OCD severity ratings. Self-confidence at a given moment was significantly (inversely) related to future (β = -0.03, 95% CI [-0.05 -0.009], p = .008), and present OCD severity (β = -0.34, 95% CI [-0.37 -0.32], p < .001), but not with past OCD (β = -0.009, p = .490) – Figure S9B. This suggests that reduced self-confidence drives future OCD ratings and not vice versa.

However, metacognitive bias was not significantly associated with OCD severity ratings (β = 0.040, p = .210).

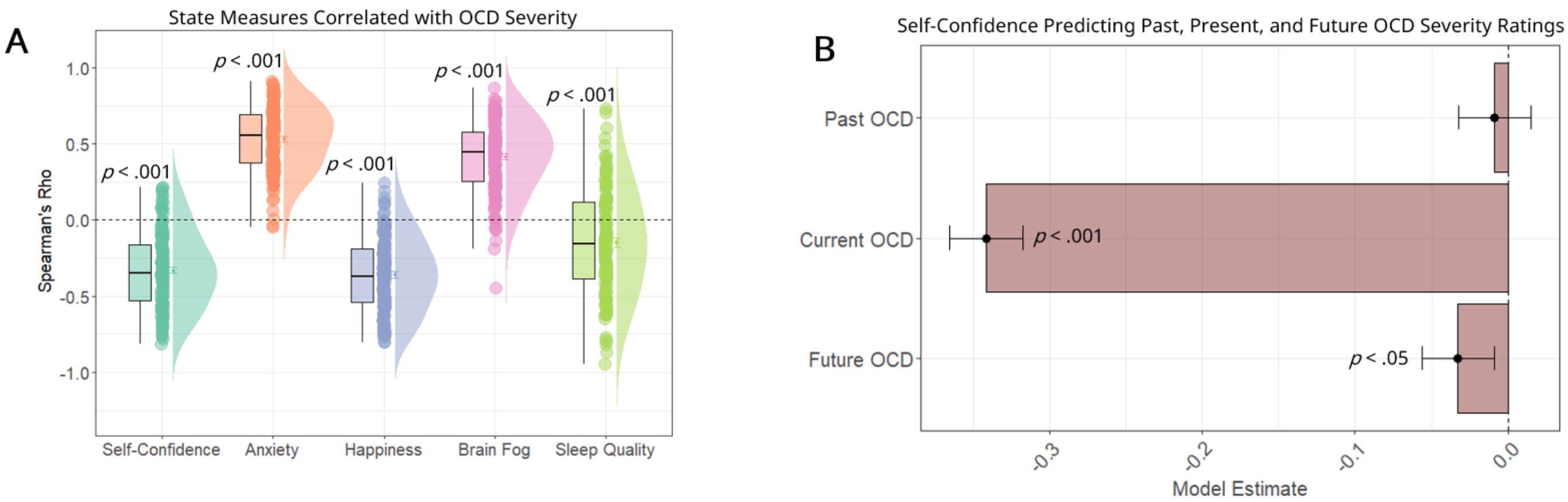

*Figure S9: State measures fluctuating with OCD severity. **A.** Raincloud plots (for visualisation purposes) showing within-subject correlations between OCD severity (a) and other state measures. Each circle represents one participant's Spearman's Rho value depicting the correlation strength between the state measure on the x-axis with their own OCD severity and self-confidence. Group mean standard error bars and boxplots are displayed. Significance stars indicate the variable was significantly predictive in a linear model (all predictors inserted at the same time) **B.** Bar plot depicting model coefficient estimates from linear model with past, current, and future OCD severity as predictors of self-confidence. Error bars represent 95% confidence intervals directional effects of self-confidence over OCD severity. Here, self-confidence predicted current and future OCD (exact p-value = .008) severity at a given moment, but past OCD did not impact current self-confidence (p = .490). N across all analyses = 137.*

<u>Supplementary Note 4: Robustness Tests</u>

Analysing significance of random subject intercepts

When assessing significance of random subject-intercepts in our main models (see Table S7 for statistics), we found that the addition of per-subject intercepts improved the fit of models where 'Future Symptom Log' was inserted as the dependent variable. However, introducing a random intercept did not enhance the performance of models that used future and past symptom logging to predict state variables. Since beta estimates were similar between models with and without the random intercept, we decided to retain our original results. Moreover, we ascertain that our findings remain significant even when fitting models without random intercepts.

Table S7: Results comparing main models with and without the random subject-intercept. Two-sided tests were conducted.

Model	Variance	Standard Deviation	Likelihood Ratio Test	Random-Intercept improved model performance?
Current OCD Severity$_{,i}$ ~ Future Symptom Log$_i$ + Past Future Log$_i$	1.17e-32	1.082e-16	$\chi^2(1) = 0$, $p = 1$	No difference.
Future Symptom Log$_{i,t}$ $\in\{0,1\}$ ~ Self-Confidence$_{t,i}$ + Other State Variables$_{t,i}$	2.72	1.51	$\chi^2(1) = 161.98$, $p < .001$	Improved
Current Self-Confidence$_{t,i}$ ~ Future Symptom Log$_i$ + Past Future Log$_i$	9.256e-33	9.621e-17	$\chi^2(1) = 0$, $p = 1$	No difference.
Future Symptom Log$_{i,t}$ $\in\{0,1\}$ ~ Task Metacognitive Bias$_{t,i}$ + Other Task Measures$_{t,i}$	2.29	1.51	$\chi^2(1) = 60.16$, $p < .001$	Improved.
Task Metacognitive Bias$_{t,i}$ ~ Future Symptom Log$_i$ + Past Future Log$_i$	0	0	$\chi^2(1) = 0$, $p = 1$	No difference.

Robustness tests for regression models

First, we found compliance was inversely correlated with metacognitive bias (*rho* = -0.23, *p* = .007) – likely due to staircasing procedure causing accuracy to converge to ~70% the more the task is played. Hence, we repeated our logistic mixed effect model with metacognitive bias predicting a symptom log, but this time controlling for compliance (number of notifications answered throughout assessment period). Metacognitive bias remained a significant predictor of a future symptom log (Odds ratio = 0.76, β = -0.28, *p* = .023).

For regression analyses of interest, we assessed robustness by accounting for different slopes per subject, and controlling for age and gender. Given that the probability of occurrence of OCD symptom logs varied by current activities, we also reran models controlling for these variables. Moreover, as state OCD severity was affected by time-of-day, day-of-the-week, and current activities, we also controlled for these variables when assessing the impact of state variables (self-confidence and OCD severity ratings) on the occurrence of symptom logs. Following these robustness checks, we found that all findings outlined in the main text were maintained. Table S8 outlines these results.

Table S8: Results from robustness checks for models reported in paper. Two-sided tests were conducted.

Significant Model	Relevant Figure in Main Paper	Robustness Check	Significant result of interest
Current OCD Severity$_{,i}$ ~ Future Symptom Log$_i$ + Past Future Log$_i$	2e	Different slopes per subject	**Future Symptom Log:** β = 0.28, *p* < .001; **Past Symptom Log:** β = 0.18, *p* = .001
Current OCD Severity$_{,i}$ ~ Future Symptom Log$_i$ + Past Future Log$_i$	2e	Control for age and gender	**Future Symptom Log:** β = 0.24, *p* < .001; **Past Symptom Log:** β = 0.15, *p* < .001
Current OCD Severity$_{,i}$ ~ Future Symptom Log$_i$ + Past Future Log$_i$	2e	Control for time of day and day-of-week	**Future Symptom Log:** β = 0.23, *p* < .001; **Past Symptom Log:** β = 0.14, *p* < .001
Current OCD Severity$_{,i}$ ~ Future Symptom Log$_i$ + Past Future Log$_i$	2e	Control for current activities and social context	**Future Symptom Log:** β = 0.21, *p* < .001; **Past Symptom Log:** β = 0.14, *p* < .001
Future Symptom Log$_{i,t}$ ∈{0,1} ~ State Variables$_{t,i}$	3a	Different slopes per subject	**OCD severity:** β = 0.22, *p* = .19; **Self-Confidence:** β = -0.69, *p* < .001
Future Symptom Log$_{i,t}$ ∈{0,1} ~ State Variables$_{t,i}$	3a	Control for age and gender	**OCD severity:** β = 0.30, *p* = .007; **Self-Confidence:** β = -0.34, *p* = .005
Future Symptom Log$_{i,t}$ ∈{0,1} ~ State Variables$_{t,i}$	3a	Control for time of day and day-of-week	**OCD severity:** β = 0.03, *p* = .009; **Self-Confidence:** β = -0.03, *p* = .005
Future Symptom Log$_{i,t}$ ∈{0,1} ~ State Variables$_{t,i}$	3a	Control for current activities and social context	**OCD severity:** β = 0.27, *p* = .02; **Self-Confidence:** β = -0.34, *p* =.005
Current Self-Confidence$_{t,i}$ ~ Future Symptom Log$_i$ + Past Symptom Log$_i$	3b	Different slopes per subject	**Future Symptom Log:** β = -0.18, *p* < .001 Past Symptom Log: β = 0.0066, *p* = .88
Current Self-Confidence $_{t,i}$ ~ Future Symptom Log$_i$ + Past Symptom Log$_i$	3b	Control for age and gender	**Future Symptom Log:** β = -0.19, *p* < .001 Past Symptom Log: β = 0.003, *p* = .94
Current Self-Confidence $_{t,i}$ ~ Future Symptom Log$_i$ + Past Symptom Log$_i$	3b	Control for time of day and day-of-week	**Future Symptom Log:** β = -0.17, *p* < .001 Past Symptom Log: β = 0.003, *p* = .93
Current Self-Confidence $_{t,i}$ ~ Future Symptom Log$_i$ + Past Symptom Log$_i$	3b	Control for current activities and social context	**Future Symptom Log:** β = -0.18, *p* < .001 Past Symptom Log: β = -0.006, *p* = .88
Task metacognitive bias$_{t,i}$ ~ Current Self-Confidence $_{t,i}$	4b	Different slopes per subject	β = 0.12, *p* = .002
Task metacognitive bias$_{t,i}$ ~ Current Self-Confidence $_{t,i}$	4b	Control for age and gender	β = 0.11, *p* < .001
Task metacognitive bias$_{t,i}$ ~ Current Self-Confidence $_{t,i}$	4b	Control for time of day and day-of-week	β = 0.12, *p* < .001
Task metacognitive bias$_{t,i}$ ~ Current Self-Confidence $_{t,i}$	4b	Control for current activities and social context	β = 0.11, *p* < .001
Future Symptom Log$_{i,t}$ ∈{0,1} ~ Task Metacognitive Bias$_{t,i}$	4c	Different slopes per subject	β = -0.23, *p* = .038
Future Symptom Log$_{i,t}$ ∈{0,1} ~ Task Metacognitive Bias$_{t,i}$	4c	Control for age and gender	β = -0.21, *p* = .035
Task Metacognitive Bias$_{t,i}$ ~ Future Symptom Log$_i$ + Past Symptom Log$_i$	4d	Different slopes per subject	**Future Symptom Log:** β = -0.21, *p* = .034 Past Symptom Log: β = 0.027, *p* = .77
Task Metacognitive Bias$_{t,i}$ ~ Future Symptom Log$_i$ + Past Symptom Log$_i$	4d	Control for age and gender	**Future Symptom Log:** β = -0.21, *p* = .035 Past Symptom Log: β = 0.031, *p* = .71

Testing symptom log effects in shorter time windows

All symptom log results reported in the main paper define a 'symptom log occurrence' as a log conducted within a 4-hour time window from the completion of a state or task notification. Hence, we also confirmed that the results hold in shorter time windows (Table S9).

Table S9: Results from testing symptom log effects in shorter time windows. Two-sided tests were conducted.

Model	Time Window	Result
Symptom $Log_{i,t} \in \{0,1\}$ ~ Self-$confidence_{t,i}$	3 hours	$\beta = -0.23$, $p < .001$
Symptom $Log_{i,t} \in \{0,1\}$ ~ Self-$confidence_{t,i}$	2 hours	$\beta = -0.22$, $p < .001$
Symptom $Log_{i,t} \in \{0,1\}$ ~ Self-$confidence_{t,i}$	1 hour	$\beta = -0.29$, $p < .001$
Symptom $Log_{i,t} \in \{0,1\}$ ~ Task Metacognitive $Bias_{t,i}$	3 hours	$\beta = -0.26$, $p = .046$
Symptom $Log_{i,t} \in \{0,1\}$ ~ Task Metacognitive $Bias_{t,i}$	2 hours	$\beta = -0.36$, $p = .011$
Symptom $Log_{i,t} \in \{0,1\}$ ~ Task Metacognitive $Bias_{t,i}$	1 hour	$\beta = -0.31$, $p = .048$

Supplementary Note 5

Assessing reliability of task metacognitive bias

As metacognitive bias emerged as the strongest task predictor of symptom logs, we checked the reliability of this measure by calculating the intraclass correlation coefficient between each pair of games (e.g., 1 and 2, 2 and 8 etc) and computed the average per pair across all subjects (Figure S12). Average reliability for metacognitive bias was high (M = 0.83, standard error mean = 0.01, 95% CI [0.81, 0.85]).

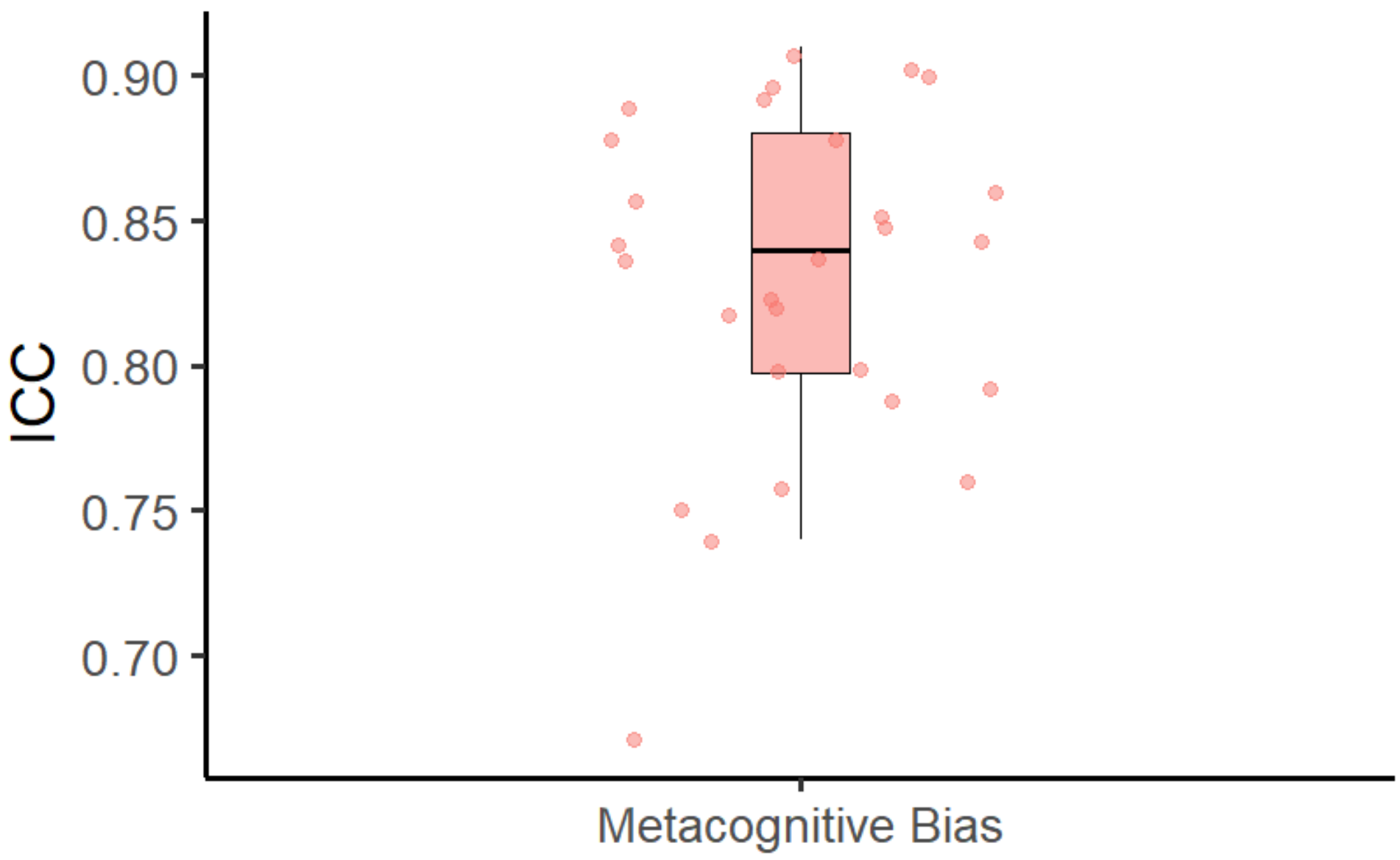

Figure S10: Average intraclass correlation coefficient for metacognitive bias. Each point is a pair of games (e.g., 1 and 2, 2 and 8 etc). N = 137.

Supplementary References

1. Mancebo, M. C. *et al.* Correlates of occupational disability in a clinical sample of obsessive-compulsive disorder. *Compr. Psychiatry* **49**, 43–50 (2008).

2. Markarian, Y. *et al.* Multiple pathways to functional impairment in obsessive-compulsive disorder. *Clin. Psychol. Rev.* **30**, 78–88 (2010).

3. Nota, J. A., Gibb, B. E. & Coles, M. E. Obsessions and time of day: A Self-Monitoring Study in Individuals With Obsessive-Compulsive Disorder. *J. Cogn. Psychother.* **28**, 134–144 (2014).
