# Supplementary material for: Drops in confidence precede symptoms of obsessive-compulsive disorder
Source: Nat Commun. 2026 Aug 13;17:8333. doi: 10.1038/s41467-026-76671-5 (PMC13473584; doi:10.1038/s41467-026-76671-5)
Supplement: Supplementary file 2 [file 41467_2026_76671_MOESM2_ESM.pdf]

nature portfolio

Corresponding author(s): Aleya Aziz Marzuki

Last updated by author(s): Oct 10, 2025

Reporting Summary

Nature Portfolio wishes to improve the reproducibility of the work that we publish. This form provides structure for consistency and transparency in reporting. For further information on Nature Portfolio policies, see our Editorial Policies and the Editorial Policy Checklist.

Statistics

For all statistical analyses, confirm that the following items are present in the figure legend, table legend, main text, or Methods section.

n/a	Confirmed	
☐	☒	The exact sample size (*n*) for each experimental group/condition, given as a discrete number and unit of measurement
☐	☒	A statement on whether measurements were taken from distinct samples or whether the same sample was measured repeatedly
☐	☒	The statistical test(s) used AND whether they are one- or two-sided<br>*Only common tests should be described solely by name; describe more complex techniques in the Methods section.*
☐	☒	A description of all covariates tested
☐	☒	A description of any assumptions or corrections, such as tests of normality and adjustment for multiple comparisons
☐	☒	A full description of the statistical parameters including central tendency (e.g. means) or other basic estimates (e.g. regression coefficient) AND variation (e.g. standard deviation) or associated estimates of uncertainty (e.g. confidence intervals)
☐	☒	For null hypothesis testing, the test statistic (e.g. *F*, *t*, *r*) with confidence intervals, effect sizes, degrees of freedom and *P* value noted<br>*Give P values as exact values whenever suitable.*
☒	☐	For Bayesian analysis, information on the choice of priors and Markov chain Monte Carlo settings
☐	☒	For hierarchical and complex designs, identification of the appropriate level for tests and full reporting of outcomes
☐	☒	Estimates of effect sizes (e.g. Cohen's *d*, Pearson's *r*), indicating how they were calculated

Our web collection on statistics for biologists contains articles on many of the points above.

Software and code

Policy information about availability of computer code

Data collection	Ecological momentary assessment (EMA) data collection was conducted using the Brain Explorer App: https://brainexplorer.net/, while questionnaire responses at the beginning of the experiment was collected using Redcap: https://projectredcap.org/software/.
Data analysis	All code for data processing and analysis can be found here: https://github.com/aleyaamarzuki/EMCT-OCD ;or in the following DOI: https://doi.org/10.5281/zenodo.21158125. R version 4.3.3. and CPM version 0.21.4 was used.

For manuscripts utilizing custom algorithms or software that are central to the research but not yet described in published literature, software must be made available to editors and reviewers. We strongly encourage code deposition in a community repository (e.g. GitHub). See the Nature Portfolio guidelines for submitting code & software for further information.

Data

Policy information about availability of data

All manuscripts must include a data availability statement. This statement should provide the following information, where applicable:

- Accession codes, unique identifiers, or web links for publicly available datasets
- A description of any restrictions on data availability
- For clinical datasets or third party data, please ensure that the statement adheres to our policy

All deidentified data generated in this study are deposited in an Open Science Framework database here: https://osf.io/x7h5k/. This includes trait questionnaire data, ecological momentary assessment data, and cognitive task data.

Research involving human participants, their data, or biological material

Policy information about studies with human participants or human data. See also policy information about sex, gender (identity/presentation), and sexual orientation and race, ethnicity and racism.

Reporting on sex and gender	We collected gender data (self-reported); participants chose between female, male, non-binary, or other. We conducted our analyses controlling for gender to assess whether gender explains any variability in the models.
Reporting on race, ethnicity, or other socially relevant groupings	Participants self-reported their ethnic background from a list of options: Arab, Asian, Black/African/Carribean/, Latin/Hispanic, White/Caucasian, Other, Unknown. This was not used as a covariate given the lack of variability and no prior assumptions about how ethnicity would impact OCD symptoms.
Population characteristics	We collected age and diagnosis data from participants.
Recruitment	Participants were recruited via Prolific (an online study recruitment platform), we screened for participants who reported that they were diagnosed with OCD and scored above a threshold for high OCD symptoms.
Ethics oversight	The study was approved by the University College London Research Ethics Committee (reference: 23505/001) and all participants provided informed consent.

Note that full information on the approval of the study protocol must also be provided in the manuscript.

Field-specific reporting

Please select the one below that is the best fit for your research. If you are not sure, read the appropriate sections before making your selection.

☐ Life sciences ☒ Behavioural & social sciences ☐ Ecological, evolutionary & environmental sciences

For a reference copy of the document with all sections, see nature.com/documents/nr-reporting-summary-flat.pdf

Behavioural & social sciences study design

All studies must disclose on these points even when the disclosure is negative.

Study description	Quantitative, micro-longitudinal design.
Research sample	137 participants dwelling in the United Kingdom with self-declared OCD diagnosis and whom scored above threshold on an OCD symptom scale (76.6% female, 21.2% male, 2.2% non-binary; Age: mean [M] = 32.8 years ± 8.22 [standard deviation]). Our ethics covered only participants from the UK. The sample is representative for a UK-based sample of people with OCD.
Sampling strategy	We conducted convenience sampling (based on participants whom signed up and fulfilled criteria on Prolific). The initial sample size pre-screened was based on samples sizes in prior EMA microlongitudinal work: https://www.pnas.org/doi/abs/10.1073/pnas.2417964122<br><br>There may have been biases in who had opted to participate. Participants with severe symptoms may have decided against joining the study due to the time commitment, which may have biased our results to being only representative of people with less severe functional impairments as a result of their OCD.
Data collection	Data collection was conducted via Redcap (online questionnaire platform) and the Brain Explorer app, wherein participants completed tasks as they received notifications to do so via the app on their mobile phones. Participants completed the tasks as they went about their daily routines, without an experimenter present. As there were no grouping conditions, blinding was not conducted.
Timing	Data was collected between 27th November 2024 and 5th February 2025
Data exclusions	We pre-screened 290 participants (residing in the United Kingdom) who declared they had a diagnosis of OCD on Prolific. Other inclusion criteria at the pre-screening stage were as follows: aged 18-55 years, fluency in English, and smartphone ownership. During pre-screening, we selected participants who scored ≥ 21 on the OCI-R (the traditional cutoff for OCD), and who did not have a history of neurological conditions or a diagnosis of Schizophrenia/psychosis. Following pre-screening, 286 participants were invited to the baseline assessment, which involved completing trait questionnaires and being briefed on the EMCT portion of the study; 201 participants completed this stage and were enrolled onto the EMCT part. After removal of dropouts and people who did not meet the completion criterion (minimum 50% EMCT notification completion), we analysed data from 137 participants.
Non-participation	Out of 286 participants that passed pre-screening, 85 did not proceed with the actual study. No reason was given by participants.
Randomization	Participants were not randomly allocated to any groups.

Reporting for specific materials, systems and methods

We require information from authors about some types of materials, experimental systems and methods used in many studies. Here, indicate whether each material, system or method listed is relevant to your study. If you are not sure if a list item applies to your research, read the appropriate section before selecting a response.

Materials & experimental systems

n/a	Involved in the study
☒	☐ Antibodies
☒	☐ Eukaryotic cell lines
☒	☐ Palaeontology and archaeology
☒	☐ Animals and other organisms
☒	☐ Clinical data
☒	☐ Dual use research of concern
☒	☐ Plants

Methods

n/a	Involved in the study
☒	☐ ChIP-seq
☒	☐ Flow cytometry
☒	☐ MRI-based neuroimaging

Plants

Seed stocks	*Report on the source of all seed stocks or other plant material used. If applicable, state the seed stock centre and catalogue number. If plant specimens were collected from the field, describe the collection location, date and sampling procedures.*
Novel plant genotypes	*Describe the methods by which all novel plant genotypes were produced. This includes those generated by transgenic approaches, gene editing, chemical/radiation-based mutagenesis and hybridization. For transgenic lines, describe the transformation method, the number of independent lines analyzed and the generation upon which experiments were performed. For gene-edited lines, describe the editor used, the endogenous sequence targeted for editing, the targeting guide RNA sequence (if applicable) and how the editor was applied.*
Authentication	*Describe any authentication procedures for each seed stock used or novel genotype generated. Describe any experiments used to assess the effect of a mutation and, where applicable, how potential secondary effects (e.g. second site T-DNA insertions, mosiacism, off-target gene editing) were examined.*
