# Supplementary material for: Drops in confidence precede symptoms of obsessive-compulsive disorder
Source: Nat Commun. 2026 Aug 13;17:8333. doi: 10.1038/s41467-026-76671-5 (PMC13473584; doi:10.1038/s41467-026-76671-5)
Supplement: Supplementary file 3 [file 41467_2026_76671_MOESM3_ESM.pdf]

nature portfolio

Peer Review File

Drops in confidence precede symptoms of OCD

Corresponding Author: Dr Aleya Marzuki

This file contains all reviewer reports in order by version, followed by all author rebuttals in order by version.

Version 0:

Reviewer comments:

Reviewer #1

(Remarks to the Author)
In this study, Marzuki and colleagues employ an ecological momentary assessment approach, to sample cognitive variables and subjective feelings of human participants with obsessive compulsive disorder throughout a period of two weeks.

The paper is well written, and clear, although a lot of the evidence is buried into the supplement – I appreciate that the journal format is short, but I would suggest to put back some of the findings into the main text so that readers do not have to believe you at your word.
I found the study findings very interesting and I think they add a valuable contribution to a growing literature in computational psychiatry addressing the relationships between subjective symptoms and cognitive task-based measures.

The robustness section should comment on what findings are maintained and what are not, besides Table S5, especially for the control by age and gender which is critical.
Currently the reader is left out to search for comparisons with the main text.
In addition, the results corresponding to current Fig 3b and 3E should also report the statistical test for past symptom log, not only future symptom log, to test whether a dissociation is still present – this is the central claim of the paper so it is crucial to evaluate if it remains true when controlling for age and gender. (especially since the sample happened to have more women)

In the supplement, the partial correlations only control for one variable at a time. Could the authors also test co-controlling for all variables at the same time in a single regression model, especially as they show in the correlation matrix just above that there are significant correlations (as expected)?

I could not find any information about medication so I assume the authors have not collected it. If so, this should be acknowledged as a limitation. If yes, does it affect the main result of shifts in metacognition preceding shifts in symptoms?

Since self-confidence rating results present a virtually identical pattern to metacognitive bias, I was curious about the authors' view on the use of a (meta)cognitive task for such predictions (just-in-time interventions). Even though task-based measures are useful for understanding (meta)cognitive distortions in OCD, for predictions of windows for intervention, do the authors agree that their findings indicate it is more efficient to ask participants for a single measure of self-report? (https://pmc.ncbi.nlm.nih.gov/articles/PMC8813275/)

The authors argue their findings will help design just-in-time support: what would such support look like? Bits of CBT? Interventions are notably difficult to produce generalisable effects; if the intervention is to talk with a therapist, then the scalable argument is difficult. What do the authors have in mind? (Even if I am not convinced by the just-in-time intervention argument, I reiterate that this does not undermine the basic research results which are valuable for the field.)

The Discussion paragraph on different modalities (or rather, measurements) should be rephrased as there is several overclaims. The authors indicate that their findings point to metacognition being domain-general, but no other domain than perception has been studied. Moreover, the momentary sample of confidence ('right now') is taken to mean a trait-level variable (« self-confidence ») i.e., pertaining to a unified self, which is problematic. Finally, the authors claim that they 'demonstrate how self-beliefs generalise across different domains', but a single domain is tested, perception, plus the

unique (self/though momentary) confidence rating (which seems to me does not constitute a 'domain' in and of itself).

Minor points

Suggestion for the main day-of-the-week analysis: code Monday-Friday vs. weekend? It seems unsurprising that the current coding by each of the seven days alone yields no clear pattern, and different participants may have different schedules.

I was also curious as to why the study focused on two weeks, since for variables like mood, they have been shown to vary over longer timescales too.

Table S5 refers to Figure 4 but there is no Fig 4; please fix the correct labels.

I would rephrase the title of Fig3b and 3e that mislead to think both past and future logs are significant

(Remarks on code availability)

Reviewer #2

(Remarks to the Author)
The manuscript presents an interesting attempt to link EMA-derived state measures of self-confidence and metacognitive performance with fluctuations in obsessive-compulsive (OCD) symptoms. The study has clear potential to contribute to our understanding of how metacognition dynamically relates to OCD symptom expression. However, several key methodological and reporting issues limit the interpretability and credibility of the results.

Uneven data distribution and missing participant-level reporting

The number of OCD symptom events is highly skewed across participants (median = 6), suggesting that the overall results are likely driven by a small subset of individuals who reported symptoms frequently. The absence of sensitivity analyses to assess this imbalance is a major concern. Sixteen participants, for example, logged only one symptom across the 14-day period, while a few high-frequency individuals appear to dominate the dataset. There is one participant who reported as many as 175 symptom logs. This raises the question of whether the reported effects are representative.

The authors should report per-participant data and demonstrate that the findings remain robust when high-frequency participants are excluded. We also note when looking at the data ourselves, that participants appeared to log primarily during the first few days, with a substantial decline in later entries. This dropout pattern further contributes to missingness. Additionally, the decision to retain only the first symptom log when multiple logs were recorded in close timing may distort the temporal signal. It is unclear how this choice affects the interpretation of momentary symptom fluctuations, especially if participants tended to log multiple events retrospectively (several at the end of a day).

Including a random intercept for each subject is essential to account for within-person dependence. The authors should report whether random effects were significant. At present, the absence of this information makes it difficult to assess model validity. A more detailed description of study compliance (response rates over time, dropout, timing of task completion) would also clarify data quality. The reported 50% completion threshold for inclusion may be too low, adopting a stricter cutoff could reduce variability and improve reliability.

Misplaced focus of sensitivity analyses

While the manuscript includes several sensitivity analyses (e.g., time-of-day and day-of-week effects), these appear overly detailed and somewhat tangential to the central questions of data robustness. They give the impression of a distraction from the more critical concerns, namely, the sparse and uneven distribution of OCD symptom events, the dominance of a few high-frequency participants, and the substantial amount of missing data.

Sensitivity analyses should instead directly test whether the reported effects persist under alternative models, smaller/completer samples or resampling procedures that explicitly address these imbalances and missingness.

Insufficient model specification and lack of clarity on random effects

The description of the statistical models in the main text is incomplete (and also not clear in the supplementals data). It is unclear which predictors and outcome variables were included, whether random effects for subjects were modelled, and how within-person correlations were handled. Given the repeated-measures design and the large heterogeneity in event frequency, inclusion of random subject effects is essential. The full model specifications, estimation methods, and justification for the chosen model structure should be reported in detail in the main text.

Furthermore, Equation 3 in the Methods section appears to be mis-specified. If the goal is to test how self-confidence influences past and future symptom logs, then the dependent variable should be the symptom log, not self-confidence. In other words, the equation should be reversed. Without this correction, the analysis instead tests how symptoms predict self-confidence, not vice versa.

Finally, the section around line 636, which mentions a 'fitting procedure' is unclear: it is not specified what model was fitted or how this step relates to the primary analyses.

Inadequate reporting of task-level data and metacognitive measures

The calculation of metacognitive bias appears incorrect. The manuscript treats it as the mean of the confidence ratings, but conventionally, metacognitive bias (or calibration) is calculated as mean accuracy minus mean confidence. The authors should clarify this computation and revise terminology accordingly. As currently analyzed, the measure represents simple confidence in decisions, not bias.

Task performance data, including mean accuracy, confidence, and derived measures, should be reported at the individual-participant level with measures of variability (e.g., SDs or CIs). This would allow readers to evaluate individual differences and data reliability.

Figure C, showing within-subject correlations between self-confidence and metacognitive bias, reveals very high variability, including a subset of participants showing negative correlations. These negative associations should be explicitly reported and discussed rather than implied visually.
For transparency, the authors should also specify whether participants excluded from meta-d′ analyses (due to insufficient trials) were also excluded from other analyses, or whether the sample size varies across reported effects.

Staircasing procedure and task data

The authors state that a staircase procedure was used to maintain ~70% accuracy across participants, but they do not present evidence that this was achieved. The mean accuracy and its standard deviation should be reported, as should verification that performance remained stable over time. Given that only 20 trials were used for the staircase, this may have been insufficient to converge on a reliable threshold. The lack of proof that the staircasing worked properly undermines claims that differences in confidence reflect metacognitive rather than performance.

Interpretation of temporal effects

The reported finding that future OCD symptom events predict task confidence better than past events is not particularly surprising, given that lag/lead analyses inherently favor forward prediction and struggle to interpret retroactive effects. The text around line 259 is also slightly confusing: “the other way around” refers to past symptoms, and this should be clarified for precision.

Broader interpretational issues

In the discussion, the authors refer to the ongoing debate in the field regarding differences between clinical OCD populations and transdiagnostic samples of highly compulsive individuals. Previous work has shown that clinically diagnosed OCD patients typically display reduced confidence, whereas transdiagnostic or non-clinical compulsive samples sometimes show elevated confidence levels. However, it remains unclear what this study contributes to that debate. The authors use self-identified OCD participants who also exhibit low confidence, replicating earlier findings rather than clarifying the discrepancy between clinical and transdiagnostic results. Stronger theoretical arguments are needed to explain how these findings advance understanding of the confidence–compulsivity relationship across diagnostic boundaries.

Additional procedural and compliance questions

Several practical aspects of the EMA design remain unclear:

• What happened if participants received the notification for the task during a busy moment, could they postpone it?
• Was there any mechanism to verify adherence to timing instructions?
• Were participants allowed to indicate explicitly that they experienced no symptoms during the day?

Clarifying these procedures would strengthen the interpretation of compliance and data validity.

Summary

In summary, while the study addresses an important and innovative question, it currently lacks sufficient methodological transparency and robustness. To strengthen the manuscript, the authors should:

1. Provide complete model specifications, including fixed and random effects.
2. Enhance transparency regarding study compliance, exclusions, and timing procedures.
3. Report participant-level data and perform sensitivity analyses that address uneven event distributions, dropout, and missingness.
4. Present clear validation of the task and staircase procedure, including accuracy metrics and convergence evidence.
5. Correct the metacognitive bias calculation and terminology, and report variability in within-subject correlations.
6. Clarify Equation 3 and ensure the direction of temporal prediction aligns with the stated hypotheses.

Only with these revisions can the reported associations be interpreted with confidence.

I co-reviewed this manuscript with one of the reviewers who provided the listed reports. This is part of the Nature Communications initiative to facilitate training in peer review and to provide appropriate recognition for Early Career Researchers who co-review manuscripts.

(Remarks on code availability)

There are also several concerns related to data reproducibility and the structure of the dataset itself.
The code is generally well-documented, with a README file that clearly explains how to install dependencies and run the analyses. However, the Notif_times.csv file, which appears to be required to reproduce the analyses from the provided R script, is missing. Without this file, most of the reported analyses cannot be replicated, limiting transparency and verification of the results.

We also inspected the dataset and observed that the data distribution is highly skewed: almost all participants reported symptoms on the first day, but by day 14 only about 10% continued logging. Sixteen participants reported only a single symptom over the 14-day period, while one participant logged as many as 175. This imbalance raises concerns about the representativeness of the results and the influence of high-frequency reporters. Notably, participants completed daily questionnaires consistently, suggesting that the decline is specific to symptom logging rather than general engagement.

Finally, aside from the issue with Equation 3 (which should be reversed to test directional effects properly), the main analyses appear appropriate. Nevertheless, the analysis script includes many side analyses not directly related to the primary research question, which may dilute the focus and could be moved to supplementary materials for clarity.

Reviewer #3

(Remarks to the Author)
I co-reviewed this manuscript with one of the reviewers who provided the listed reports. This is part of the Nature Communications initiative to facilitate training in peer review and to provide appropriate recognition for Early Career Researchers who co-review manuscripts.

(Remarks on code availability)

Version 1:

Reviewer comments:

Reviewer #1

(Remarks to the Author)
I have now carefully read the rebuttal and the authors' additional analyses have addressed all my previous comments. I think the extra control analyses provide important nuance to the conclusions of the study for the readers.

(Remarks on code availability)

Reviewer #2

(Remarks to the Author)
We thank the authors for their careful and thorough revisions and for the effort they made to address the other reviewer’s and our comments. We appreciate the additional analyses and clarifications that were added to the manuscript, as well as the detailed responses provided. Overall, these revisions have improved the manuscript and helped clarify several aspects of the analyses. That said, we still have a few remaining points that we would like the authors to address or clarify further.
Uneven data distribution and participant-level reporting
We thank the authors for their response to this comment and for conducting additional sensitivity analyses to address the concerns regarding uneven data distribution and potential missingness. We appreciate the effort to replicate the main analyses after removing the highest-frequency logger and participants who logged only once, as well as the inclusion of the split-half analyses examining Week 1 versus Week 2. These additions improve the transparency of the analyses and help clarify the robustness of the reported effects.
However, we remain somewhat concerned that the issue of data skewness may not yet be fully addressed. While removing the single highest-frequency logger (175 logs) and participants who logged only once is a helpful first step, the distribution of symptom logs still appears highly uneven. As illustrated in Figure S1B, there remain several participants who logged only two symptoms, while others logged very large numbers (e.g., above 100). Consequently, the dataset may still be disproportionately influenced by a relatively small number of high-frequency reporters. We therefore encourage the authors to further discuss this imbalance and consider whether additional robustness checks might help demonstrate that the findings are not driven by this remaining skew.
Relatedly, because the distribution of symptom logging across participants is central to evaluating the robustness of the results, we suggest that the figure illustrating this distribution (currently Figure S1B in the Supplementary) may be better placed in the main manuscript rather than the Supplementary. Presenting this information more prominently would improve transparency and allow readers to more easily assess the structure of the dataset.
Regarding the split-half analyses, we agree that examining Week 1 and Week 2 separately is a useful approach given the decline in symptom logging over time. However, we find the interpretation that the disappearance of effects in Week 2 is “likely due to limited power rather than a true absence of effect” somewhat strong. While reduced sample size and data density could certainly contribute to the lack of statistical significance, the current analyses do not allow one to clearly distinguish between insufficient power and a genuine weakening or absence of the effect later in the study. We therefore suggest that the authors frame this interpretation more cautiously in the manuscript.
Study compliance and completion threshold
We thank the authors for their response and for adding additional analyses and visualisations addressing study compliance. In particular, we appreciate the inclusion of the plot showing the number of participants logging symptoms over time, as well as the additional analysis using a stricter 75% completion threshold. These additions help clarify aspects of participant engagement and provide useful robustness checks.
However, we remain somewhat uncertain about how “completion” is defined in this context. From the description provided, it appears that completion refers primarily to whether participants responded to the prompted task notifications. However, an important component of the study design is the self-directed symptom logging. The current analyses therefore leave some ambiguity as to whether participants who met the completion threshold also continued to log symptoms throughout the study period, or whether some participants may have continued completing the prompted tasks while no longer reporting symptoms. Given that symptom logging itself is a central outcome variable in the analyses, this distinction is important for interpreting compliance and data quality. It would therefore be helpful if the authors could clarify how completion was operationalised and whether symptom logging behaviour was considered as part of the completion criteria.
Additionally, it is not entirely clear whether the analyses using the stricter 75% completion threshold were conducted on the full dataset or on the dataset after excluding the extreme loggers discussed in the previous point. Clarifying this would improve transparency. If these analyses were conducted on the full dataset, we would encourage the authors to also consider applying the stricter completion threshold in combination with the exclusion of extreme loggers, as this may provide a more informative assessment of the robustness of the findings.

Reporting of task-level data and metacognitive measures
We thank the authors for their clarification regarding the operationalisation of metacognitive bias and for explaining how their definition aligns with the cognitive neuroscience literature. While we understand that the staircase procedure helps to ensure stable performance levels and can therefore be informative when assessing potential confidence biases (i.e., systematic deviations between subjective confidence and objective performance), it is important to note that confidence reports themselves do not constitute a bias. A bias implies a systematic misalignment between confidence and actual performance. For example, if participants report an average confidence of 70% and their performance is also around 70%, this would indicate good calibration rather than a bias.

In the current manuscript, the term “confidence bias” appears to be used in places where the authors are in fact referring to confidence reports. Strictly speaking, this is conceptually inaccurate unless the analysis demonstrates a deviation between confidence and performance. To make this interpretation correct, the authors would need to explicitly quantify and report the relationship between confidence and accuracy.
Additionally, if a bias is present, it would be important to specify its direction, as confidence biases can reflect either overconfidence or underconfidence. Based on the description of the results, the pattern seems more consistent with underconfidence rather than overconfidence. At minimum, the manuscript should clarify this distinction in the Method section and briefly indicate the direction of the bias.
Minor points
• There appear to be some inconsistencies in the numbering of the supplementary figures. The manuscript refers to Figure S11A in the Supplementary Materials, but in the document this figure appears to be labeled Figure S4, while another figure is already labeled S4. Similarly, there appear to be two figures labeled S3. We recommend carefully checking the numbering of all supplementary figures to ensure consistency with the references in the text.
• Additionally, the figure illustrating the decline in symptom logging over time (referred to as Figure S11A, currently labeled S4A) is quite central to the discussion of compliance and missingness. The authors may want to consider whether this figure would be more appropriate in the main text rather than the Supplementary Materials, as it helps contextualize several of the sensitivity analyses discussed in the manuscript.

Regarding scripts and data-analyse:
We appreciate that the authors have now uploaded the previously missing Notif_times.csv file and provided an updated analysis script including the additional analyses conducted during the revision. With the inclusion of this file, the analyses can now be reproduced, improving transparency and reproducibility.

I co-reviewed this manuscript with one of the reviewers who provided the listed reports. This is part of the Nature Communications initiative to facilitate training in peer review and to provide appropriate recognition for Early Career Researchers who co-review manuscripts.

(Remarks on code availability)
Regarding scripts and data-analyse:
We appreciate that the authors have now uploaded the previously missing Notif_times.csv file and provided an updated analysis script including the additional analyses conducted during the revision. With the inclusion of this file, the analyses can now be reproduced, improving transparency and reproducibility.

Reviewer #3

(Remarks to the Author)
I co-reviewed this manuscript with one of the reviewers who provided the listed reports. This is part of the Nature Communications initiative to facilitate training in peer review and to provide appropriate recognition for Early Career Researchers who co-review manuscripts.

(Remarks on code availability)

Version 2:

Reviewer comments:

Reviewer #2

(Remarks to the Author)
We have reviewed the revised manuscript and are satisfied that the concerns have been adequately addressed, and we therefore recommend approval of the paper.

(Remarks on code availability)
Is complete and readable

Reviewer #3

(Remarks to the Author)
I co-reviewed this manuscript with one of the reviewers who provided the listed reports. This is part of the Nature Communications initiative to facilitate training in peer review and to provide appropriate recognition for Early Career Researchers who co-review manuscripts.

(Remarks on code availability)
Everything is good.

Open Access This Peer Review File is licensed under a Creative Commons Attribution 4.0 International License, which permits use, sharing, adaptation, distribution and reproduction in any medium or format, as long as you give appropriate credit to the original author(s) and the source, provide a link to the Creative Commons license, and indicate if changes were made.
In cases where reviewers are anonymous, credit should be given to 'Anonymous Referee' and the source.
The images or other third party material in this Peer Review File are included in the article's Creative Commons license, unless indicated otherwise in a credit line to the material. If material is not included in the article's Creative Commons license and your intended use is not permitted by statutory regulation or exceeds the permitted use, you will need to obtain permission directly from the copyright holder.
To view a copy of this license, visit https://creativecommons.org/licenses/by/4.0/

Dear reviewers,

In this rebuttal, we have marked the reviewer's comments in **bold**, our responses are in standard text, and direct quotations from the manuscript in blue. All page numbers and line number refer to the marked-up main manuscript with changes in red, unless stated otherwise (i.e., they may refer to the Supplementary). We also include some responses to the editorial comments below.

START OF RESPONSE

Reviewer #1 (Remarks to the Author):

In this study, Marzuki and colleagues employ an ecological momentary assessment approach, to sample cognitive variables and subjective feelings of human participants with obsessive compulsive disorder throughout a period of two weeks.

1.1 The paper is well written, and clear, although a lot of the evidence is buried into the supplement – I appreciate that the journal format is short, but I would suggest to put back some of the findings into the main text so that readers do not have to believe you at your word.

I found the study findings very interesting and I think they add a valuable contribution to a growing literature in computational psychiatry addressing the relationships between subjective symptoms and cognitive task-based measures.

We would like to thank Reviewer #1 for their very positive assessment of our manuscript, the encouraging remarks and the many helpful suggestions. We have now addressed all raised points and provide detailed answers in the responses below. Moreover, we have re-worked the result section and tried to add further information from the supplement to

the main manuscript so that readers can evaluate the findings without a deep dive in the supplement.

Among them, we have included results in the main manuscript for weekday and context effects for the symptom log:

"Additionally, we confirmed that symptom logs were meaningfully capturing external events (i.e., whether probability of a symptom log varied based on current activities and social contexts) and explored how symptom log occurrence fluctuated by the time-of-day and the day-of-week. Briefly, we uncovered that OCD symptom logging was, on average, more frequent on weekdays (per day mean = 2.34 ± 3.73) compared to weekends (per day mean = 1.95 ± 3.38), Wilcoxon's V = 4506, p = .008, and that partaking in restful (Odds ratio = 0.59 =, 13 = -0.54, p < .001), and self-care (Odds ratio = 0.69, 13 = -0.37, p = .008) activities promoted lowered likelihood of symptom logging. This suggests, intuitively, engaging in relaxing or enjoyable activities is associated with reduced OCD symptoms.." – Page 7 Lines 188 - 197

Furthermore, we have added statistics for our validation checks into whether the symptom log results hold when evaluating different time windows between survey completion and symptom log:

For self-confidence – "These results held even when testing shorter time windows between symptom logs and state questionnaires (i.e., within 3 hours: 13 = -0.23, p < .001; 2 hours: 13 = -0.23, p < .001; and 1 hour: 13 = -0.29, p < .001)." – Page 9 Lines 248 - 249

For metacognitive bias – "Finally, using shorter time windows between game completion and symptom log (<4 hours) yielded similarly significant results (3 hours: 13 = -0.26, p = .046; 2 hours: 13 = -0.36, p = .011; 1 hour: 13 = -0.31, p = .048)." – Page 12 Lines 332 - 334

1.2 The robustness section should comment on what findings are maintained and what are not, besides Table S5, especially for the control by age and gender which is critical. Currently the reader is left out to search for comparisons with the main text.

We apologise for this lack of clarity and have now made it clearer in text that all findings described in the main text were robust in the face of the checks we conducted:

"Following these robustness checks, we found that all findings outlined in the main text remained significant." – Supplementary Page 15 Lines 280-281

Additionally, in (now) Table S8 (Supplementary Pages 16-17) we have listed all the results of the robustness checks including those controlling for age and gender, and we have now updated the last column of the table to be titled '**Significant result of interest**' so that there is no ambiguity that our findings were maintained.

1.3 In addition, the results corresponding to current Fig 3b and 3E should also report the statistical test for past symptom log, not only future symptom log, to test whether a dissociation is still present – this is the central claim of the paper so it is crucial to evaluate if it remains true when controlling for age and gender. (especially since the sample happened to have more women)

We thank the reviewer for pointing this out. In our models, we entered both the past symptom log and the future symptom log, testing for the significance of both. We agree that it was not evident in the previous results version that the past symptom log was not significant. To remediate this, we reworked Figures 3 (page 10) and 4 (page 12) and explicitly add p-values (p< .001 or p < .05 if statistically significant and p > .05 if non-significant) to enhance comprehension of our findings. Regarding the past symptom log, this is explicitly included in the models depicted in Figures 3b and 4d.

Moreover, we have updated our figure captions such that it is now made explicit that future symptom logging is linked to both self-confidence and metacognitivie bias, but past symptom logging is unrelated to either:

Figure Caption 3b: "The effects of confidence on symptom logs are unidirectional; high self-confidence is associated with a lower probability of a symptom being logged, but occurrence of a past symptom log did not predict future self-confidence." – Page 10

Figure Caption 4d: "the effect of metacognitive bias over symptom logs is unidirectional; metacognitive bias significantly predicts future symptom logs but past symptom logs do not predict metacognitive bias." – Page 12

The statistical tests for past symptom logs are now reported in-text with clearer language used:

"We uncovered that the relationship between self-confidence and the likelihood of logging a symptom was unidirectional: in a linear mixed-effects model current self-confidence was significantly associated with a future symptom log (13 = -0.18, 95% CI [0.26 -0.10], p < .001; Figure 3b) but not with a past symptom log (13 = 0.007, 95% CI [-0.08 0.09], p = .88)." – Page 9 lines 254 - 258

"In line with our self-confidence findings, we saw that reduced metacognitive bias predicted future symptom logs (13 = -0.21, 95% CI [-0.40 -0.015], p = .035) but past symptom logs did not impact current metacognitive bias (13 = 0.03, 95% CI [-0.13 0.19], p = .69)." – Page 11-12 lines 336 - 339

Furthermore, the robustness tests outlined in the Supplementary in Table S8 now include statistical results for the past symptom log impacting self-confidence and meta-cognitive bias. Control analyses for age and gender are included showing that the results remain when controlling for these covariates (Supplementary, Page 16 - 17).

1.4 In the supplement, the partial correlations only control for one variable at a time. Could the authors also test co-controlling for all variables at the same time in a single regression model, especially as they show in the correlation matrix just above that there are significant correlations (as expected)?

We apologise for not having this made clearer in the original manuscript. The reason for running independent analyses is to establish an association. Critically, we reasoned for some of the measures (e.g., mood and anxiety) to be collinear, meaning we did not expect them to uniquely predict the outcomes.

However, we agree that there is merit in allowing these predictors to directly compete for variance. We have now added analyses where we controlled for all variables using linear models. We find that many findings remained significant after controlling for the other factors with some less critical effects no longer reaching significance (but maintaining the same directionality). We have now added the details on this in our revised manuscript.

"As an extra measure of robustness, we assessed whether relationships between state variables (OCD and confidence) and trait variables (Rosenburg Self-Esteem, CAIOC-13, and OCI-R) held when controlling for all other state variables (anxiety, sleep quality, brain fog, happiness) simultaneously (see Table S6) using linear models.

We found that the associations between averaged state confidence and CAIOC-13 scores (13 = -8.00, 95% CI [-13.5 -2.42], $p = .005$), and between averaged state OCD and OCI-R scores (OCD: 13 = 6.09, 95% CI [0.84 11.35], $p = .024$) were still significant even when controlling for all other state variables. However, while the direction of the relationship was maintained, the association between Rosenburg Self-Esteem scores and state averaged self-confidence only showed a trend-level significance (13 = 2.66, 95% CI [-0.06 5.38], $p = .055$). Nonetheless, no other state measure emerged as being more strongly associated with trait self-esteem (state OCD: 13 = 0.58, $p = .67$; state anxiety: 13 = -0.84, $p = .54$; state sleep quality: 13 = 1.03, $p = .26$; state brain fog: 13 = -0.26, $p = .833$, state happiness: 13 = 1.89, $p = .21$)." – Supplementary Page 8 Lines 94-107 (also see Table S6, page 8).

1.5 I could not find any information about medication so I assume the authors have not collected it. If so, this should be acknowledged as a limitation. If yes, does it affect the main result of shifts in metacognition preceding shifts in symptoms?

This reviewer is indeed correct in their assumption that we did not collect medication information, unfortunately. As suggested by the reviewer, we have now mentioned this as a limitation in our revised Discussion section.

"Lastly, it is important to highlight that we did not collect information on current medication intake and thus are unable to assess how OCD symptom fluctuations may have been affected by momentary psychiatric drug use." – Page 16 Lines 477 - 479

1.6 Since self-confidence rating results present a virtually identical pattern to metacognitive bias, I was curious about the authors' view on the use of a (meta)cognitive task for such predictions (just-in-time interventions). Even though task-based measures are useful for understanding (meta)cognitive distortions in OCD, for predictions of windows for intervention, do the authors agree that their findings indicate it is more efficient to ask participants for a single measure of self-report? (https://pmc.ncbi.nlm.nih.gov/articles/PMC8813275/)

We thank the reviewer for this highly relevant and thought-provoking query. The goal of our paper was to examine whether either or both would be predictive. Finding meaningful associations with both was quite striking for us and speaks to a broad drop in confidence, rather than a drop in a specific domain/modality. If the main purpose would simply be to predict the likelihood of symptoms, then we agree that a simple self-report would increase speed and efficiency of data collection.

However, to understand these fluctuations on a more mechanistic, and potentially even neural, level then including task-based measures would be valuable. For example, measuring task-based metacognition would allow the adoption of mobile neuroimaging techniques to identify neural mechanisms driving confidence fluctuations, as trial-by-trial data would enable more reliable neuroimaging estimates compared to a single self-confidence measure at a given time point.

As this is a relevant consideration, we have expanded our Discussion in the revised manuscript along these lines and now make reference to the cited paper by Bennett and colleagues (2019).

"As our findings indicate that *task* metacognitive biases drive moment-to-moment symptom emergence, this could serve as a foundation for future work to incorporate mobile neuroimaging techniques with EMCT to identify the neural mechanisms underlying these fluctuations. Whilst the neural mechanisms associated with *fluctuations* in metacognition remain unknown, we speculate that connectivity between prefrontal cortices and right dorsal anterior cingulate cortex and striatal regions could act as drivers given their involvement both in metacognition[1,2] and OCD symptomatology[3,4]. Nonetheless, if we just wanted to predict the likelihood of future symptom occurrence, a simple confidence self-report measure could suffice over a more time-consuming task-confidence paradigm[5]." - Page 15 Lines 446 - 455

1.7 The authors argue their findings will help design just-in-time support: what would such support look like? Bits of CBT? Interventions are notably difficult to produce generalisable effects; if the intervention is to talk with a therapist, then the scalable argument is difficult. What do the authors have in mind? (Even if I am not convinced by the just-in-time intervention argument, I reiterate that this does not undermine the basic research results which are valuable for the field.)

We thank the reviewer for challenging our speculative musings. Our reading of the literature indicates relatively few studies on just-in-time interventions but there are implications that delivering interventions at the right time might further improve their effectiveness [6]. As for the interventions for our case, one could devise delivering an intervention building on exposure & response prevention to directly impact the symptoms, or an intervention that would alter confidence (which may subsequently lower the likelihood of symptom occurrence). For the latter, task-based or psychotherapeutic interventions, such as metacognitive training may show effectiveness.

We have now expanded on our thoughts in the revised discussion, and highlight their speculative character, which we hope the reviewer will appreciate.

"While this line of research is still in its infancy, we speculate that task-based metacognitive interventions may prove promising, for instance by providing selective feedback on performance, as this has been found to improve both trial-by-trial and overall task confidence in prior work [7]. Just-in-time interventions could also build on existing metacognitive training programmes, shown to be effective for disorders like OCD and depression [8,9]. These trainings, possibly initially introduced by their therapist, could be triggered via smartphone notifications by reports of low confidence and symptom logging. Such an approach would be relatively cost-efficient and moderately scalable. Moreover, therapist access to momentary assessment reports could help improve therapeutic treatment outcomes – as they can provide rich context [10] into where and when low confidence and OCD symptoms could occur." – Page 16 Lines 466 - 476

1.8 The Discussion paragraph on different modalities (or rather, measurements) should be rephrased as there is several overclaims. The authors indicate that their findings point to metacognition being domain-general, but no other domain than perception has been studied. Moreover, the momentary sample of confidence ('right now') is taken to mean a trait-level variable (« self-confidence ») i.e., pertaining to a unified self, which is problematic. Finally, the authors claim that they 'demonstrate how self-beliefs generalise across different domains', but a single domain is tested, perception, plus the unique (self/though momentary) confidence rating (which seems to me does not constitute a 'domain' in and of itself).

We apologise if our discussion was misleading and we agree with the reviewer that no other task-domain was tested beyond visuo-perceptual metacognition. The main idea behind said discussion section was the striking alignment between task-based measures, simple momentary self-reported confidence, and – to some extent – trait measures of confidence.

In reference to the comment on domain generality, we would like to clarify that we theorise that our momentary self-confidence measure is potentially tapping into higher order 'global' (self-referential) metacognition which aligns with the metacognitive hierarchy framework conceptualised in the recent review paper by Seow et al. (2025). On the other hand, trial-by-trial confidence (i.e., our metacognitive bias measure) might potentially be tapping into local levels of confidence. We have made this clearer in the Discussion by explicitly (and tentatively) positing how our confidence measures fit into global and local metacognitive levels. Nevertheless, we concede that more work validating the momentary self-confidence measure and how it pertains to global self-image is needed.

Based on this reviewer's comment, we have now revised the entire section and clarify all these points in our revised text.

".. we acknowledge that the tested ratings (decision-making and self-confidence) remain limited in scope and future research should characterise how more diverse domains of confidence are interrelated, along the lines of previous research that has compared memory and perceptual confidence[11]." – Page 15 Lines 442 - 445

"Briefly, lower levels along the hierarchy are thought to pertain to 'local' metacognition (i.e., confidence in specific contexts such as trial-by-trial decisions on our cognitive task) and higher levels represent 'global' metacogniton related to metrics like confidence in oneself (i.e., what our self-confidence item taps into). In the context of the present study, we demonstrate how self-beliefs **perhaps** generalise across global and local metacognitive levels and that alterations in these beliefs go on to **potentially** drive obsessive-compulsive symptoms." – Page 15 Lines 424 - 431

"Next, while averaged moment-to-moment self-confidence was significantly correlated with trait-level self-esteem, more research needs to investigate whether this novel confidence measure truly is representative of one's global unified self-image. For instance, assessing whether long-term changes in self-esteem reflect shifts in momentary self-confidence." – Page 15 Lines 437 – 440

Minor points

1.9 Suggestion for the main day-of-the-week analysis: code Monday-Friday vs. weekend? It seems unsurprising that the current coding by each of the seven days

alone yields no clear pattern, and different participants may have different schedules.

We thank the reviewer for their suggestion, which we have now implemented. Indeed, when collapsing days of the week data into 'Weekdays' and 'Weekends', we found significant effects of increased symptom logging and OCD severity on weekdays compared to weekends. We have now provided details of this in the main Results (page lines), and the supplement (see Supplementary page 10-11 lines 157 – 161 & lines 178 – 183, and in Figure S6 (page 11)):

"Briefly, we uncovered that OCD symptom logging was, on average, more frequent on weekdays (per day mean = 2.34 ± 3.73) compared to weekends (per day mean = 1.95 ± 3.38), Wilcoxon's V = 4506, *p* = .008..." – Page 7 Lines 191 - 193

1.10 I was also curious as to why the study focused on two weeks, since for variables like mood, they have been shown to vary over longer timescales too.

Previous microlongitudinal work, which we had conducted over a two-week period has unveiled promising links between mood, motivation, and computational task parameters. Thus, we opted to continue this timeframe, while also increasing the frequency of notifications (x4 instead of x2) to better capture the temporality of OCD symptoms, which show fluctuations from morning to afternoon to evening (Nota et al., 2014).

While it may have been better to collect data over more weeks, we were also cognisant of the risk of increased dropout and missing data. Thus, two weeks with more frequent notifications seemed to be the best trade-off for obtaining enough data and avoiding excessive attrition. We now mention this reasoning in the revised methods section:

"This timeframe was chosen based on prior work demonstrating promising links between mood and computational task parameters over a 2-week period." – Page 18 Lines 530 - 532

"We programmed notifications to be sent out this frequently (x4 a day) to rigorously capture time-of-day effects on symptom severity – as OCD symptoms are reported to fluctuate significantly within a single day [12]." – Page 18 Lines 536 - 539

1.11 Table S5 refers to Figure 4 but there is no Fig 4; please fix the correct labels.

We thank the reviewer for pointing out this error which we have now rectified. In fact, following this review, we now have 4 figures, and we have ensured the labels are correct in Table S8 (formerly Table S5).

1.12 I would rephrase the title of Fig3b and 3e that mislead to think both past and future logs are significant

The title of the figures for 3b (page 10) and 4d (page 12) have now been changed to “Association between Self-confidence and Past-Future Symptom Log” and “Association between Metacognitive Bias and Past-Future Symptom Log” respectively.

<u>Reviewer #2 (Remarks to the Author):</u>

The manuscript presents an interesting attempt to link EMA-derived state measures of self-confidence and metacognitive performance with fluctuations in obsessive-compulsive (OCD) symptoms. The study has clear potential to contribute to our understanding of how metacognition dynamically relates to OCD symptom expression. However, several key methodological and reporting issues limit the interpretability and credibility of the results.

2.1 Uneven data distribution and missing participant-level reporting
The number of OCD symptom events is highly skewed across participants (median = 6), suggesting that the overall results are likely driven by a small subset of individuals who reported symptoms frequently. The absence of sensitivity analyses to assess this imbalance is a major concern. Sixteen participants, for example, logged only one symptom across the 14-day period, while a few high-frequency individuals appear to dominate the dataset. There is one participant who reported as many as 175 symptom logs. This raises the question of whether the reported effects are representative.
The authors should report per-participant data and demonstrate that the findings remain robust when high-frequency participants are excluded. We also note when looking at the data ourselves, that participants appeared to log primarily during the first few days, with a substantial decline in later entries. This dropout pattern further contributes to missingness.

We thank the reviewers for their generally positive assessment of our paper and its timely and novel contribution. We are also grateful for them raising the important issue of missing/uneven data. We agree that the imbalanced data could be impacting results, and based on the reviewers' suggestion, we have conducted two key sensitivity tests to address this.

First, we checked the robustness the results of our main analyses (namely the effects of state self-confidence and metacognitive bias on future symptom logging) by removing the high frequency logger (175 logs total) as well as infrequent loggers (those who logged only once). Critically, removing these subjects did not affect the results and we replicated the same findings with this reduced dataset (see below). We have now added these analyses to the revised manuscript.

Next, we thank the reviewers for sharing their observation that participants reported more symptoms in the first part of the study. Given the decline in logging in later days, we conducted a 'split-half' analysis where we divided data into Week 1 (days 1 - 7) and Week 2 (days 8 - 14). We then reran our main models within each week. Critically, even if we only look at the first half of the study time, we replicate the findings (note that one effect

drops to p=.051, which is to be expected as we lose power). This means that the effect cannot be explained by time effects of a drop in symptom reports with time alone, which shows the robustness of the effect. Please note that the effects in the second week, where we indeed have significantly fewer data points, are less clear (see below). We now mention and discuss this in the revised manuscript. For purpose of transparency, we also included a plot in the Supplementary (Page 19 Figure S11A) that explicitly shows logging as a function of time.

methods:

"Finally, **6)** we conducted various sensitivity tests to ascertain whether imbalanced/missing data could have an impact on the results. As there were several infrequent loggers (n = 17 that logged only 1 symptom throughout the testing period) and one particularly high frequency logger (total logs = 175) – see Figure S1B, we redid our analysis removing these participants (Figure S10). Next, we found that frequency of logging decreased over time (Figure S11) with 119 participants logging symptoms on Day 1, but by Day 8 this had diminished to 38 loggers, with Day 14 only having 7 loggers left. Thus, we checked the strength of effects in Week 1 (Days 1-7) vs Week 2 (Days 8-14) to assess whether the decrease in data points impacted the results (Figure S11)." – Page 24 – 25 Lines 760 – 770.

Supplement:

"When removing high (n = 1) and low frequency (n = 17) loggers, we found that OCD severity (Odds Ratio = 1.29, 13 = 0.25, p = .024) and self-confidence (Odds Ratio = 0.73, 13 = -0.32, p = .008) still significantly predicted future symptom logging – Figure S10A.

Additionally, metacognitive bias, but not other task measures, still significantly predicted a future symptom log (Odds Ratio = 0.74, 13 = -0.31, p = .016) – Figure S10B.

A future symptom log, but not past logging, was still significantly associated with current self-confidence (Future log: 13 = -0.18, 95% CI [-0.27 -0.1], p < .001; Past log: 13 = 0.0037, 95% CI [-0.08 0.09], p = .93; Figure S10C) and metacognitive bias (Future log: 13 = -0.233, 95% CI [-0.43 -0.04], p = .019; Past log: 13 = -0.233, 95% CI [-0.18 0.16], p = .90; Figure S10D)." - Supplementary, Pages 18 Lines 295 - 304

For the split-half analysis, we found that our results were maintained in Week 1:

"In the first week of the study (Days 1 -7), controlling for other state measures, state self-confidence was still significantly predictive of a future symptom log (Odds Ratio = 0.58, 13 = -0.55, p < .001, Figure S11B), while the effect was in the same direction for metacognitive bias (controlling for other task measures), albeit weaker (Odds Ratio = 0.77, 13 = -0.26, p = .051, Figure S11C). However, both effects had disappeared by week 2/Days 8-14 (State self-confidence: Odds Ratio = 1.29, 13 = 0.26, p = .26, Figure S11D; Metacognitive bias: Odds Ratio = 0.46, 13 = -0.77, p = .25, Figure S11E), likely due to the

limited availability of symptom log data (see Fig S11A)." – Supplementary Page 18-19 Lines 314 - 322

We also briefly summarise these findings in our Main Results section:

"In addition to robustness tests described above, we conducted several sensitivity analyses targeting potential imbalances in the data and found that the main findings were largely robust across these checks. First, we found that when removing 17 low frequency loggers (i.e., those who logged only once across the entire study period) and 1 very high frequency logger (logs across study period = 175), self-confidence (Odds Ratio = 0.73, 13 = -0.32, p = .008) and task metacognitive bias (Odds Ratio = 0.74, 13 = -0.31, p = .016) still significantly predicted future symptom logging, suggesting that our results are not driven by outlier participants."

"Finally, we addressed the fact that there was a decline in symptom logging over the 14-day period (see Supplementary for visualisation). Specifically, symptom entries decreased from 119 participants on Day 1 to 38 by Day 8 and only 7 by Day 14. When restricting the analysis to the first week (Days 1–7), state self-confidence remained a significant predictor of subsequent symptom logging (Odds Ratio = 0.58, 13 = – 0.55, p < .001), and metacognitive bias showed a similar—though weaker—effect (Odds Ratio = 0.77, 13 = −0.26, p = .051). However, both effects were no longer significant during Days 8–14 (state self-confidence: Odds Ratio = 1.29, 13 = 0.26, p = .26; metacognitive bias: Odds Ratio = 0.46, 13 = −0.77, p = .25), likely due to the substantial reduction in available data rather than a true absence of effect. Thus, our conclusions hold under sufficient data density but may become unreliable when symptom logging rates are markedly low." – Page 13 Lines 357 - 382

Moreover, we outline possible mitigations against missing data in the Discussion:

"Nonetheless, we did demonstrate that our results rely on having enough participants logging to demonstrate that reduced confidence precedes symptoms, as the effect was weakened in the 2nd week of the study when substantially less people were logging symptoms. Thus, future work should design techniques for improving compliance for self-directed reporting. One method could be to notify participants, at the end of the day, to explicitly record whether they did NOT experience any symptoms that day, and to otherwise retrospectively log them. This method additionally aids in distinguishing between a true lack of symptoms and non-compliance.." – Page 16 Lines 485 - 493

2.2 Additionally, the decision to retain only the first symptom log when multiple logs were recorded in close timing may distort the temporal signal. It is unclear how this choice affects the interpretation of momentary symptom fluctuations, especially if

participants tended to log multiple events retrospectively (several at the end of a day).

We thank the reviewer for raising this point, but this might be based on a misunderstanding. Indeed, for the logistic mixed effect models, we binarized the symptom logs, such that it the symptom log variable is defined as the "presence [1] or absence [0] within a 4-hour time window from the completion of a state or task notification." (Page 20 Line 625 - 626). Hence, any symptom logs, after the first one, recorded within the 4-hour period are not additionally accounted for by the models.

This method enabled us to account for imbalanced data, as several participants did not log frequently enough to fit a model that accounts for all data (for instance, a linear model with the dependent variable specified as the <u>total number of symptom logs</u> within a 4-hour period). We have now made this more explicit in our Method section:

"The method of binarizing the symptom log variable in our mixed models partially accounts for imbalanced data because several participants simply did not log frequently enough to operationalise symptom severity as the *total number* of symptom logs within a 4-hour period from state notification. However, treating the symptom log as a binary variable led to us retaining only the first symptom log close to notification completion and discarding symptom logs that were recorded after." Page 22 Lines 685 – 6690

We checked if this could be a potential issue by assessing the number of logs discarded per participant. This is outlined in our revised Methods:

"We found that several participants had <u>no</u> logs discarded (n = 45; 38% of our sample), while the median number of logs discarded across all participants was 1 (interquartile range = 4) – see Figure S8 for visualisation" – Page 22 - 23 Lines 692 - 694

Our supplementary Figure S8 (page) also highlights this more clearly:

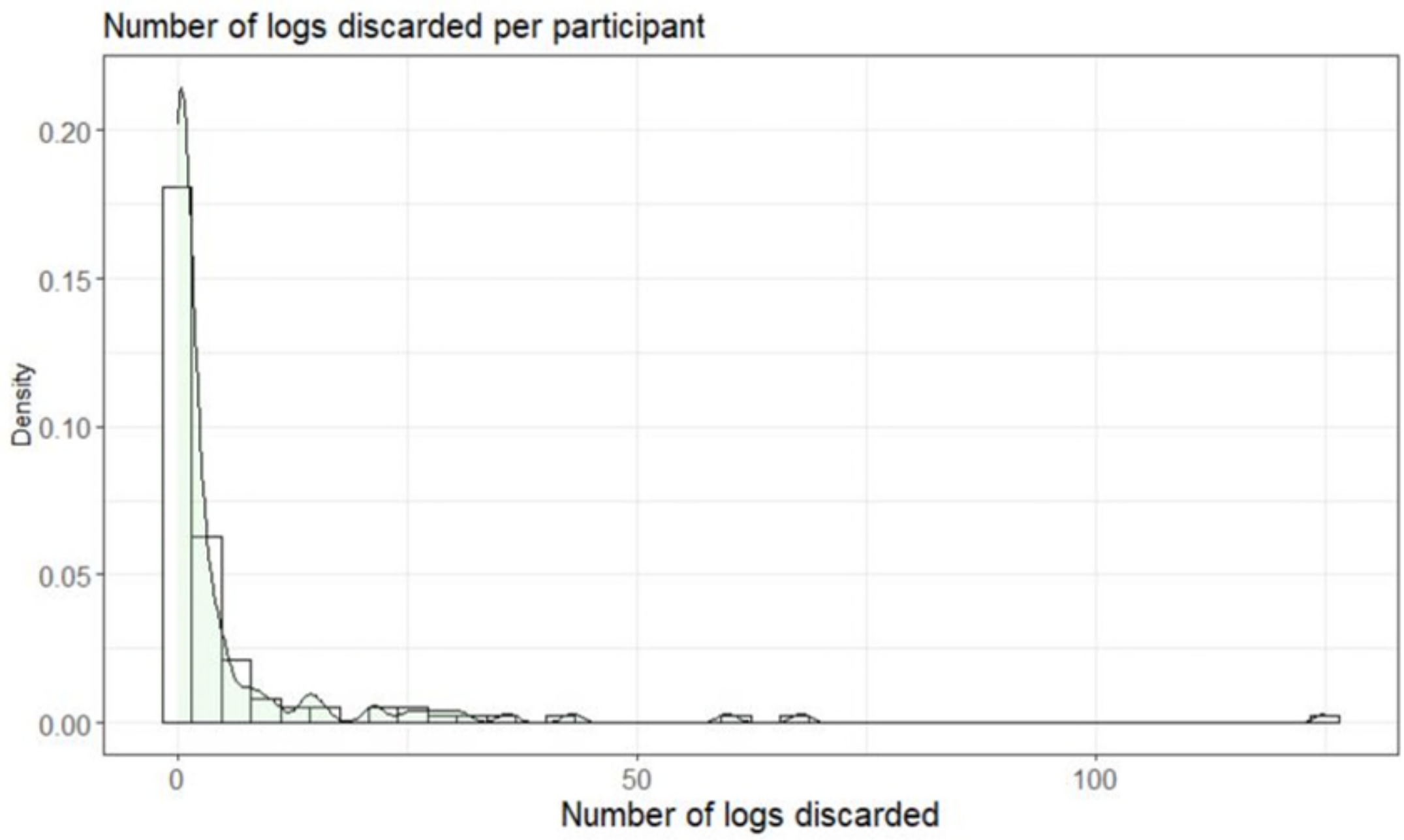

Nonetheless, we also include a caveat right after in the Methods:

"Considering the relatively low proportion of data loss, we determined that logistic regression models were suitable for our analysis. However, we recognize that in samples with greater logging frequency, a linear model that retains all data would be preferable."-Page 23 Lines 694 - 697

The retrospective logging should not impact the results here as when participants do log retrospectively, they include the approximate time of day that they experienced a symptom(s). Thus, the retrospective log is inserted alongside the appropriate time/day column of our dataset. We describe this in our revised Methods:

"They were also able to retrospectively log symptoms, an option we provided to account for their symptoms of OCD interfering with their ability to log symptoms at the specific moment. To do this, in response to the question above, they would select the option 'No, but I did earlier today that I would like to report'. They would then be able to select an approximate time (in 30-minute intervals) that they last experienced symptoms." – Page 20, Lines 598 - 603

And we have now included, briefly, that these logs are analysed the same as real-time logs:

"These retrospective logs were then placed into the appropriate timeframe in the dataset and were analysed alongside real-time logs". (Page 20 Lines 603 - 604)

2.3 Including a random intercept for each subject is essential to account for within-person dependence. The authors should report whether random effects were

significant. At present, the absence of this information makes it difficult to assess model validity.

We agree that accounting for within-person dependence is essential and our original analyses already included a random intercept for each participant. We now explicitly report the estimated variance and standard deviation of this random effect and we ran likelihood-ratio comparisons with models with and without the subject random intercept, which supports its inclusion.

This is described in our Methods:

"We assessed that accounting for within-person dependence improved on model fit (or otherwise showed no difference in performance) compared to non-mixed effect models by reporting the estimated variance and standard deviation of the random effects. We also ran likelihood ratio tests comparing models with and without the random intercept (see Supplementary and Table S7)." - Page 22 Lines 670 – 674.

We include the supporting results in the Supplementary:

"When assessing significance of random subject-intercepts in our main models (see Table S7 for statistics), we found that the addition of per-subject intercepts improved the fit of models where 'Future Symptom Log' was inserted as the dependent variable. Introducing a random intercept did not enhance the performance of models that used future and past symptom logging to predict state variables. Since beta estimates were similar between models with and without the random intercept, we decided to retain our original results. Moreover, we ascertain that our findings remain significant even when fitting models without random intercepts." – Supplementary Page 14 - 15, Lines 255 - 266

Table S7: Results comparing main models with and without the random subject-intercept

Model	Variance	Standard Deviation	Likelihood Ratio Test	Random-Intercept improved model performance?
Current OCD Severity$_{t,i}$ ~ Future Symptom Log$_i$ + Past Future Log$_i$	1.17e-32	1.082e-16	$\chi^2(1) = 0$, $p = 1$	No difference.
Future Symptom Log$_{i,t} \in \{0,1\}$ ~ Self-Confidence $_{t,i}$ + Other State Variables$_{t,i}$	2.72	1.51	$\chi^2(1) = 161.98$, $p < .001$	Improved
Current Self-Confidence$_{t,i}$ ~ Future	9.256e-33	9.621e-17	$\chi^2(1) = 0$, $p = 1$	No difference.

Symptom Log_i + Past Future Log_i				
Future Symptom $Log_{i,t} \in \{0,1\}$ ~ Task Metacognitive $Bias_{t,i}$ + Other Task Measures $_{t,i}$	2.29	1.51	$\chi^2(1) = 60.16$, $p < .001$	Improved.
Task Metacognitive $Bias_{t,i}$ ~ Future Symptom Log_i + Past Future Log_i	0	0	$\chi^2(1) = 0$, $p = 1$	No difference.

In addition to this, we also check that our results hold when controlling for random slopes per subject (see Supplementary Page 16-17 Table S8) to account for potential subject variability in the relationships (slopes) between variables.

2.4 A more detailed description of study compliance (response rates over time, dropout, timing of task completion) would also clarify data quality. The reported 50% completion threshold for inclusion may be too low, adopting a stricter cutoff could reduce variability and improve reliability.

Thank you for this query. In our response to this reviewer's earlier query, we have now added plot explicitly showing the number of participants logging symptoms over time (Figure S11A, Supplementary Page19). This clarifies the dropout rate in logging alongside Figure S1A-B (Supplementary Page 2) that reports the dropout rate and total number of symptoms logged per participant.

To address the concern that the 50% completion threshold might be too low, we now report redoing our main analyses under a more stringent 75% threshold, which retained 101 out of 175 subjects (a 50% completion rate threshold retained 137/175). Critically, our findings also hold under these more conservative criteria. We now report this in our revised manuscript.

"Moreover, our decision to retain participants who completed at least 50% of notifications may be viewed as too liberal, and hence we reattempted our analyses using a more stringent 75% cut-off. We found that lowered state self-confidence, (controlling for other state measures; 13 = -0.61, Odds ratio = 0.76, p = .0014) and metacognitive bias (controlling for other task measures; 13 = -0.29, Odds ratio = 0.75, p = .031) still significantly predicted a future symptom log."– Page 13 Lines 365 – 370

We also discuss the dropout rate in the Discussion:

"Next, as is typical in ecological momentary assessment (EMA) research, our dataset, particularly the symptom logs, exhibited unevenness and missingness, with some

participants completing entries infrequently or insufficiently. Given the high notification frequency (four times per day), we adopted a 50% completion threshold, which could be considered relatively liberal compared to other EMA studies[13]. Our primary findings remained robust despite these limitations, suggesting the presence of a genuine effect." – Page 16 Lines 479 - 485

2.5 Misplaced focus of sensitivity analyses

While the manuscript includes several sensitivity analyses (e.g., time-of-day and day-of-week effects), these appear overly detailed and somewhat tangential to the central questions of data robustness. They give the impression of a distraction from the more critical concerns, namely, the sparse and uneven distribution of OCD symptom events, the dominance of a few high-frequency participants, and the substantial amount of missing data. Sensitivity analyses should instead directly test whether the reported effects persist under alternative models, smaller/completer samples or resampling procedures that explicitly address these imbalances and missingness.

We appreciate the reviewers' suggestion to alter the focus and structure of our paper. Whilst we agree with the reviewers' comment about the 'sensitivity analyses' being detailed, we believe this is essential because we wanted to verify that the main findings will remain robust in the face of covarying for external predictors, like time-of-day, day-of-week, and activities participants are doing. Especially considering that, in our study, they are important precursors to when people log symptoms. Notably, established prior work has reported the importance of these factors in potentially driving OCD symptom fluctuations [12,14,] which means that it is important that we analysed (and controlled) for these effects.

Additionally, given the novelty of our methodology, it is important we carry out these extra tests to demonstrate proof-of-concept, in the sense that we can ascertain that participants are indeed logging meaningfully. We have done so similarly in our previous studies (e.g. Hewitt et al., 2025, PNAS), where this was much appreciated by the reviewers and readers.

Nonetheless, we take the reviewers' observation that the key results were relatively brief (also note Reviewer 1's comment R1.1) and their suggestion for conducting further analyses seriously. To address this, we have now added detail to these analyses, report further analyses in the main results rather than the Supplement, and we expanded our analyses, not least due to the many helpful suggestions of this reviewer. We believe that the revised results section thus provides a more balanced presentation of results where the key findings are shown in detail.

Please see the revised manuscript for the entire revised results section.

2.6 Insufficient model specification and lack of clarity on random effects The description of the statistical models in the main text is incomplete (and also not clear in the supplementals data). It is unclear which predictors and outcome variables were included, whether random effects for subjects were modelled, and how within-person correlations were handled. Given the repeated-measures design and the large heterogeneity in event frequency, inclusion of random subject effects is essential. The full model specifications, estimation methods, and justification for the chosen model structure should be reported in detail in the main text.

We apologise for a lack of clarity in our methods and have now substantially revised the methods section and added more detail about the model specifications.

Specifically, we have outlined the exact models used in our main analyses, covariates, and equations (in Wilkinson notation; for equations, see revised main manuscript) in the Methods:

"When analysing the impact of self-confidence on future symptom logs, we controlled for other state variables (OCD severity, anxiety, happiness, brain fog, and sleep quality, see Equation 1; Figure 3a). Next, when investigating the effect of metacognitive bias over symptom logging, we controlled for potential confounding task variables including staircased accuracy, choice reaction time, and signal strength (Equation 2; Figure 4c). Additionally, we checked that the relationship between metacognitive bias and symptom logging was maintained when controlling for state variables recorded close in time (≤ 4 hours) to task completion (OCD severity, anxiety, happiness, brain fog, and sleep quality) using a model similar to Equation 1 but with metacognitive bias inserted as an independent variable instead of self-confidence."– Page 22 Lines 675 - 684

"For cross-correlation analyses (i.e., to check how OCD severity, self-confidence and metacognitive bias affects past and future OCD symptoms), we created lagged and leading versions of the symptom log variables. Lagged and leading symptom log variables were inserted in the same logistic mixed effect model predicting 1) OCD severity (Equation 4; Figure 2e) 2) self-confidence (Equation 5; Figure 3a) and 3) metacognitive bias (Equation 6; Figure 4b)." – Page 23 Lines 711 - 716

We explicitly state random intercepts were modelled (see our response to review question 2.3 for a detailed discussion of the random effects):

"Logistic mixed effect models with random intercepts were used when 'symptom log' (presence [1] or absence [0] within a 4-hour time window from the completion of a state or task notification) was inserted as the dependent variable." – Page 22 Lines 667 - 670

Additionally, we describe analyses that use linear models without random intercepts (due to all variables already being within-person centred):

"To verify that self-confidence and metacognitive bias are indeed temporally interconnected, we fit two linear models with metacognitive bias as the dependent variable and self-confidence as the independent variable, while controlling for state variables (Equation 6) and for task variables (Equation 7). These models did not include random intercepts as all dependent and independent variables were already within-person centred." – Page 22 Lines 730 - 6735

Moreover, to account for potential subject variability in these relationships, for we also checked that our results hold when controlling for random slopes per subject (see Supplementary Page 15-16 Table S8).

Justification for controlling for other state variables in our model are outlined earlier in the Methods section:

"We tested for state anxiety[15,16], and happiness/mood[15,17] because OCD has high comorbidity rates with both anxiety and depressive disorders[18], and it is thus important to control for these variables when investigating the link between OCD and confidence. Similarly, we assessed sleep quality once a day due to the disorder being associated with sleep disturbances and reduced sleep efficiency[19].

To address the potential impact of poor concentration or diminished thought clarity resulting from OCD symptoms on performance in the perceptual metacognitive task, we included a 'brain fog' item designed to assess difficulties with mental clarity. Evaluating this aspect was considered important, as 'mind-blanking' is also observed during states of anxiety [20]." – Page 19 - 20 Lines 569 – 578

2.6 Furthermore, Equation 3 in the Methods section appears to be mis-specified. If the goal is to test how self-confidence influences past and future symptom logs, then the dependent variable should be the symptom log, not self-confidence. In other words, the equation should be reversed. Without this correction, the analysis instead tests how symptoms predict self-confidence, not vice versa.

We apologise for the lack of clarity on this point. Our basal goal was to test the association between self-confidence and symptom logs. The directionality of the effect comes automatically through the temporal succession of these events, i.e. because past symptom log did take place before a self-confidence rating there is a natural directionality – rather than through the mathematical model.

Critically, we wanted to test whether self-confidence was more closely related to past vs future symptom log. This means that these two measures should directly compete for

variance to establish which one is more tightly related. This is only possible in a model as specified in the original equation 3.

To our knowledge, this approach is widely used and accepted. We, for example, have used a similar approach in our previous work when working with ecological momentary assessment data (see Hewitt et al., 2025).

To be transparent about the approach and the rationale behind it, we have now clarified this in the revised methods section:

"For cross-correlation analyses (i.e., to check how OCD severity, self-confidence and metacognitive bias affects past and future OCD symptoms), we created lagged and leading versions of the symptom log variables. Lagged and leading symptom log variables were inserted in the same logistic mixed effect model predicting 1) OCD severity (Equation 3; Figure 2e) 2) self-confidence (Equation 4; Figure 3a) and 3) metacognitive bias (Equation 5; Figure 4b)." – Page 23 Lines 711 - 716

2.7 Finally, the section around line 636, which mentions a 'fitting procedure' is unclear: it is not specified what model was fitted or how this step relates to the primary analyses.

We apologise for the lack of clarity. The fitting procedure corresponds specifically to the estimation of metacognitive sensitivity, which was merely a secondary analysis we compared against metacognitive bias. To make this apparent, we have amended the subtitle of this section to be '**Metacognitive model fitting procedure'** and we have explicitly referenced metacognitive sensitivity in the first sentence of this section:

"Following previous work, for metacognitive sensitivity estimation, we adjusted the model parameters..." - Page 26 Line 806 - 807

2.8 Inadequate reporting of task-level data and metacognitive measures The calculation of metacognitive bias appears incorrect. The manuscript treats it as the mean of the confidence ratings, but conventionally, metacognitive bias (or calibration) is calculated as mean accuracy minus mean confidence. The authors should clarify this computation and revise terminology accordingly. As currently analyzed, the measure represents simple confidence in decisions, not bias.

We respectfully disagree with the reviewers' comment. Our operationalisation of metacognitive bias is entirely in line with the standard operationalisation used in cognitive neuroscience [11,21–24]. The foundational literature in the field has defined metacognitive bias as 'the overall level of confidence expressed, independent of whether the trial is correct or incorrect' [23].

Given that accuracy was staircased in our metacognitive paradigm (analogous to most cognitive neuroscience metacognition paradigms) and was thus held constant across sessions and participants, accuracy can be assumed as stable and hence the reviewer's suggestion would have led to very similar results.

In line with the literature upon we are building, we have decided to keep our original operationalisation in the paper as any change would only induce confusion to the reader.

2.9 Task performance data, including mean accuracy, confidence, and derived measures, should be reported at the <u>individual-participant level</u> with measures of variability (e.g., SDs or CIs). This would allow readers to <u>evaluate individual differences</u> and data reliability.

We thank the reviewers for this suggestion, and we have now added a section in the supplementary depicting plots summarising per subject and per session (8 in total) signal strength, mean confidence/metacognitive bias, mean choice reaction time, and meta-d'/d' (Figure S3, Supplementary page 4). A similar figure for staircased accuracy has also been added as a sub-figure in Figure 4 in the main text (page 12). Furthermore, we have included a table describing the numerical means and standard deviations per task measure per session (Supplementary Table S2, page 5).

2.10 Figure C, showing within-subject correlations between self-confidence and metacognitive bias, reveals very high variability, including a subset of participants showing negative correlations. These negative associations should be explicitly reported and discussed rather than implied visually.

We have now clarified in the Results that 57.3% of participants show a positive correlation (Spearman correlation coefficient ≥ 0.1) between metacognitive bias and self-confidence, and 29% show a negative correlation (Spearman correlation coefficient ≤ -0.1):

"We found a significant within-subject correlation between metacognitive bias and self-confidence (Figure 4b; median Spearman's rho = 0.24, one-sample Wilcoxon rank-sum test: $p < .001$, % participants showing a positive correlation coefficient ≥ 0.1 = 57%, participants showing a negative correlation coefficient ≤ -0.1 = 29%)" – Page 11, Lines 301 - 304

We discuss this variability briefly in the Discussion and make suggestions for future work:

"...our work has stopped short of addressing person-level heterogeneity in the relatedness between levels of metacognition: our findings uncovered a proportion of the sample (29%) displayed a negative relationship (correlation coefficient of ≤ -0.1) between metacognitive bias and self-confidence. Future work should address whether

this dissociation is meaningfully related to psychopathology and has implications for one's broader metacognitive processes. ." – Page 15 Lines 432 - 435

2.11 For transparency, the authors should also specify whether participants excluded from meta-d' analyses (due to insufficient trials) were also excluded from other analyses, or whether the sample size varies across reported effects.

We agree that this should be stated explicitly and have hence included the following sentence within our description of model fitting

"Considering the estimation of meta-d'/d' was conducted for secondary analysis and estimation of metacognitive bias (our task primary variable) does not require fitting this model and is robust to such distributional irregularities, we retained all sessions for analyses of metacognitive bias to avoid unnecessary loss of power." – Page 25 - 26 lines 802 - 805

2.12 Staircasing procedure and task data
The authors state that a staircase procedure was used to maintain ~70% accuracy across participants, but they do not present evidence that this was achieved. The mean accuracy and its standard deviation should be reported, as should verification that performance remained stable over time. Given that only 20 trials were used for the staircase, this may have been insufficient to converge on a reliable threshold. The lack of proof that the staircasing worked properly undermines claims that differences in confidence reflect metacognitive rather than performance.

We now have a figure in the main text showing the averaged staircased accuracy over 8 sessions (with individual participant variability included, Figure 4A). We also provide the following description to demonstrate the validity of our staircasing method:

"We ascertained that the staircasing procedure succeeded in producing an averaged (proportion) accuracy of 0.72 ± 0.005 across all participants and across 8 sessions (Figure 4A), within the range of average staircased accuracy reported in prior metacognitive studies[7,11,22,24]. Each individual session also yielded averaged accuracies ranging from 0.71 to 0.73." – Page 10 Lines 278 – 282

Furthermore, we have confirmed that practice effects (i.e., session number) do not substantially impact the staircased accuracy:

"Moreover, in a linear mixed-effects model with session number predicting staircased accuracy, we found that accuracy did not significantly fluctuate by session (β = 0.023, 95% CI [-0.055 0.009], p =.16) indicating that practice effects did not substantially impact participant accuracy." – Page 10 Lines 282 - 286

We also include the per session means and standard deviations for staircased accuracy in Table S2 of the Supplementary (Page 5).

2.13 Interpretation of temporal effects

The reported finding that future OCD symptom events predict task confidence better than past events is not particularly surprising, given that lag/lead analyses inherently favor forward prediction and struggle to interpret retroactive effects. The text around line 259 is also slightly confusing: "the other way around" refers to past symptoms, and this should be clarified for precision.

We are unsure about this comment and how to interpret it. As the reviewer has clearly noted, we allowed the two variables to directly compete for variance with the intention of assessing which one would be more predictive. In the model, there has been no 'favouring' of one variable over the other. We did not use more complex directional/causal models so as to maximise the explainability and to prevent possible effects, like the one mentioned, from happening.

We have clarified this and the confusing wording that this reviewer highlighted. For

self-confidence –

"We uncovered that the relationship between self-confidence and the likelihood of logging a symptom was unidirectional: in a linear mixed-effects model current self-confidence was significantly associated with a future symptom log (13 = -0.18, 95% CI [0.26 -0.10], *p* < .001; Figure 3b) but not with a past symptom log (13 = 0.007, 95% CI [-0.08 0.09], *p* = .88)." - Page 9 Lines 254- 258

For metacognitive bias -

"In line with our self-confidence findings, we saw that reduced metacognitive bias predicted future symptom logs (13 = -0.21, 95% CI [-0.40 -0.015], *p* = .035) but past symptom logs did not impact current metacognitive bias (13 = 0.03, 95% CI [-0.13 0.19], *p* = .69) – Figure 4d." - Page 12 Lines 336 - 339

2.14 Broader interpretational issues

In the discussion, the authors refer to the ongoing debate in the field regarding differences between clinical OCD populations and transdiagnostic samples of highly compulsive individuals. Previous work has shown that clinically diagnosed OCD patients typically display reduced confidence, whereas transdiagnostic or non-clinical compulsive samples sometimes show elevated confidence levels. However, it remains unclear what this study contributes to that debate. The authors use self-identified OCD participants who also exhibit low confidence, replicating earlier findings rather than clarifying the discrepancy between clinical and

transdiagnostic results. Stronger theoretical arguments are needed to explain how these findings advance understanding of the confidence–compulsivity relationship across diagnostic boundaries.

We agree with this notion and have now made it clear that instead of reconciling this debate, our results contribute to this growing body of knowledge:

"This EMCT study is the first to implement naturalistic self-initiated symptom logging throughout the assessment period, which enabled us to effectively ascertain the temporal relationship between multimodal metacognitive processes and OCD symptoms. Our work contributes to the growing cognitive OCD literature which has produced heterogeneous accounts regarding metacognition." – Page 14 Lines 394 - 398

Namely, by explicitly investigating temporal directionality:

"Thus, our EMCT design advances existing knowledge by providing i) insight into how these processes interact over time while controlling for potential confounding factors, and ii) assessment of the temporal directionality between processes. Using this state fluctuation approach, we established, for the first time, that it is reductions in confidence that precede OCD symptoms within the timescale of a few hours." – Page 14 Lines 404 - 406

We have also made it apparent that further research needs to be done to assess how and whether transdiagnostic and clinical samples diverge longitudinally:

"It is now crucial to understand whether clinical and subclinical/transdiagnostic populations are in alignment on these within-subject momentary associations between metacognition and OCD(-like) symptoms, and this may shed light on diverging cross-sectional findings thus far." -Page 14 Lines 412 - 415

2.15 Additional procedural and compliance questions

Several practical aspects of the EMA design remain unclear:

- **What happened if participants received the notification for the task during a busy moment, could they postpone it?**

Participants were informed that they should answer notifications within one hour of receiving the notification, otherwise the assessment would not count towards their completion. Further information about this has now been added to the Methods:

"Participants were reimbursed at a base rate for completing the screening and trait questionnaires (£5.25) and received additional payment at the end of the 14-day study based on the number of assessments completed: they were informed they could earn up to £15 extra if they completed 70% of assessments, and up to £20 extra if they completed 100% of assessments. Participants were informed that they had to complete

surveys/tasks within one hour of receiving the notification, otherwise the assessment would not count towards their completion rate." – Page 19 Lines 547 - 551

- **Was there any mechanism to verify adherence to timing instructions?**

In practice, any notifications that were completed too close in time to each other (less than one hour apart) were excluded from analysis:

"First, there were several instances where participants bulk completed notifications in one sitting. To account for this, in the initial 175 recruited participants, we removed data points that were too close in time to each other within each participant (> 1 hour). We always retained the first data point within a given hour (e.g., if notification A was completed first, and notification B was completed 5 minutes later, we would retain data from notification A and discard B)." – Page 21 Lines 638 - 643

We encouraged compliance by sending personalised reminders to participants if they missed too many notifications. This has now been added:

"Participants who had missed more than 2 notifications in a row also received personalised reminders from the experimenter over the Prolific messaging platform to encourage compliance." – Page 19 Lines 549 - 551

- **Were participants allowed to indicate explicitly that they experienced no symptoms during the day?**

No, but we acknowledge this would have improved interpretation of the data as currently an absence of symptom logs in a day is inferred as 'no symptoms' when indeed this might not always be the case. We have included this as a suggestion for future research in the Discussion:

"Thus, future work should design techniques for improving compliance for self-directed reporting. One method could be to notify participants, at the end of the day, to explicitly record whether they did NOT experience any symptoms that day, and to otherwise retrospectively log them. This method additionally aids in distinguishing between a true lack of symptoms and non-compliance." – Page 16 Lines 488 - 493

Clarifying these procedures would strengthen the interpretation of compliance and data validity.

2.16 Summary

In summary, while the study addresses an important and innovative question, it currently lacks sufficient methodological transparency and robustness. To strengthen the manuscript, the authors should:

1. Provide complete model specifications, including fixed and random effects.
2. Enhance transparency regarding study compliance, exclusions, and timing procedures.
3. Report participant-level data and perform sensitivity analyses that address uneven event distributions, dropout, and missingness.
4. Present clear validation of the task and staircase procedure, including accuracy metrics and convergence evidence.
5. Correct the metacognitive bias calculation and terminology, and report variability in within-subject correlations.
6. Clarify Equation 3 and ensure the direction of temporal prediction aligns with the stated hypotheses.
Only with these revisions can the reported associations be interpreted with confidence.

We thank the reviewers #2 and #3 for their kind words about our study and for their dedication to providing detailed, thoughtful, and helpful feedback. We hope that our answers and amendments above have sufficiently addressed all comments and concerns.

Reviewer #2 (Remarks on code availability):

2.17 There are also several concerns related to data reproducibility and the structure of the dataset itself.
The code is generally well-documented, with a README file that clearly explains how to install dependencies and run the analyses. However, the Notif_times.csv file, which appears to be required to reproduce the analyses from the provided R script, is missing. Without this file, most of the reported analyses cannot be replicated, limiting transparency and verification of the results.

We apologise for this oversight and have since uploaded the missing Notif_times.csv file and have uploaded the updated EMCT_analysis_final.R script that includes code with all the extra analyses conducted during the revisions.

2.18 We also inspected the dataset and observed that the data distribution is highly skewed: almost all participants reported symptoms on the first day, but by day 14 only about 10% continued logging. Sixteen participants reported only a single symptom over the 14-day period, while one participant logged as many as 175. This imbalance raises concerns about the representativeness of the results and the influence of high-frequency reporters. Notably, participants completed daily questionnaires consistently, suggesting that the decline is specific to symptom logging rather than general engagement.

We hope that our additional analyses as detailed above have adequately addressed the imbalanced data issue. We have also mentioned this in the limitations section of the revised Discussion.

2.19 Finally, aside from the issue with Equation 3 (which should be reversed to test directional effects properly), the main analyses appear appropriate. Nevertheless, the analysis script includes many side analyses not directly related to the primary research question, which may dilute the focus and could be moved to supplementary materials for clarity.

In addition, we hope that we have sufficiently argued for the suitability of our directional analysis above.

<u>Reviewer #3 (Remarks to the Author):</u>

3.1 I co-reviewed this manuscript with one of the reviewers who provided the listed reports. This is part of the Nature Communications initiative to facilitate training in peer review and to provide appropriate recognition for Early Career Researchers who co-review manuscripts.

We thank Reviewer #3 for their comments and suggestions in concert with Reviewer #2.

References

1. Baird, B., Smallwood, i., Gorgolewski, K. i. & Margulies, D. S. Medial and lateral networks in anterior prefrontal cortex support metacognitive ability for memory and perception. *J. Neurosci.* **33**, 16657–16665 (2013).

2. Saccenti, D. *et al.* Neural correlates of metacognition: Disentangling the brain circuits underlying prospective and retrospective second-order judgments through noninvasive brain stimulation. *J. Neurosci. Res.* **102**, 1–18 (2024).

3. Robbins, T. W., Vaghi, M. M. & Banca, P. Obsessive-Compulsive Disorder: Puzzles and Prospects. *Neuron* **102**, 27–47 (2019).

4. Vaghi, M. M. *et al.* Specific Frontostriatal Circuits for Impaired Cognitive Flexibility and Goal-Directed Planning in Obsessive-Compulsive Disorder: Evidence From Resting-State Functional Connectivity. *Biol. Psychiatry* (2017) doi:10.1016/j.biopsych.2016.08.009.

5. Bennett, D., Silverstein, M. S. & Niv, Y. The Two Cultures of Computational Psychiatry. *JAMA Psychiatry* **76**, 563–564 (2022).

6. Kashyap, H. *et al.* Cognitive training for neurocognitive and functional impairments in obsessive compulsive disorder: A case report. *J. Obsessive. Compuls. Relat. Disord.* **23**, 100480 (2019).

7. Katyal, S., Huys, Q. i. M., Fleming, S. M. & Dolan, R. i. Distorted learning from local metacognition supports transdiagnostic undercon fi dence. *Nat. Commun.* 1–16 (2025) doi:10.1038/s41467-025-57040-0.

8. Radomsky, A. S. *et al.* When it's at: An examination of when cognitive change occurs during cognitive therapy for compulsive checking in obsessive-compulsive disorder. *J. Behav. Ther. Exp. Psychiatry* **67**, 101442 (2020).

9. ielinek, L., Faissner, M., Moritz, S. & Kriston, L. Long-term efficacy of Metacognitive Training for Depression (D-MCT): A randomized controlled trial. 245–259 (2019) doi:10.1111/bjc.12213.

10. Tilley, P. i. M. & Rees, C. S. A clinical case study of the use of ecological momentary assessment in obsessive compulsive disorder. *Front. Psychol.* **5**, 1–8 (2014).

11. Seow, T. X. F., Fleming, S. M. & Hauser, T. U. Metacognitive biases in anxiety-depression and compulsivity extend across perception and memory. *PLOS Ment. Heal.* **2**, e0000259 (2025).

12. Nota, i. A., Gibb, B. E. & Coles, M. E. Obsessions and time of day: A Self-Monitoring Study in Individuals With Obsessive-Compulsive Disorder. *J. Cogn. Psychother.* **28**, 134–144 (2014).

13. Fritz, i. *et al.* So You Want to Do ESM ? 10 Essential Topics for Implementing the Experience-Sampling Method. (2024) doi:10.1177/25152459241267912.

14. Brierley, M. E. E. *et al.* How do daily routines and situational factors affect the severity of obsessive-compulsive disorder? *J. Psychiatr. Res.* **143**, 395–399 (2021).

15. Matcham, F. *et al.* Remote assessment of disease and relapse in major depressive disorder (RADAR-MDD): A multi-centre prospective cohort study protocol. *BMC Psychiatry* **19**, 1–11 (2019).

16. Starr, L. R. & Davila, i. Temporal patterns of anxious and depressed mood in generalized anxiety disorder: A daily diary study. *Behav. Res. Ther.* **50**, 131–141 (2012).

17. Heininga, V. E. *et al.* The dynamical signature of anhedonia in major depressive disorder: Positive emotion dynamics, reactivity, and recovery. *BMC Psychiatry* **19**, 1–11 (2019).

18. Lochner, C. *et al.* Comorbidity in obsessive-compulsive disorder (OCD): A report from the International College of Obsessive-Compulsive Spectrum Disorders (ICOCS). *Compr. Psychiatry* **55**, 1513–1519 (2014).

19. Paterson, i. L., Reynolds, A. C., Ferguson, S. A. & Dawson, D. Sleep and obsessive-compulsive disorder (OCD). *Sleep Med. Rev.* **17**, 465–474 (2013).

20. Andrillon, T., Lutz, A., Windt, i. & Demertzi, A. Where is my mind? A neurocognitive investigation of mind blanking. *Trends Cogn. Sci.* **xx**, 1–14 (2025).

21. Seow, T. X. F., Rouault, M., Gillan, C. M. & Fleming, S. M. How Local and Global Metacognition Shape Mental Health. *Biol. Psychiatry* **90**, 436–446 (2021).

22. Rouault, M., Seow, T., Gillan, C. M. & Fleming, S. M. Psychiatric Symptom Dimensions Are Associated With Dissociable Shifts in Metacognition but Not Task Performance. *Biol. Psychiatry* **84**, 443–451 (2018).

23. Fleming, S. M. & Lau, H. C. How to measure metacognition. *Front. Hum. Neurosci.* **8**, 1–9 (2014).

24. Fleming, S. M., Ryu, i., Golfinos, i. G. & Blackmon, K. E. Domain-specific impairment in metacognitive accuracy following anterior prefrontal lesions. *Brain* (2014) doi:10.1093/brain/awu221.

------------------------- END OF RESPONSE -------------------------

<u>Other Changes</u>

In addition to the amendments made based on reviewer comments, we have also made the following changes:

1) All figures now display statistical significance via explicit p-value ranges (e.g., “p < .001”) instead of asterisks (**) to improve comprehension of results.
2) The number of participants who had logged symptoms across the study was erroneously reported at 118 when it is in fact 119. This has been rectified across the manuscript.
3) The task diagram in Figure 1c in the main manuscript has been altered slightly to include an actual image of the stimuli from the task as prior to this, the alien images were shown as being bigger (and fewer) than what is typically shown to participants. The revised figure now better captures what study participants actually view in the task.
4) We have amended the ethics number in the Methods to be ‘23505/001’ (page 18, line 520) as previously it was incorrect.
5) The lead author Aleya A. Marzuki has recently changed institutions, so her affiliations now include her new one (Department of Neuroimaging, Institute of Psychology, Psychiatry & Neuroscience, King’s College London, United Kingdom)

Enclosed is the revised manuscript with changes in red font, a clean revised manuscript, supplementary (marked up and clean), and edited figures. We thank you again for considering our manuscript for publication and look forward to your decision in due course.

Aleya A. Marzuki, PhD

Post-Doctoral Research Fellow
Department of Psychiatry and Psychotherapy,
Medical School and University Hospital,
Eberhard Karls University of Tübingen,
Tübingen, Germany

Dear reviewers,

In this new rebuttal, we have marked the reviewer's comments in **bold**, our responses are in standard text, and direct quotations from the manuscript in blue. All page numbers and line number refer to the marked-up main manuscript with changes in red, unless stated otherwise (i.e., they may refer to the Supplementary).

START OF RESPONSE

Reviewer #1 (Remarks to the Author):

1.1 I have now carefully read the rebuttal and the authors' additional analyses have addressed all my previous comments. I think the extra control analyses provide important nuance to the conclusions of the study for the readers.

We thank Reviewer #1 for their positive evaluation of our revised manuscript and are very grateful for their suggestions which have improved the study's clarity and transparency.

Reviewer #2 (Remarks to the Author):

2.1 We thank the authors for their careful and thorough revisions and for the effort they made to address the other reviewer's and our comments. We appreciate the additional analyses and clarifications that were added to the manuscript, as well as the detailed responses provided. Overall, these revisions have improved the manuscript and helped clarify several aspects of the analyses. That said, we still have a few remaining points that we would like the authors to address or clarify further.

We would like to thank Reviewers #2 for their favourable assessment of our revised manuscript, as well as for providing additional insight into how it can be further improved.

Uneven data distribution and participant-level reporting

2.2 We thank the authors for their response to this comment and for conducting additional sensitivity analyses to address the concerns regarding uneven data distribution and potential missingness. We appreciate the effort to replicate the main analyses after removing the highest-frequency logger and participants who logged only once, as well as the inclusion of the split-half analyses examining Week 1 versus Week 2. These additions improve the transparency of the analyses and help clarify the robustness of the reported effects.

However, we remain somewhat concerned that the issue of data skewness may not yet be fully addressed. While removing the single highest-frequency logger (175 logs) and participants who logged only once is a helpful first step, the distribution of symptom logs still appears highly uneven. As illustrated in Figure S1B, there remain several participants who logged only two symptoms, while others logged very large numbers (e.g., above 100). Consequently, the dataset may still be disproportionately influenced by a relatively small number of high-frequency reporters. We therefore encourage the authors to further discuss this imbalance and consider whether additional robustness checks might help demonstrate that the findings are not driven by this remaining skew.

We thank the reviewer for encouraging us to consider the possible data imbalances further. We have now conducted additional sensitivity analysis in which we removed participants showing extreme symptom log numbers according to the following standard statistical criterion: either above 75% or below the 25% quartiles by a factor of 1.5 times the interquartile range. This initially led to the removal of 12 participants who showed extremely high logs, although no participants in the lower ranges met the cut-off for removal (using the quartile method, the cut-off for high logging was 41.75, while the cut-off for low logging was -20.25). Nonetheless, as those who logged only once or twice may not be logging meaningfully, we also removed these infrequent loggers. This led to a further 28 participants removed, leaving 79 participants remaining in our sensitivity analysis.

We have now included a subplot showing the distribution of symptom log scores (where the skew, while still somewhat present, has now noticeably reduced) in our reconfigured Figure 4 in the main manuscript:

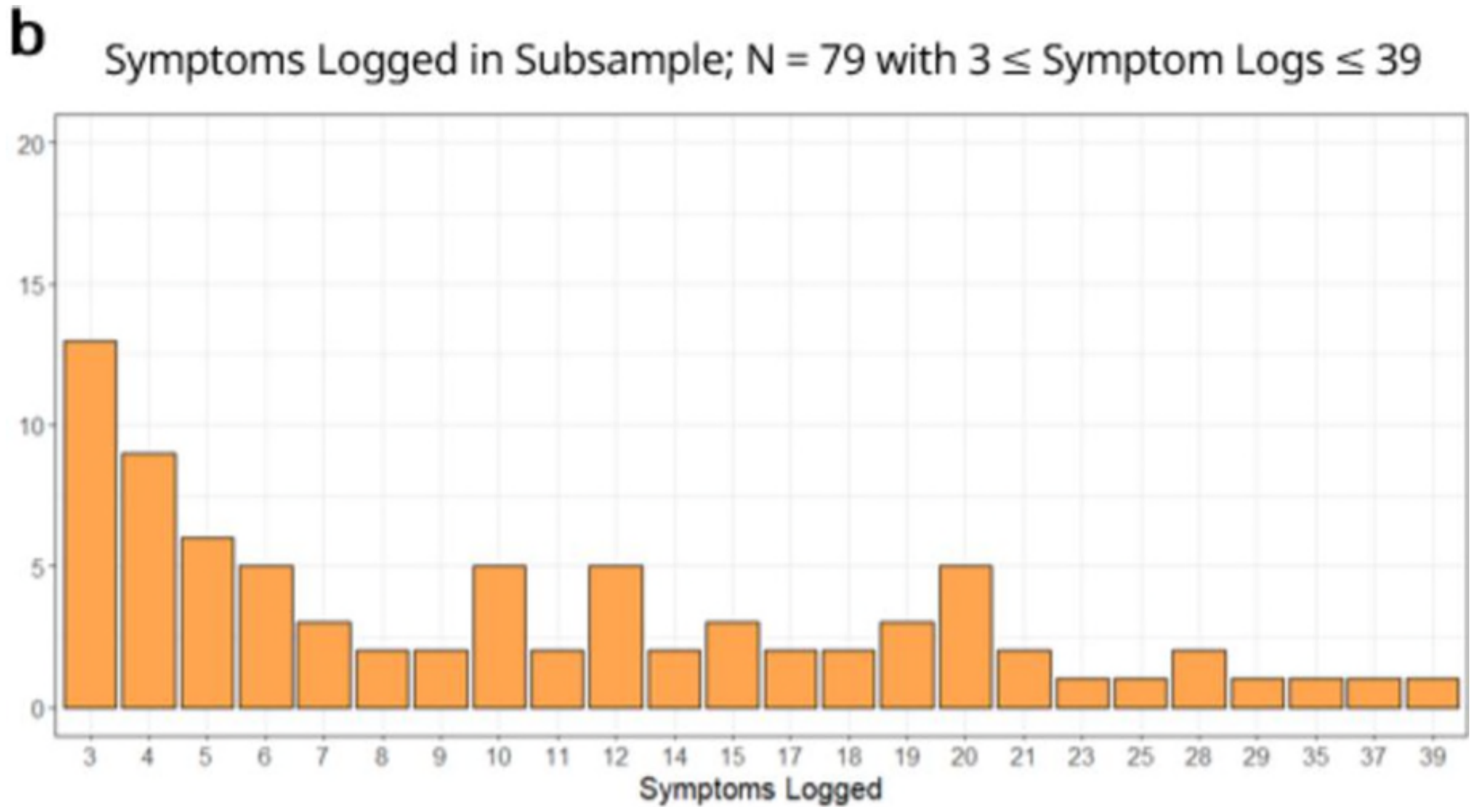

Critically, we found that our main results have remained stable in this subset of our participants, lending support to the robustness of our results. These new analyses are described in the manuscript's Results section:

"When removing the overly high- and low-frequency loggers, we found that self-confidence (Odds Ratio = 0.63, 13 = -0.46, p = .001) still significantly predicted future symptom logging – Figure 4b. Furthermore, a future symptom log, but not past logging, was still significantly associated with current self-confidence (Future log: 13 = -0.25, 95% CI [-0.36 -0.15], p < .001; Past log: 13 = -0.01, 95% CI [-0.12 0.10], p = .82; Figure 4d). Additionally, metacognitive bias, but not other task measures, still significantly predicted a future symptom log (Odds Ratio = 0.68, 13 = -0.39, p = .01; Figure 4e). The relationship between metacognitive bias and a future symptom log was similarly intact (Future log: 13 = -0.32, 95% CI [-0.56 -0.08], p = .0088; Past log: 13 = -0.01, 95% CI [-0.21 0.18], p = .90; Figure 4f)." – Page 14 - 15 Lines 397 - 406

Plots showing these results have been included in our new Figure 4 in the main manuscript on page 14.

We hope that these more restricted analyses credibly support the notion that our findings are not driven by potentially anomalous participants.

2.4 Relatedly, because the distribution of symptom logging across participants is central to evaluating the robustness of the results, we suggest that the figure illustrating this distribution (currently Figure S1B in the Supplementary) may be better placed in the main manuscript rather than the Supplementary. Presenting this information more prominently would improve transparency and allow readers to more easily assess the structure of the dataset.

We are happy to move this figure to the main manuscript and have thus placed it in our newly reworked Figure 4 (Figure 4b, page 14):

2.5 Regarding the split-half analyses, we agree that examining Week 1 and Week 2 separately is a useful approach given the decline in symptom logging over time. However, we find the interpretation that the disappearance of effects in Week 2 is "likely due to limited power rather than a true absence of effect" somewhat strong. While reduced sample size and data density could certainly contribute to the lack of statistical significance, the current analyses do not allow one to clearly distinguish between insufficient power and a genuine weakening or absence of the effect later in the study. We therefore suggest that the authors frame this interpretation more cautiously in the manuscript.

We thank the reviewer for challenging this interpretation. Indeed, our study design cannot distinguish between a power issue or a true absence of effect. We have now reframed this interpretation more tentatively in our Results section:

"However, both effects had disappeared by week 2/Days 8-14 (State self-confidence: Odds Ratio = 1.29, 13 = 0.26, p = .26, Figure 5D; Metacognitive bias: Odds Ratio = 0.46, 13 = -0.77, p = .25, Figure 5E), potentially due to the limited availability of symptom log data. Thus, our conclusions hold under sufficient data density, and future work with higher compliance rates will be better positioned to determine whether the this reflects a power limitation or whether the effect may genuinely attenuate over time." – Page 15 Lines 423 – 428.

We have also added this important caveat in our Discussion:

"Nonetheless, we did demonstrate that our results may rely on having enough participants logging to demonstrate that reduced confidence precedes symptoms, as the effect was weakened in the 2nd week of the study when substantially less people were logging symptoms. Albeit do note that our study design does not allow us to ascertain whether the lack of significance is due to insufficient power or a genuine absence of effect later in the study; it is uncertain whether repeated engagement with the app may have reduced the sensitivity of confidence ratings and symptom logs as measures, independent of compliance rate" -Page 19 Lines 538 - 546

Study compliance and completion threshold

2.6 We thank the authors for their response and for adding additional analyses and visualisations addressing study compliance. In particular, we appreciate the inclusion of the plot showing the number of participants logging symptoms over time, as well as the additional analysis using a stricter 75% completion threshold. These additions help clarify aspects of participant engagement and provide useful robustness checks.

We thank the reviewer for acknowledging our efforts to address study compliance, and we agree that this is a very crucial element to consider when conducting

ecological momentary assessment research.

2.7 However, we remain somewhat uncertain about how "completion" is defined in this context. From the description provided, it appears that completion refers primarily to whether participants responded to the prompted task notifications. However, an important component of the study design is the self-directed symptom logging. The current analyses therefore leave some ambiguity as to whether participants who met the completion threshold also continued to log symptoms throughout the study period, or whether some participants may have continued completing the prompted tasks while no longer reporting symptoms. Given that symptom logging itself is a central outcome variable in the analyses, this distinction is important for interpreting compliance and data quality. It would therefore be helpful if the authors could clarify how completion was operationalised and whether symptom logging behaviour was considered as part of the completion criteria.

We apologise for not being clearer on the definition of completion in the context of the study. We defined this as completion of surveys and tasks during timed notifications, and not symptom logging, as (given the lack of research investigating daily self-directed reporting in OCD) we did not have baseline expectations for how often participants would log their symptoms. We have made this explicit in our Methods section (see below), and we hope that our sensitivity analyses above have demonstrated that our results hold even when we *do* implement a minimum threshold for symptom logging.

"Next, we excluded participants (38 out of 175 recruited participants) who did not provide data ≥ 50% of the total timepoints. The final sample (N = 137) represented 78.3% of the recruited sample (see participant recruitment and exclusion flowchart in Supplementary). <u>This exclusion criteria applied only to timed notifications (i.e., for surveys and tasks) because we did not have prior expectations over how often participants would conduct self-initiated symptom logging. Hence, we did not impose an exclusion criterion based on symptom log frequency for our main analyses.</u> Nonetheless, we conducted sensitivity analyses on a subset of the sample that did not show extremely low and high logging frequency to ensure our findings were not driven by these few participants (see 'Sensitivity Analysis' sub-section in the Results)." -Page 23 - 24 Lines 704 - 710

2.8 Additionally, it is not entirely clear whether the analyses using the stricter 75% completion threshold were conducted on the full dataset or on the dataset after excluding the extreme loggers discussed in the previous point. Clarifying this would improve transparency. If these analyses were conducted on the full dataset, we would encourage the authors to also consider applying the stricter completion threshold in combination with the exclusion of extreme loggers, as

this may provide a more informative assessment of the robustness of the findings.

We agree that it would be important to assess whether the 75% completion threshold applied to the subset of data where we removed overly infrequent and frequent loggers. This has now been conducted and outlined in the main Results section:

"Next, our decision to retain participants who completed at least 50% of notifications may be viewed as too liberal, and hence we reattempted the analyses above (removing high and low frequency loggers) using a more stringent 75% cut-off (n = 101 before removing high/low frequency loggers, n = 56 after removal). We found that lowered state self-confidence, (controlling for other state measures; 13 = -0.41, Odds ratio = 0.66, p = .010) and metacognitive bias (controlling for other task measures; 13 = -0.46, Odds ratio = 0.63, p = .005) still significantly predicted a future symptom log." – Page 15 Lines 407 - 413

Reporting of task-level data and metacognitive measures

2.9 We thank the authors for their clarification regarding the operationalisation of metacognitive bias and for explaining how their definition aligns with the cognitive neuroscience literature. While we understand that the staircase procedure helps to ensure stable performance levels and can therefore be informative when assessing potential confidence biases (i.e., systematic deviations between subjective confidence and objective performance), it is important to note that confidence reports themselves do not constitute a bias. A bias implies a systematic misalignment between confidence and actual performance. For example, if participants report an average confidence of 70% and their performance is also around 70%, this would indicate good calibration rather than a bias. In the current manuscript, the term "confidence bias" appears to be used in places where the authors are in fact referring to confidence reports. Strictly speaking, this is conceptually inaccurate unless the analysis demonstrates a deviation between confidence and performance. To make this interpretation correct, the authors would need to explicitly quantify and report the relationship between confidence and accuracy.

We thank the reviewer for pressing this important point. On reflection, we agree. The term "bias" conventionally implies a systematic and quantifiable deviation from a normative reference point. Because of the staircasing, the experimentally measured variance in mean confidence ratings is not confounded by variance in accuracy, although staircasing alone does not introduce a reference point by which a conventional "bias" can necessarily be calculated. The Fleming tradition (Fleming et al., 2012; Fleming & Lau, 2014; Katyal et al., 2025; Rouault et al., 2018; Seow et al., 2026; Weil et al., 2013) has used "metacognitive bias" to refer to this quantity under controlled performance conditions, but this follows a terminological convention and the reviewer is right in that it may suggest connotations that the measure does not

actually implement. We have therefore revised the manuscript to make our operationalisation explicit:

"From the metacognition task, we derived two commonly studied measures: (i) metacognitive bias (operationalised as mean confidence rating[30]), where low bias indicates underconfidence while high bias indicates overconfidence and (ii) metacognitive efficiency (meta-d'/d'), derived using signal-detection theoretic computational models[31], which quantifies whether task confidence ratings are sensitive to correct and error trials while controlling for performance (d'). Here, metacognitive bias was defined according to metacognition research conventions[9,27,29,30,32,33], where it refers to the overall expressed confidence under staircased accuracy conditions – not to the calibration bias in the strict sense of a deviation from a normative reference point." -Page 10 Lines 282 - 291

Beyond terminological clarification, we also implement the reviewer's suggestion and conducted the analysis as they defined it: using the signed difference between confidence and accuracy as a bias measure. The results held:

"Finally, to address the possibility that the observed effects reflect a systematic miscalibration rather than an overall confidence level, we reanalysed the data using the signed difference between confidence and accuracy (confidence − accuracy). This alternative operationalisation is conceptually stricter as it uses accuracy as an explicit reference point against which confidence is evaluated. The results held: this calibration-based bias measure similarly showed a negative association with future symptom logs (Odds Ratio = 0.75, 13 = -0.28, *p* = .022), even when controlling for choice reaction times and signal strength (Odds Ratio = 0.78, 13 = -0.25, *p* = .046). This indicates that participants who are underconfident relative to their actual performance are more likely to subsequently report OCD symptoms." – Page 11- 12 Lines 338 - 347

The reviewer's suggestion has sharpened the conceptual framing and made the empirical results more robust and straightforwardly interpretable.

References

Fleming, S. M., Dolan, R. J., & Frith, C. D. (2012). Metacognition: Computation, biology and function. *Philosophical Transactions of the Royal Society B: Biological Sciences*, *367*(1594), 1280–1286. https://doi.org/10.1098/rstb.2012.0021

Fleming, S. M., & Lau, H. C. (2014). How to measure metacognition. *Frontiers in Human Neuroscience*, *8*(JULY), 1–9. https://doi.org/10.3389/fnhum.2014.00443

Katyal, S., Huys, Q. J. M., Fleming, S. M., & Dolan, R. J. (2025). Distorted learning from local metacognition supports transdiagnostic undercon fi dence. *Nature Communications*, *July 2023*, 1–16. https://doi.org/10.1038/s41467-025-57040-0

Rouault, M., Seow, T., Gillan, C. M., & Fleming, S. M. (2018). Psychiatric Symptom Dimensions Are Associated With Dissociable Shifts in Metacognition but Not

Task Performance. *Biological Psychiatry*, *84*(6), 443–451. https://doi.org/10.1016/j.biopsych.2017.12.017

Seow, T. X. F., Jelinek, L., Moritz, S., & Hauser, T. U. (2026). Integrating cognitive neuroscience and clinical perspectives on metacognitive mechanisms in psychopathology. *Nature Reviews Psychology*, *5*(January), 9–28. https://doi.org/10.1038/s44159-025-00503-4

Weil, L. G., Fleming, S. M., Dumontheil, I., Kilford, E. J., Weil, R. S., Rees, G., Dolan, R. J., & Blakemore, S. J. (2013). The development of metacognitive ability in adolescence. *Consciousness and Cognition*, *22*(1), 264–271. https://doi.org/10.1016/j.concog.2013.01.004

2.10 Additionally, if a bias is present, it would be important to specify its direction, as confidence biases can reflect either overconfidence or underconfidence. Based on the description of the results, the pattern seems more consistent with underconfidence rather than overconfidence. At minimum, the manuscript should clarify this distinction in the Method section and briefly indicate the direction of the bias.

Indeed, the pattern of results do point to underconfidence, specifically, preceding symptom reporting. The directions have now been clarified, as above, in the Results section:

"From the metacognition task, we derived two commonly studied measures: (i) metacognitive bias (operationalised as mean confidence rating[30]), where low bias indicates underconfidence while high bias indicates overconfidence..." -Page 10 Lines 283 - 285

Additionally, we have made it clearer in our Results that a lower metacognitive bias (preceding symptom logs) refers to underconfidence:

"When inserted into a logistic mixed effects model, lower metacognitive bias (i.e., underconfidence; Odds ratio = 0.75, β = -0.28, p = .022)." -Page 11 Lines 319 - 320

Minor points

2.11 There appear to be some inconsistencies in the numbering of the supplementary figures. The manuscript refers to Figure S11A in the Supplementary Materials, but in the document this figure appears to be labeled Figure S4, while another figure is already labeled S4. Similarly, there appear to be two figures labeled S3. We recommend carefully checking the numbering of all supplementary figures to ensure consistency with the references in the text.

We apologise for possible inconsistencies that arose from our revision. We have now carefully checked through our revised manuscript and supplementary to ensure consistency across both when referring to the figures.

2.12 Additionally, the figure illustrating the decline in symptom logging over time (referred to as Figure S11A, currently labeled S4A) is quite central to the discussion of compliance and missingness. The authors may want to consider whether this figure would be more appropriate in the main text rather than the Supplementary Materials, as it helps contextualize several of the sensitivity analyses discussed in the manuscript.

We agree that the figure would improve transparency further and indeed provide important contextualisation for the sensitivity analyses. Thus, we have now moved the entire Figure S11 to the main manuscript as a newly added main Figure 5 on page 16:

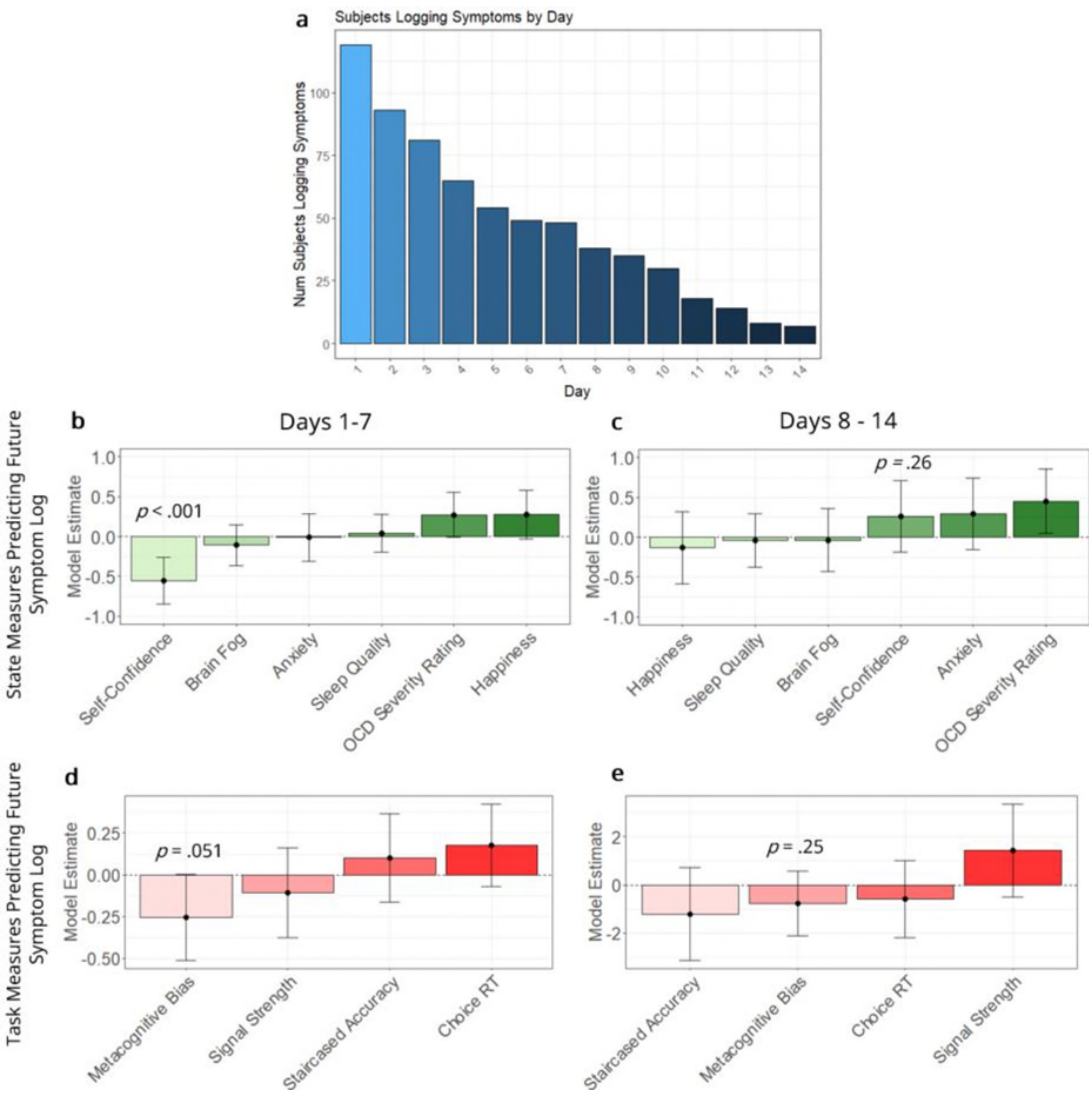

Reviewer #2 (Remarks on code availability):

2.13 Regarding scripts and data-analyses:
We appreciate that the authors have now uploaded the previously missing Notif_times.csv file and provided an updated analysis script including the additional analyses conducted during the revision. With the inclusion of this file, the analyses can now be reproduced, improving transparency and reproducibility.

We thank the reviewer for this acknowledgement; we agree that code reproducibility is a crucial aspect of open science initiatives, which we similarly champion.

Reviewer #3 (Remarks to the Author):

2.14 I co-reviewed this manuscript with one of the reviewers who provided the listed reports. This is part of the Nature Communications initiative to facilitate training in peer review and to provide appropriate recognition for Early Career Researchers who co-review manuscripts.

We thank both reviewers for their dedication to improving the quality and transparency of reported findings in our manuscript.

Other changes:

- The original Figures 3 and 4 (which depicted the main self-reported confidence and task results respectively) have now been combined into one figure (Figure 3, page 12) to enable the inclusion of two extra figures tied to our sensitivity analyses results (Figure 4, page 14; Figure 5, page 16).
- As all sensitivity analyses addressing compliance and missing data have been described fully in the main Results, and hence we have removed relevant sections from the Supplementary and Methods which were otherwise repetitive.
- We have uploaded an updated R script to our Github page: https://github.com/aleyaamarzuki/EMCT-OCD that includes the new analyses described here.

Enclosed is the revised manuscript with changes in red font, a clean revised manuscript (marked up and clean), supplementary, and edited figures. We thank you again for considering our manuscript for publication and look forward to your decision in due course.

Aleya A. Marzuki, PhD

Post-Doctoral Research Fellow
Department of Psychiatry and Psychotherapy,
Medical School and University Hospital,
Eberhard Karls University of Tübingen,
Tübingen, Germany
